# A novel electronic algorithm using host biomarker point-of-care tests for the management of febrile illnesses in Tanzanian children (e-POCT): A randomized, controlled non-inferiority trial

**DOI:** 10.1371/journal.pmed.1002411

**Published:** 2017-10-23

**Authors:** Kristina Keitel, Frank Kagoro, Josephine Samaka, John Masimba, Zamzam Said, Hosiana Temba, Tarsis Mlaganile, Willy Sangu, Clotilde Rambaud-Althaus, Alain Gervaix, Blaise Genton, Valérie D’Acremont

**Affiliations:** 1 Swiss Tropical and Public Health Institute, Basel, Switzerland; 2 University of Basel, Basel, Switzerland; 3 Boston Children’s Hospital, Boston, Massachusetts, United States of America; 4 Department of Ambulatory Care and Community Medicine, University Hospital Lausanne, Lausanne, Switzerland; 5 Ifakara Health Institute, Dar es Salaam, Tanzania; 6 Amana Hospital, Dar es Salaam, Tanzania; 7 Ilala Municipality, Dar es Salaam, Tanzania; 8 Médecins Sans Frontières, Geneva, Switzerland; 9 Pediatric Emergency Medicine Department, Child and Adolescent Medicine, Geneva University Hospital, Geneva, Switzerland; 10 Infectious Diseases Service, University Hospital Lausanne, Lausanne, Switzerland; Makerere University Medical School, UGANDA

## Abstract

**Background:**

The management of childhood infections remains inadequate in resource-limited countries, resulting in high mortality and irrational use of antimicrobials. Current disease management tools, such as the Integrated Management of Childhood Illness (IMCI) algorithm, rely solely on clinical signs and have not made use of available point-of-care tests (POCTs) that can help to identify children with severe infections and children in need of antibiotic treatment. e-POCT is a novel electronic algorithm based on current evidence; it guides clinicians through the entire consultation and recommends treatment based on a few clinical signs and POCT results, some performed in all patients (malaria rapid diagnostic test, hemoglobin, oximeter) and others in selected subgroups only (C-reactive protein, procalcitonin, glucometer). The objective of this trial was to determine whether the clinical outcome of febrile children managed by the e-POCT tool was non-inferior to that of febrile children managed by a validated electronic algorithm derived from IMCI (ALMANACH), while reducing the proportion with antibiotic prescription.

**Methods and findings:**

We performed a randomized (at patient level, blocks of 4), controlled non-inferiority study among children aged 2–59 months presenting with acute febrile illness to 9 outpatient clinics in Dar es Salaam, Tanzania. In parallel, routine care was documented in 2 health centers. The primary outcome was the proportion of clinical failures (development of severe symptoms, clinical pneumonia on/after day 3, or persistent symptoms at day 7) by day 7 of follow-up. Non-inferiority would be declared if the proportion of clinical failures with e-POCT was no worse than the proportion of clinical failures with ALMANACH, within statistical variability, by a margin of 3%. The secondary outcomes included the proportion with antibiotics prescribed on day 0, primary referrals, and severe adverse events by day 30 (secondary hospitalizations and deaths). We enrolled 3,192 patients between December 2014 and February 2016 into the randomized study; 3,169 patients (e-POCT: 1,586; control [ALMANACH]: 1,583) completed the intervention and day 7 follow-up. Using e-POCT, in the per-protocol population, the absolute proportion of clinical failures was 2.3% (37/1,586), as compared with 4.1% (65/1,583) in the ALMANACH arm (risk difference of clinical failure −1.7, 95% CI −3.0, −0.5), meeting the prespecified criterion for non-inferiority. In a non-prespecified superiority analysis, we observed a 43% reduction in the relative risk of clinical failure when using e-POCT compared to ALMANACH (risk ratio [RR] 0.57, 95% CI 0.38, 0.85, *p =* 0.005). The proportion of severe adverse events was 0.6% in the e-POCT arm compared with 1.5% in the ALMANACH arm (RR 0.42, 95% CI 0.20, 0.87, *p =* 0.02). The proportion of antibiotic prescriptions was substantially lower, 11.5% compared to 29.7% (RR 0.39, 95% CI 0.33, 0.45, *p <* 0.001). Using e-POCT, the most common indication for antibiotic prescription was severe disease (57%, 103/182 prescriptions), while it was non-severe respiratory infections using the control algorithm (ALMANACH) (70%, 330/470 prescriptions). The proportion of clinical failures among the 544 children in the routine care cohort was 4.6% (25/544); 94.9% (516/544) of patients received antibiotics on day 0, and 1.1% (6/544) experienced severe adverse events. e-POCT achieved a 49% reduction in the relative risk of clinical failure compared to routine care (RR 0.51, 95% CI 0.31, 0.84, *p =* 0.007) and lowered antibiotic prescriptions to 11.5% from 94.9% (*p <* 0.001). Though this safety study was an important first step to evaluate e-POCT, its true utility should be evaluated through future implementation studies since adherence to the algorithm will be an important factor in making use of e-POCT’s advantages in terms of clinical outcome and antibiotic prescription.

**Conclusions:**

e-POCT, an innovative electronic algorithm using host biomarker POCTs, including C-reactive protein and procalcitonin, has the potential to improve the clinical outcome of children with febrile illnesses while reducing antibiotic use through improved identification of children with severe infections, and better targeting of children in need of antibiotic prescription.

**Trial registration:**

ClinicalTrials.gov NCT02225769

## Abstract 2

In a randomized controlled non-inferiority trial, Kristina Keitel and colleagues compare the use of an electronic algorithm using host biomarker point-of-care-tests to routine care for the management of febrile illnesses in Tanzanian children.

## Author summary

**Why was this study done?:**

- Case management of children with infections remains inadequate, and antibiotic overprescription is a major challenge.
- To improve case management of children with acute infections, we developed an innovative tool: e-POCT, a smartphone-based algorithm that uses tests performed at the patient care site (point-of-care tests: oximetry, hemoglobin, C-reactive protein, and procalcitonin).
- Given the innovative nature of this algorithm, we assessed the safety of e-POCT in treating children with acute infections.

**What did the researchers do and find?:**

- In a randomized trial in Dar es Salaam, Tanzania, we compared the clinical outcomes of 1,586 children under 5 years with acute infections treated using e-POCT with that of 1,583 children treated using the current reference smartphone algorithm (ALMANACH).
- We observed that e-POCT improved clinical outcomes while reducing antibiotic prescription from 30% to 11%.
- e-POCT better targeted those children truly in need of antibiotic treatment through enhanced identification of children with severe diseases.

**What do these findings mean?:**

- e-POCT, an innovative smartphone algorithm using point-of-care tests, has the potential to improve clinical outcomes for children with acute infections and to increase the rational use of antibiotics.
- Future studies should assess e-POCT’s performance in routine care settings.

## Introduction

Febrile illnesses comprise the vast majority of pediatric outpatient consultations in resource-poor settings [1]. Only a small percentage of these children require antibiotic treatment or referral for hospital-based supportive care, such as oxygen therapy [2]. However, correct identification of this minority of children is pivotal; substandard management of children with infections has led to 2 major public health challenges: first, persistent high mortality from common childhood infections [3] and, second, the tremendous overprescription of antibiotics at the peripheral healthcare level [4], which contributes to spreading antimicrobial resistance [5].

Though childhood infections are common, their management requires integration of a multitude of information such as epidemiological, demographic, clinical, and laboratory data. It also necessitates the consideration of multiple diagnoses at once, as children often present with several concurrent complaints and symptoms [6]. Such an integrated approach for the classification and treatment of childhood infections is reflected in the current World Health Organization (WHO) strategy, Integrated Management of Childhood Illness (IMCI) [7]. Though a positive impact on child mortality could be shown [8], its implementation has faced major challenges through a spectrum of obstacles within the health system, from the macro (policy) to the micro (patient–provider interaction) level. At the micro level, the cornerstone of the IMCI strategy remains a set of paper-based algorithms that recommends presumptive treatment based on clinical signs and symptoms (except for the malaria rapid diagnostic test [mRDT] that was introduced in the 2014 version) [7]. Adherence to the IMCI guidelines is low across geographical settings [9–11]. Electronic IMCI versions (e-IMCI) provide a more user-friendly format and may thereby increase algorithm adherence and the consistency of clinical assessments [2,12]. Beyond e-IMCI, electronic algorithms also have the potential to integrate more complex information while maintaining a simple user interface.

In addition to facing format-related barriers, IMCI implementation also faces content-related challenges. The algorithm lacks guidance for a significant proportion of febrile children, such as children without localizing symptoms (fever without source [FWS]). For such children, IMCI instructs clinicians to “give appropriate antibiotic treatment for an identified bacterial cause of fever” but provides no guidance on how to identify a bacterial cause of fever [7]. Before introduction of mRDT-based treatment, IMCI presumptively classified all children with fever as having malaria. Now that mRDTs are used, and lacking adequate diagnostics for bacterial infections, clinicians have practically exchanged antimalarial against nonselective antibiotic treatment for patients with negative mRDTs [13]. This has led to a tremendous overuse of antibiotics at the peripheral level [4]. To address this challenge of diagnosing bacterial infections in children with FWS, a revised IMCI-based algorithm was developed (ALMANACH; S2 Fig). It includes urine dipstick testing and a clinical predictor or rapid test for typhoid [14]. However, these 2 diseases represent only a fraction of bacterial infections that need to be considered [15]. A second content-related challenge is that the IMCI algorithm relies on clinical symptoms alone, which inherently lack diagnostic accuracy in identifying children in need of antibiotic treatment or referral for hospital-based supportive care [16]. Host biomarkers that can help identify children with bacterial infections, such as C-reactive protein (CRP) and procalcitonin (PCT), have not been considered within the IMCI strategy [17]. The safety of using CRP or PCT cutoffs to decide on antibiotic prescription in children has never been evaluated. Besides 1 trial in Vietnam that evaluated using CRP without provision of clinical guidance to guide antibiotic prescription in mild respiratory infections [18], all studies of CRP and PCT in children have focused on analytical performance; none have assessed whether using these tests would change patient outcome. Furthermore, some point-of-care tests (POCTs) may help detect disease in children with severe symptoms where clinical signs lack diagnostic accuracy: for example hemoglobin (Hb) testing can identify children with severe anemia in need of blood transfusions. Lastly, the diagnostic value of the clinical signs that were included in IMCI (based on expert opinion and small derivation studies) has changed. The epidemiology context of infections has indeed shifted away from bacterial and parasitic infections towards viral infections [15]. Additionally, since development of IMCI 30 years ago, a considerable amount of novel evidence on such clinical signs has emerged, which has not been integrated into IMCI thus far [16].

Based on the challenges and opportunities identified, we constructed a novel electronic patient management algorithm, e-POCT, on the IMCI backbone. e-POCT is derived from the latest evidence of pediatric fever management based on studies from both low- and high-income settings.

e-POCT is built into an Android application, which guides the clinician through the entire consultation and recommends management based on a few clinical elements as well as POCTs. The POCTs used are aimed at triaging children with severe disease who require referral to a higher level of care (oxygen saturation [SaO2], heart rate, blood glucose, and Hb), detecting malaria infection (mRDT), and distinguishing between bacterial and viral diseases (CRP and PCT). Given the innovative approach of the algorithm, we first sought to assess its safety when applied to children presenting with febrile illnesses in a low-resource setting. Hence, the objectives of this study were to determine whether e-POCT was non-inferior in terms of clinical outcome to a validated electronic algorithm derived from IMCI (ALMANACH) when managing febrile illness in children under 5 years and to compare the proportion of antibiotic prescriptions and severe adverse events (deaths and secondary hospitalizations) between the 2 arms.

## Methods

### Ethics

The study protocol and related documents were approved by the institutional review boards of the Ifakara Health Institute and the National Institute for Medical Research in Tanzania, by the Ethikkommission Beider Basel in Switzerland, and the Boston Children’s Hospital ethical review board. An independent data and safety monitoring board oversaw the study. The trial was registered in ClinicalTrials.gov, identifier NCT02225769.

### Study design

This was a randomized (at patient level), open controlled trial to investigate whether a novel electronic algorithm using point-of-care (POC) testing, e-POCT, was not inferior in terms of clinical outcome to a validated electronic algorithm derived from IMCI (ALMANACH) when treating febrile infections in children under 5 years of age. In parallel to the randomized study, a small cohort of children managed per routine care was observed, and their clinical outcomes were compared to those of the children treated using e-POCT. The protocol for this trial, the statistical analysis plan, and supporting CONSORT checklist are available as supporting information (S1 and S2 Texts; S1 Table).

### Participants

This study was conducted in the city of Dar es Salaam, Tanzania. Malaria endemicity in this region is relatively low, with about 10% of fever patients positive for malaria; transmission is perennial with a peak in the post-rainy season [15]. Consecutive patients presenting for acute care during normal business hours at the outpatient departments of 3 district hospitals and at 6 health centers in Dar es Salaam were screened for eligibility. Recruitment sites were chosen to represent the pediatric outpatient population in Dar es Salaam. Inclusion criteria were age 2 to 59 months, history of fever for 7 days or less, and axillary temperature ≥ 37.5°C at presentation. Exclusion criteria were weight less than 2.5 kg, main complaint being an injury or acute poisoning, or previous medical care for the present illness. Children satisfying the inclusion and exclusion criteria were enrolled if the parent or guardian had received full information on the study and signed written informed consent.

### Randomization

For the main comparison between e-POCT and ALMANACH, patients were enrolled by the study clinicians and then randomized to 1 of the 2 management arms. They were individually randomized in blocks of 4 according to a computer-generated randomization list provided by an independent, off-site researcher. Sealed, opaque forms were used for allocation concealment and were opened only after the patient’s enrollment. Patients in the routine care cohort were enrolled by a research assistant and directed to a routine clinician at the corresponding health center.

### Interventions and study procedures

**Fig 1:**
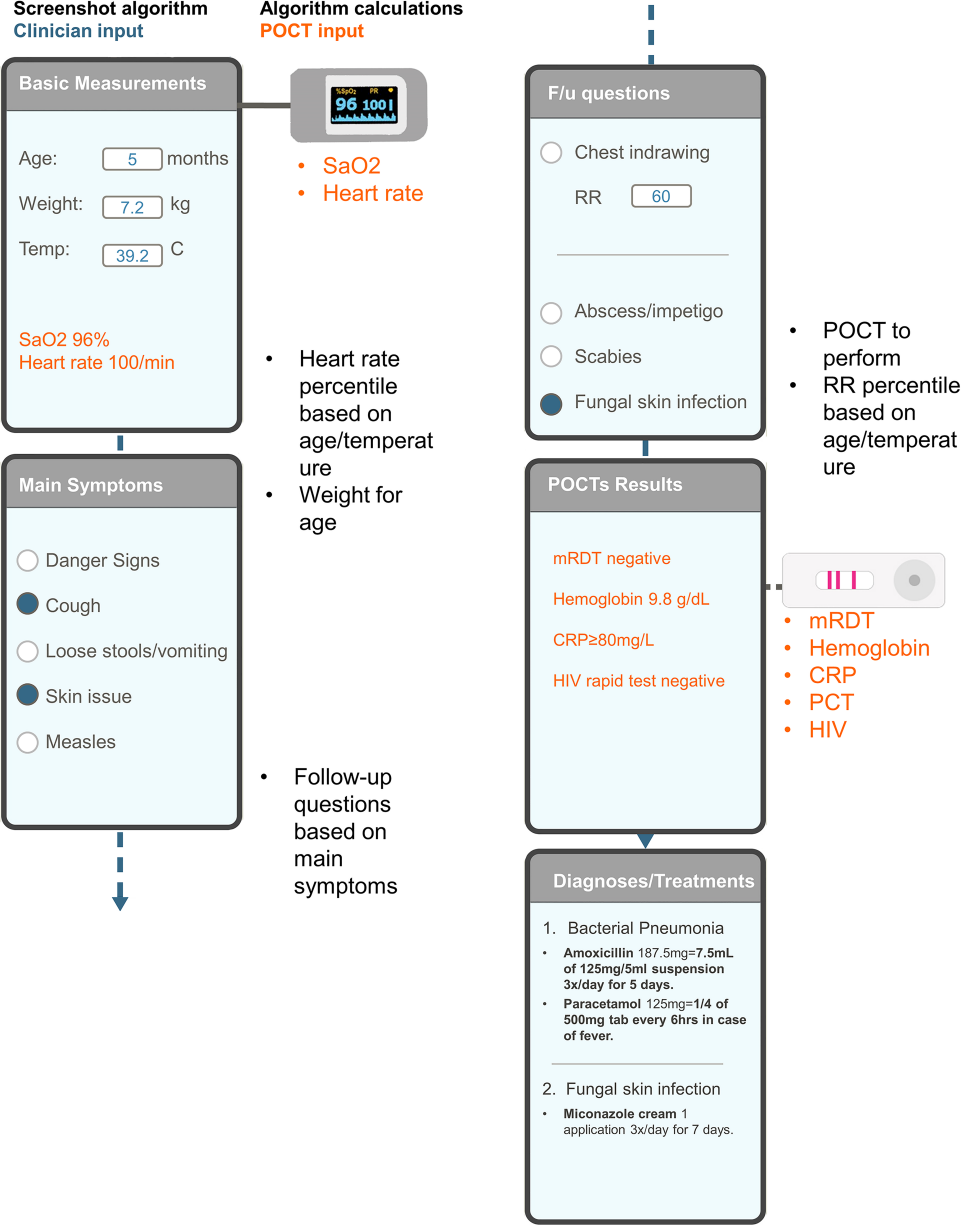
Schematic representation of the e-POCT algorithm. Example of input and output screens, sensor input, and background algorithm calculations. An example of a consultation with respective input and output screens is shown on the left; the background calculations of the algorithm are displayed on the right in black; input of information from POCTs is displayed in orange. CRP, C-reactive protein; F/u, follow-up; HIV, human immunodeficiency virus; PCT, procalcitonin; POCT, point-of-care test; mRDT, malaria rapid diagnostic test; RR, respiratory rate; SaO2, oxygen saturation.

**Fig 2:**
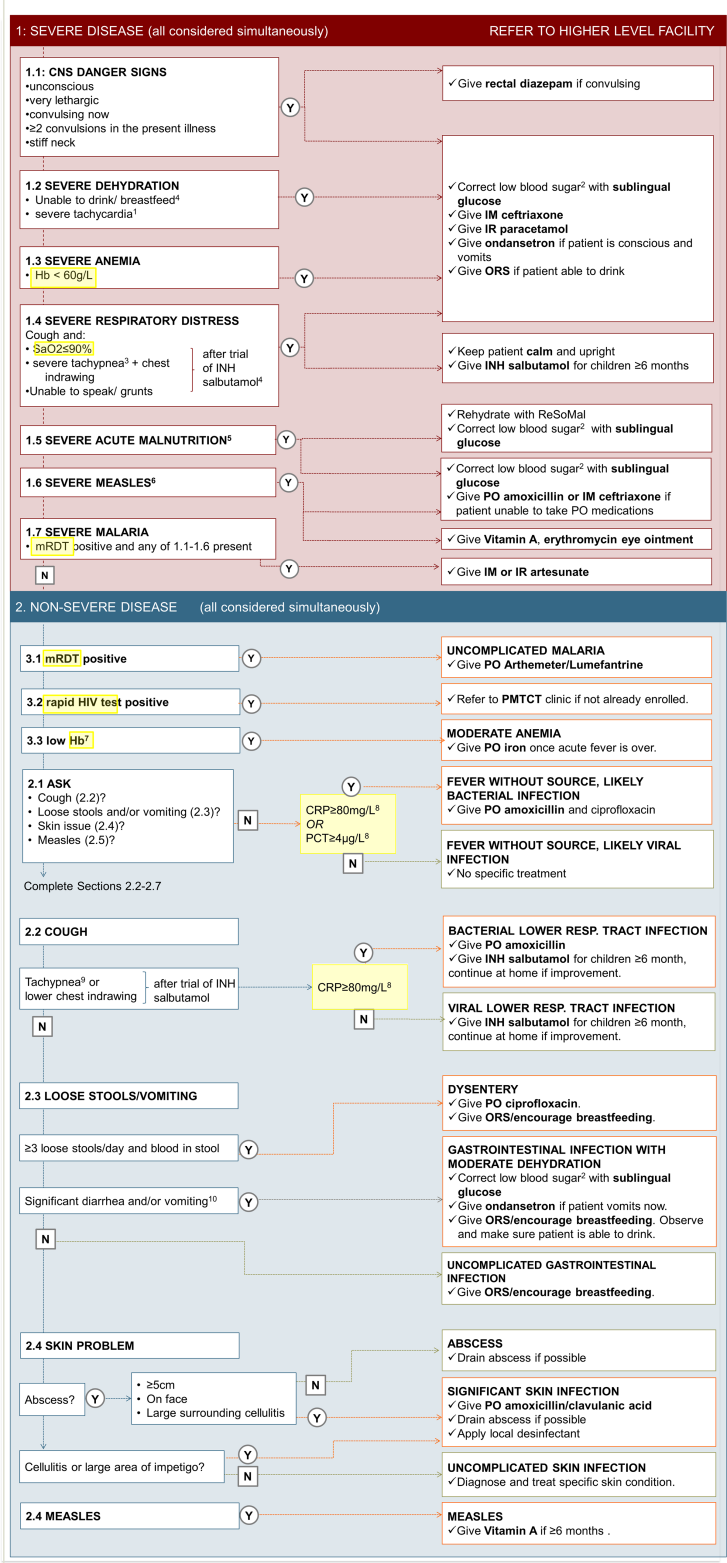
Content of the e-POCT algorithm. The main content (algorithm) of e-POCT is displayed. Questions and information requested by the algorithm are shown on the left side, the respective disease classifications and treatment recommendations on the right. ^1^Heart rate ≥ 90th percentile for age and temperature [19]. ^2^Blood glucose < 3.3 mmol/l. ^3^Respiratory rate ≥ 97th percentile for age and temperature [20]. ^4^Children ≥ 6 months only. ^5^Weight-for-age *z*-score < 3, per WHO 2006 growth charts, and/or mid-upper arm circumference < 11.5 cm and age > 6 months. ^6^Clouding of cornea or severe mouth ulcers or cough and tachypnea (respiratory rate ≥ 75th percentile for age and temperature [20]). ^7^Hb < 90 g/l (2–6 months), < 100 g/l (7–24 months), < 110 g/l (25–59 months). ^8^Measured for patients with negative mRDT only. ^9^Respiratory rate ≥ 75th percentile for age and temperature [20]. ^10^>5 loose stools over past 24 hours or ≥3 loose stools over past 24 hours and emesis or >3 emeses over past 24 hours. CNS, central nervous system; Hb, hemoglobin; IM, intramuscular; INH, inhaled; IR, intrarectal; mRDT, malaria rapid diagnostic test; ORS, oral rehydration solution; PMTCT, prevention of mother-to-child transmission; PO, per os; resp., respiratory; SaO2, oxygen saturation.

The intervention consisted in having study clinicians use the e-POCT algorithm (e-POCT arm) or ALMANACH algorithm (control arm) during the consultation to manage the patient. The development, rationale, and content of the e-POCT algorithm are detailed in S1 Text. In brief, we performed a structured literature review focusing on (i) the identification of children with severe infections requiring referral, (ii) the identification of children with serious bacterial infections, including using CRP and PCT to predict the need for antibiotic treatment, and (iii) the identification of children with dehydration (S2 Table; S1 Fig). The evidence retrieved is described in detail in S3 Text. It was used to design the novel e-POCT electronic algorithm (Figs 1 and 2).

e-POCT differs from the 2014 version of IMCI in the following ways: (i) use of pulse oximetry to identify children with hypoxemia and severe tachycardia, (ii) use of Hb testing to detect children with severe anemia, (iii) construction of a “severe respiratory distress” classification, (iv) refinement of criteria for severe malnutrition, (v) use of a 2-step approach including temperature- and age-corrected respiratory rate and CRP for diagnosing bacterial pneumonia, and (vi) use of CRP and PCT to decide on antibiotic prescription for children with fever without localizing symptoms. The main differences between the e-POCT and ALMANACH algorithms, as well as IMCI, are summarized in Table 1. We constructed a novel electronic algorithm, e-POCT, that was programmed into an Android-based mobile tool. The electronic version allowed integrating a greater amount of data, more elaborate calculations, and direct connection to the oximeter, without increasing the complexity of the consultation process for the clinician.

**Table 1:** Summary of the main differences between e-POCT, ALMANACH, and the 2014 version of IMCI.

Disease category	Classification	e-POCT			ALMANACH [14]			IMCI (2014) [7]		
		Clinical signs/symptoms	Vital sign	POCT	Clinical signs/symptoms	Vital sign	POCT	Clinical signs/symptoms	Vital sign	POCT
**Severe disease**	CNS infection	- Severe lethargy - Stiff neck - ≥2 convulsions			- Severe lethargy, - Stiff neck - ≥1 convulsions			- Severe lethargy, - Stiff neck - ≥1 convulsions		
	Severe pneumonia	Severe respiratory distress^1^	Severe tachypnea^2^	SaO2 < 90%	- Chest indrawing - Cyanosis - Stridor			- Stridor - Chest indrawing and		HIV positive
	Severe anemia			Hb < 60 g/l	Severe palmar pallor			Severe palmar pallor		
	Severe dehydration	Not tolerating oral liquids		Severe tachycardia^3^	Vomits everything or 2 of: lethargic/unconscious; sunken eyes; not able to drink/drinks poorly; skin pinch very slow			Vomits everything or 2 of: lethargic/unconscious; sunken eyes; not able to drink/drinks poorly; skin pinch very slow		
	Severe malnutrition		Very low WFA and/or MUAC^4^		- Severe wasting - Edema of both feet			- Edema of both feet or - Very low WFH/MUAC combined with complications^5^		
	Other severe disease	None			- Jaundice - Tender swelling behind ear - Infected skin lesion or lump larger than 4 cm or with red streaks or with tender nodes or multiple abscesses			Tender swelling behind ear		
	Severe malaria	Any severe classification and		Positive mRDT	Not considered			Not considered		
**Non-severe disease**	Clinical pneumonia	Not considered			Cough and	Very fast breathing^6^		Cough and	Fast breathing^7^ Chest indrawing	
	Bacterial LRTI	Cough and	Tachypnea^8^ and	CRP ≥ 80 mg/l	Not considered			Not considered		
	Viral LRTI/bronchiolitis	Cough and	Tachypnea^8^ and	CRP < 80 mg/l	Not considered			Not considered		
	Upper respiratory infection	Cough and	No tachypnea^8^		Cough and	No very fast breathing^6^		Cough and	No fast breathing^7^	
	Gastrointestinal infection with dehydration	Significant diarrhea and/or vomiting^9^			Two of: restless/irritable; sunken eyes; drinks eagerly/thirsty; skin pinch slow			Two of: restless/irritable; sunken eyes; drinks eagerly/thirsty; skin pinch slow		
	Skin infection	Skin infection without additional severe diagnosis^10^			Infected skin lesion smaller than 4 cm and without red streaks and without tender nodes or single abscesses			Not considered in main algorithm		
	Fever without source, likely bacterial infection			CRP ≥ 80 mg/l and/or PCT ≥ 4 μg/l	Purulent ear discharge		Positive urine dipstick (<2 years only) or positive typhoid test (≥2 years only)	Not considered		
	Fever without source, likely viral infection			CRP < 80 mg/l and PCT < 4 μg/l			Negative urine dipstick (<2 years only) and negative typhoid test (≥2 years only)	Not considered		
	Uncomplicated malaria			Positive mRDT			Positive mRDT			Positive mRDT

The conjunctions “and” or “or” are to be read across columns.

^1^Severe respiratory distress: Speaks only single words or grunts or speaks short phrases only or short cries and lower chest wall indrawing.

^2^Severe tachypnea: respiratory rate ≥ 97th percentile for age and temperature [20].

^3^Severe tachycardia: heart rate ≥ 90th percentile for age and temperature [19].

^4^WFA *z*-score < −3 and/or MUAC < 11.5 cm and age > 6 months.

^5^WFH *z*-score < −3 and/or MUAC < 11.5 cm and age > 6 months; complications defined as feeding problem or medical problem. Please note that ALMANACH was developed based on the 2008 version of IMCI, which used only clinical signs for the identification of malnutrition.

^6^Very fast breathing: respiratory rate ≥ 50/min, regardless of age.

^7^Fast breathing: respiratory rate ≥ 50/min and age < 12 months or RR ≥ 40/min and age ≥ 12 months.

^8^Tachypnea: respiratory rate ≥ 75th percentile for age and temperature.

^9^>5 loose stools over past 24 hours or ≥3 loose stools over past 24 hours and emesis or >3 emeses over past 24 hours.

^10^Algorithm provides diagnoses and specific treatment recommendations for 13 common skin diseases (abscess, cellulitis, impetigo/pyoderma, tinea corporis, pityriasis versicolor, candidiasis, tinea capitis, scabies, chicken pox, herpes, larva migrans, eczema, urticarial).

CNS, central nervous system; CRP, C-reactive protein; IMCI, Integrated Management of Childhood Illness; LRTI, lower respiratory tract infection; mRDT, rapid test for malaria; MUAC, mid-upper arm circumference; PCT, procalcitonin; POCT, point-of-care test, SaO2, oxygen saturation, HIV, human immunodeficiency virus; WFA, weight-for-age; WFH, weight-for-length/height.

Children enrolled in the intervention arm were assigned to study clinicians using e-POCT (Figs 1, 2 and S1) during 2 weeks, while children enrolled in the control arm were managed by other study clinicians using ALMANACH (S2 Fig). In order to minimize a cluster effect at clinician level, clinicians then switched arms, and thus algorithms, every 2 weeks. Based on their assignment, enrolled children were directed either to the e-POCT or ALMANACH clinician.

Compared to the IMCI-based algorithm (ALMANACH) used by study clinicians in the control arm, e-POCT uses fewer clinical symptoms and signs. Rather, it relies on signs that can be measured objectively: hypoxemia and severe tachycardia (oximeter), severe anemia (POC hemoglobinometer), hypoglycemia (POC glucometer), as well as host biomarkers of inflammation predictive of bacterial infection (elevated CRP using a rapid semi-quantitative lateral-flow test and elevated PCT using a POC immunoassay system).

We chose ALMANACH, instead of the paper IMCI, as a control group, since ALMANACH is also built into an Android support tool: we were interested in comparing the impact related to the content of the algorithms rather than the format and technological features. In addition, since our goal was to reduce antibiotic prescription while ensuring optimal clinical patient outcome, and since a reduction in both antibiotic prescriptions and clinical failures using ALMANACH versus routine care has already been demonstrated, we regarded ALMANACH as the current gold standard in terms of antibiotic prescription and used it for the reference control arm [2]. In order to monitor routine care practice during the study, children were included in a routine care cohort in 2 participating health centers. After enrollment, patients were managed by routine clinicians. There was no intervention done, but we assured that essential laboratory tests and medicines were available at the health center.

Rapid diagnostic testing for malaria was done for all patients (including in the routine care cohort) using either the SD BIOLINE Malaria Ag P.f/Pan (Standard Diagnostics) or CareStart Malaria HRP2 (Access Bio) assay. Other POCTs were performed on site as recommended by the algorithms (Figs 1, 2 and S2; Table 1). Following Tanzanian national guidelines, voluntary screening for human immunodeficiency virus (HIV) antibodies using the Determine HIV-1/2 (Alere) was offered to all patients when HIV test kits were available at the health facilities. In the routine care cohort, voluntary screening was offered at the routine clinician’s discretion per standard practice. In the e-POCT arm, Hb measurement (HemoCue 201+ photometer) and oximetry (NONIN XPod with pediatric probe) were done in all patients. Children with clinical signs of lower respiratory tract infection (LRTI) (Table 1) underwent CRP testing to decide on antibiotic prescription for pneumonia. For children with FWS, the e-POCT algorithm uses combined CRP and PCT testing (Table 1). For CRP testing, we used a POC semi-quantitative assay (bioNexia CRPplus, Biomérieux). PCT values were determined on site using the B.R.A.H.M.S PCT assay on the miniVIDAS platform (Biomérieux, Thermo Scientific). Using ALMANACH, children less than 2 years with FWS underwent urine dipstick testing, as well as older children with dysuria (Table 1). Children 2 years or older with FWS were tested for typhoid using the Typhidot assay (Reszon Diagnostics International).

### Follow-up

All caregivers were asked to return with the child for scheduled visits on days 3 and 7, or at any time if the parent was concerned about the child’s condition. Patients cured at day 3 were followed up by phone only on day 7. Field workers traced patients missing the day 7 follow-up. For admitted patients, the scheduled visits were done in the hospital. Patients not cured (see definition below under “Outcomes”) before day 7 were treated again per the assigned algorithm, i.e., the e-POCT algorithm if they were part of the e-POCT arm or ALMANACH if in the control arm. Patients not cured before day 7 in the routine care cohort were treated at the routine clinician’s discretion. Patients not cured at day 7 were treated per the study clinician’s judgment, and another follow-up visit was performed at day 14 to assure that the child was cured. All patients were called by phone at day 30 to assess for severe adverse events (secondary outcome measure, see below). When the algorithm recommended referral (or a routine clinician decided to refer a patient), a field worker escorted the patient to the nearest referral hospital. Patients were then admitted (or discharged home) and managed at the discretion of the responsible medical doctor in the referral hospital.

### Outcomes

The primary outcome measure was the risk of clinical failure (Table 2) by day 7. At follow-up, clinicians recorded the variables that were used to calculate the criteria for clinical failure per Table 2 (the variables were either already part of the electronic algorithm assessment or otherwise recorded on paper forms). However, the clinicians were unaware of the study criteria for clinical failure and how the variables recorded were used to calculate study outcomes. The study outcomes were not used to decide on patient management. To guarantee equal assessments of the primary outcome in both arms, the following additional definition was applied: Patients were considered “not cured” and were treated again using the respective algorithm (or per the routine clinician) if either (i) the caregiver considered that the child was still ill or (ii) the child still had fever when assessed by trained field workers who did not know the content of the algorithms nor the criteria for clinical failure. The secondary outcome measures were the proportion of antibiotic prescriptions at day 0 and between day 1 and day 6, the proportion of primary referrals at day 0, and the proportion of severe adverse events (secondary hospitalizations and death) by day 30.

**Table 2:** Definition of clinical failure by day 7 (primary outcome measure).

At any time between initial assessment and day 7	At day 3	At day 7
• Severe disease: - Coma - More than 2 convulsions within 24 hours - Inability to drink or breastfeed - Hypoxemia (SaO2 < 90%) - Severe tachypnea^1^ - Severe tachycardia^2^	• Clinical pneumonia: - History of cough and tachypnea^3^ - History of cough and lower chest indrawing • Significant dehydration^4^	• Fever or temperature ≥ 38°C • Clinical pneumonia: - History of cough and tachypnea^3^ - History of cough and lower chest indrawing • Diarrhea^5^ • Significant dehydration^4^ • Serious skin infection^6^ • A new significant symptom or sign related to the acute episode but not present at day 0

^1^Respiratory rate ≥ 97th percentile for age and temperature [20].

^2^Heart rate ≥ 90th percentile for age and temperature [19].

^3^Respiratory rate ≥ 60/min and age < 12 months or respiratory rate ≥ 50/min and age ≥ 12 months.

^4^Dehydration requiring facility-based treatment.

^5^≥3 liquid stools per day.

^6^Skin infection requiring systemic antibiotic treatment and/or facility-based treatment.

SaO2, oxygen saturation.

### Sample size and statistical analyses

The sample size was computed for the primary analysis based on a 97.5% (1-sided) confidence interval (CI). To prove non-inferiority, the upper limit of this CI was to be within 3%. This non-inferiority margin was chosen because 3% was considered a clinically meaningful difference in clinical failure by day 7. The proportion of clinical failures by day 7 was estimated to be 10% in both arms based on prior studies using ALMANACH in the same area [2]. Assuming 80% statistical power, 3,140 patients were needed to show whether the difference in clinical failure by day 7 between the e-POCT and ALMANACH arms was within 3%.

**Fig 3:**
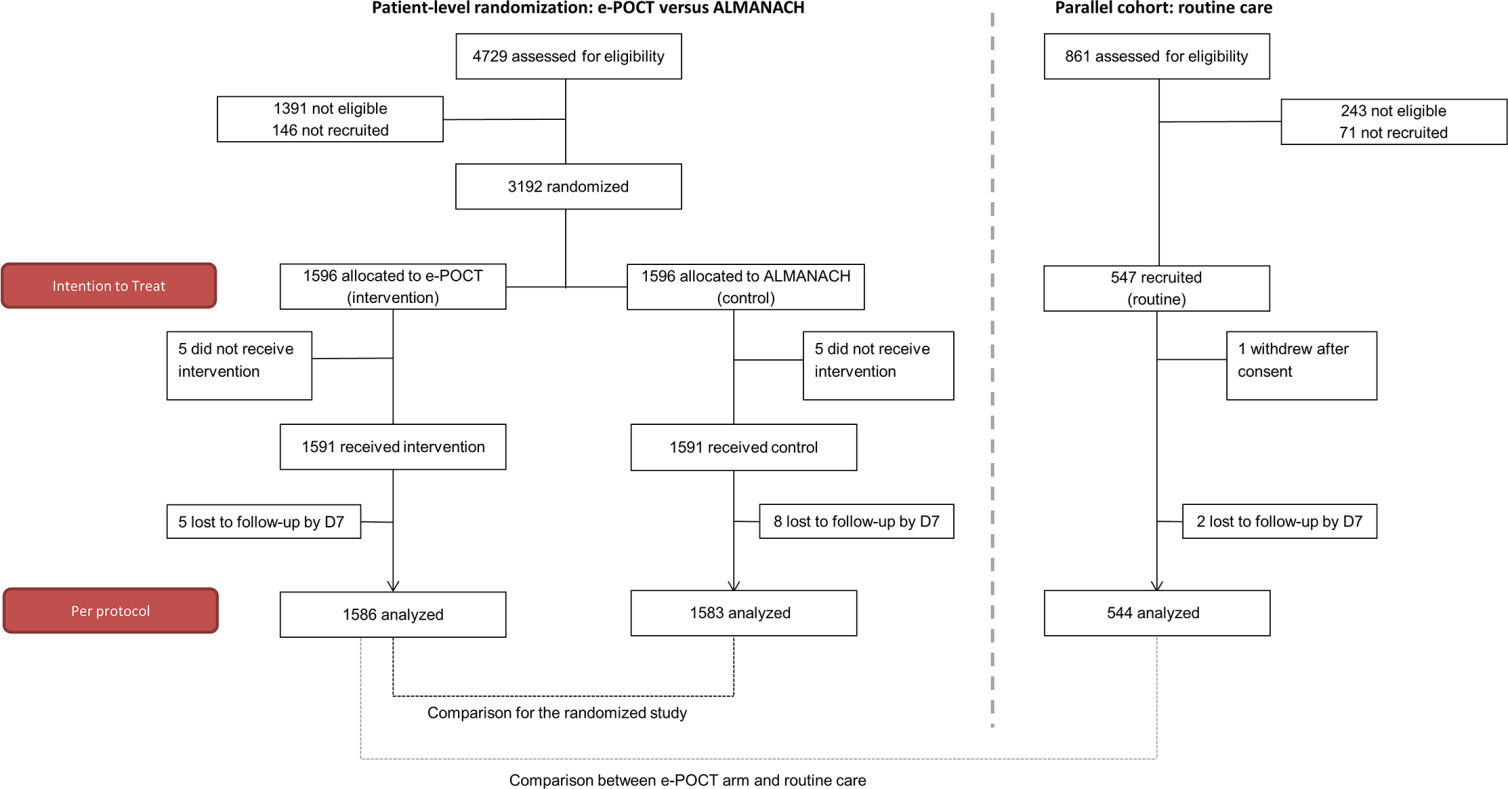
Patient flowchart. “Not eligible” refers to patients who did not meet inclusion criteria or met exclusion criteria.

Interim analyses of clinical failure rates were performed after inclusion of the first 200 and 1,000 patients. A stopping rule was predefined for an absolute difference in clinical failure by day 7 of more than 5% between e-POCT and ALMANACH. Both intention-to-treat (ITT) and per-protocol (PP) study populations were defined. The ITT population comprised all randomized patients (or patients recruited into the routine care cohort); per definition, patients who were lost to follow-up were treated as clinical failures. The PP population included all randomized patients (or patients recruited into the routine care cohort) who received the intervention (or were attended by the routine clinician) and completed the day 7 assessment (Fig 3).

Since this was a non-inferiority trial, and bias towards the null would tend to favor non-inferiority, we used a PP analysis as our primary analysis. Accordingly, all results are displayed according to PP analyses if not stated otherwise. Risk difference (RD) and risk ratio (RR) values with 95% CIs were calculated to estimate the intervention effects on the main study outcomes using the Stata cs procedure; associations between the interventions and outcomes were checked using the chi-squared test. Stratified analyses with Mantel–Haenszel estimates for RR were performed to explore the statistical heterogeneity of effect between health centers and clinicians [21]. For the primary outcome, mixed effects logistic regression was used to adjust for possible confounding covariates. Health center was modeled as a random effect, and clinician as a fixed effect. Additional predictors (age in months, weight-for-age *z*-score, body temperature, respiratory rate, heart rate, past medical history, and maternal education) were chosen based on clinical reasoning and were introduced into the model in a stepwise forward selection process. Predictors that were either a confounder or significantly related to the outcome were kept in the final model. Changes in odds ratio (OR) were used as approximated changes in RR since the primary outcome was rare. Kaplan–Meier survival analysis was used to compare the duration of fever between the 2 study arms.

## Results

### Recruitment and follow-up

Overall, between December 2014 to February 2016, 4,729 patients were screened, and 3,192 randomized (ITT population; Fig 3) into the main trial. Eight hundred fifty-eight (27%) patients were recruited in the rainy season, 736 (23%) in the post-rainy season, and the remaining 1,598 (50%) in the dry season. The PP population consisted of 3,169 patients (1,586 in the e-POCT arm and 1,583 in the control arm); 4 patients in the e-POCT arm and 5 in the control arm withdrew consent after randomization. There was only 1 algorithm deviation in the e-POCT arm and none in the ALMANACH arm. In all, 5 and 8 patients were lost to follow-up for the day 7 outcome assessment in the e-POCT and control arm, respectively. For the routine care cohort, 547 patients were recruited into the cohort between January and December 2015 (ITT population; Fig 3); 1 refused participation after consent, and 2 were lost to follow-up for the day 7 outcome assessment, leaving 544 for the PP analysis (Fig 3).

The day 30 phone follow-up could not be completed in 20/1,586 patients (1.3%) in the e-POCT arm, 25/1,583 patients (1.6%) in the control arm, and 6/544 patients (1.1%) in the routine care cohort. For the e-POCT and ALMANACH arms as well as the routine cohort, follow-up intervals for the day 3 and day 7 outcome assessments were 3 (IQR 3–3, range 2–5) and 7 (IQR 7–7, range 6–12) days, respectively. The distribution of follow-up intervals did not differ between the e-POCT and ALMANACH arms (Wilcoxon rank-sum *p =* 0.54 for day 3, *p =* 0.96 for day 7), nor between the e-POCT arm and the routine care cohort (Wilcoxon rank-sum *p =* 0.60 for day 3, *p =* 07 for day 7). Baseline characteristics did not differ between the e-POCT and ALMANACH arms (Table 3). Median age of children enrolled into the trial was 13 months (IQR 9–22); 54% (2,008/3,713) of the children were male (Table 3). In the routine care cohort, fewer patients were reported to have IMCI danger signs by the routine clinician than by clinicians in the other 2 arms (Table 3).

**Table 3:** Baseline characteristics.

Characteristic	Randomized study			Routine care cohort	
	Total *N*	e-POCT arm	ALMANACH arm	*N*	Value
***Demographic***					
**Male sex**	3,192	891/1,596 (56)	875/1,596 (55)	547	259 (47)
**Age group**	3,192			547	
2–11 months		686/1,596 (43)	721/1,596 (45)		225 (41)
12–23 months		553/1,596 (35)	501/1,596 (32)		206 (38)
≥24 months		357/1,596 (22)	374/1,596 (23)		116 (21)
**Primary caregiver other than mother**	3,113	64/1,555 (4)	66/1,558 (4)	514	8 (2)
**Mother’s highest grade of education**	3,106			514	
None		137/1,551 (9)	152/1,555 (10)		30 (6)
Primary		1,060/1,551 (68)	1,015/1,555 (65)		348 (68)
Post-primary		354/1,551 (23)	388/1,555 (25)		136 (27)
**Number of children in household, median (IQR)**	3,100	2 (1–3)	2 (1–3)		2 (1–2)
***Medical history***					
**Main reason for consultation**	3,192			547	
Fever only		214/1,596 (13)	216/1,596 (14)		54 (10)
Cough		917/1,596 (57)	890/1,596 (56)		323 (59)
Rhinorrhea/nasal congestion		669/1,596 (42)	696/1,596 (44)		159 (29)
Diarrhea		313/1,596 (20)	328/1,596 (21)		107 (20)
Vomiting		312/1,596 (20)	286/1,596 (18)		63 (12)
**Duration of fever**	3,180			543	
1 day or less		1,004/1,589 (63)	982/1,591 (62)		363 (67)
2–4 days		569/1,589 (36)	591/1,591 (37)		174 (32)
5 days or more		16/1,589 (1)	18/1,591 (1)		6 (1)
**Duration of cough**	1,827			331	
2 days or less		629/923 (68)	606/904 (67)		252 (76)
3–6 days		282/923 (31)	292/904 (32)		252 (22)
7 days or more		12/923 (1)	6/904 (1)		252 (2)
***Clinical signs***					
WFA *z*-score, mean (SD)	3,189	−0.8 (1.3)	−0.7 (1.3)	547	
Severe malnutrition^1^	3,189	56/1,595 (4)	50/1,594 (3)	547	15 (3)
Respiratory rate, median (IQR)	3,180	41 (36–49)	41 (36–51)	528	43 (36–49)
Heart rate, mean (SD)	3,142	145 (17)	143 (16)	—	—^4^
Tachypnea per IMCI^2^	3,180	419/1,593 (26)	416/1,587 (26)	540	147 (27)
Severe symptom per IMCI^3^	3,192	22/1,596 (1)	23/1,596 (1)	547	2 (0)

Values are *n/N* (percent) unless otherwise indicated.

^1^Weight-for-age (WFA) *z*-score < −3 or mid-upper arm circumference < 11.5 cm and age > 6 months, per WHO 2006 growth curve [22].

^2^Age <12 months and respiratory rate ≥ 50/min, or age ≥12 months and respiratory rate ≥ 40/min.

^3^Positive meningeal signs, convulsion or history of convulsion, lethargy, severe anemia, HIV positive with chest indrawing, or severe malnutrition with complications.

^4^Heart rate was not measured in the routine care cohort.

IMCI, Integrated Management of Childhood Illness.

### Clinical failure and severe adverse events in the randomized study

**Fig 4:**
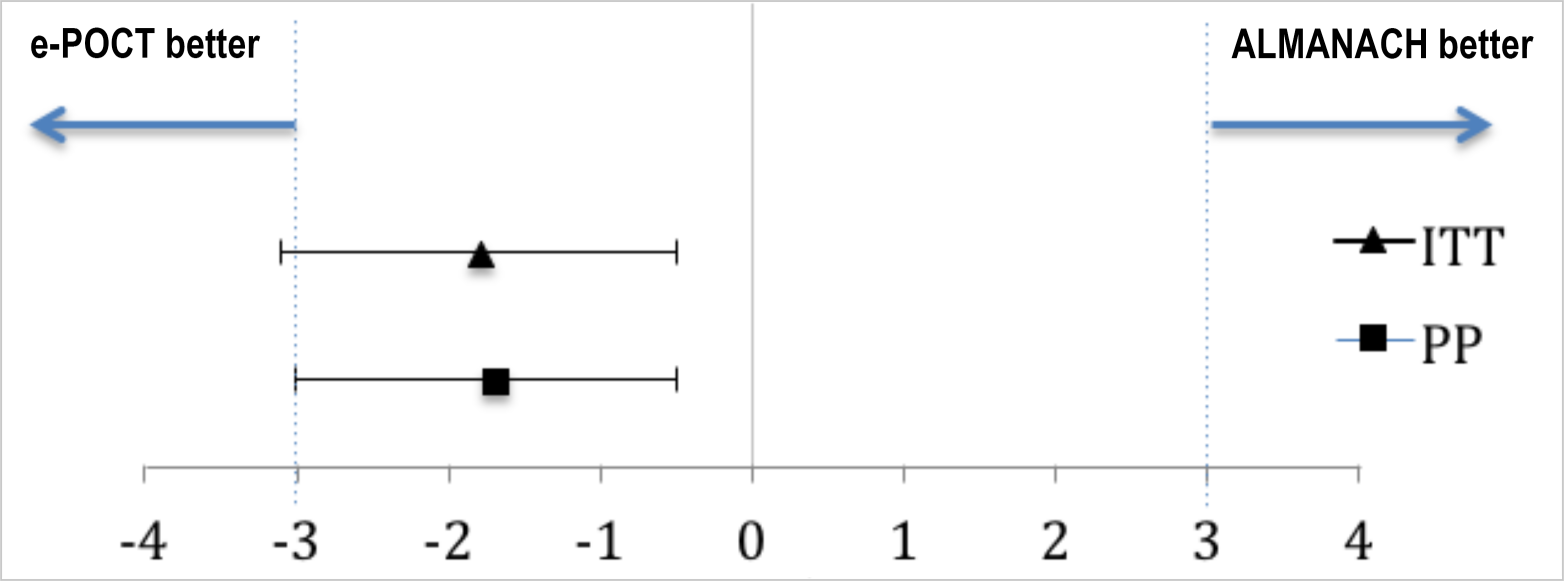
Non-inferiority plot comparing clinical outcome at day 7 in the e-POCT and control arm of the randomized study. The point estimates of the risk difference in clinical failure by day 7 and their respective 95% confidence intervals are displayed in black. The dotted blues line show the predefined inferiority margin of 3%. In none of the performed analyses was the non-inferiority margin exceeded. ITT, intention-to-treat; PP, per-protocol.

Table 4 shows the primary and secondary study outcomes for the randomized study (PP analysis). The results of the ITT analysis are summarized in S3 Table. In the PP population, 2.3% (37/1,586) of patients experienced clinical failure by day 7 in the e-POCT arm versus 4.1% (65/1,583) of patients in the ALMANACH arm (RD −1.7, 95% CI −3.0, −0.5; RR 0.57, 95% CI 0.38, 0.85). There was a 43% reduction in relative risk for clinical failure in the e-POCT arm compared to the ALMANACH control arm. The non-inferiority plot of clinical failure in the ITT and PP populations is displayed in Fig 4.

**Table 4:** Primary and secondary study outcomes of the randomized study (per-protocol population).

Outcome	e-POCT arm, percent (*n/N*)	ALMANACH arm, percent (*n/N*)	Risk difference (95% CI)	Risk ratio (95% CI)	*p*-Value^1^
***Primary outcome***					
**Clinical failure by day 7**	2.3 (37/1,586)	4.1 (65/1,583)	−1.7 (−3.0, −0.5)	0.57 (0.38, 0.85)	0.005
***Secondary outcomes***					
**Primary referrals**	6.6 (104/1,586)	2.9 (46/1,583)	3.6 (2.2, 5.1)	2.26 (1.61, 3.17)	<0.001
**Antibiotic prescription at day 0**	11.5 (182/1,586)	29.7 (470/1,583)	−18.2 (−21.0, −15.5)	0.39 (0.33, 0.45)	<0.001
**Severe adverse events by day 30**	0.6 (10/1,586)	1.5 (24/1,583)	−0.9 (−1.6, −0.2)	0.42 (0.20, 0.87)	0.02
Secondary admissions	0.4 (7/1,586)	1.2 (19/1,583)	−0.8 (−1.4, −0.1)	0.37 (0.15, 0.87)	0.02
Deaths	0.2 (3/1,586)	0.4 (6/1,583)	−0.2 (−0.6, 0.2)	0.50 (0.12, 2.00)	0.32

^1^Chi-squared test.

As depicted in Table 5, the main reduction in clinical failure between the 2 algorithms occurred by day 3 (RR 0.39, 95% CI 0.22, 0.67)—there was no significant difference in clinical failure for days 4–7 (RR 0.93, 95% CI 0.51, 1.72). The crude OR (0.56, 95% CI 0.37, 0.84) for clinical failure by day 7 was similar to the adjusted OR (0.55, 95% CI 0.36, 0.84), adjusted for significant covariates and random effects (age, axillary temperature, weight-for-age *z*-score [22], respiratory rate, clinician, and health center; S4 Table). We did not note statistical heterogeneity between health centers or clinicians for either the primary and the secondary outcome measures (S5 Table). There was a 68% reduction in the relative risk of severe adverse events (secondary hospitalizations and deaths) in the e-POCT arm (0.6%, 10/1,586) compared to the control arm (1.5%, 24/1,583) (RR 0.42, 95% CI 0.20, 0.87). In all, 0.5% (8/1,586) of patients in the e-POCT arm versus 1.4% (22/1,583) of children in the ALMANACH arm developed severe symptoms during follow-up (RR 0.36, 95% CI 0.16, 0.81; Table 5). Within both algorithms, having a severe classification at day 0 was associated with a higher, and having a likely viral infection classification with a lower, risk of clinical failure (Table 6). Within the ALMANACH algorithm, patients with a clinical pneumonia classification also had a higher risk of clinical failure (Table 6).

**Table 5:** Details of clinical failure by day 7 in the randomized study.

Criterion for clinical failure	e-POCT arm			ALMANACH arm		
	Clinical failure	Secondary admission	Death	Clinical failure	Secondary admission	Death
**Day 0–3**						
Coma/convulsion	1	—	—	5	1	5
Hypoxemia/severe tachypnea	1	1	—	7	2	1
Other severe symptom	5	4	1	9	6	—
Cough and tachypnea/chest indrawing	10	2	1	23	3	—
**Total at day 0–3, percent (*n/N*)**	**1.1 (17/1,586)**	**0.4 (7/1,586)**	**0.1 (2/1,586)**	**2.8 (44/1,583)**	**0.8 (12/1,583)**	**0.4 (6/1,583)**
**Day 4–7**						
Hypoxemia/severe tachypnea	1	—	1	—	—	—
Severe anemia	—	—	—	1	1	—
Persistent fever	13	—	—	16	5	—
Cough and tachypnea/chest indrawing	3	—	—	2	—	—
Diarrhea/vomiting	2	—	—	1	—	—
Significant skin infection	1	—	—	1	1	—
**Total at day 4–7, percent (*n/N*)**	**1.3 (20/1,569)**	**0 (0/1,579)**	**0.1 (1/1,584)**	**1.4 (21/1,539)**	**0.4 (7/1,571)**	**0 (0/1,577)**

**Table 6:** Association between clinical failure and disease classification.

Algorithm classification	e-POCT arm		ALMANACH arm	
	Clinical failure by day 7, percent (*n/N*)	RR (95% CI)	Clinical failure by day 7, percent (*n/N*)	RR (95% CI)
Severe disease	5.8 (6/103)	2.79 (1.19, 6.53)	23.6 (13/55)	6.90 (4.03, 11.98)
Clinical pneumonia	—		6.4 (21/330)	1.81 (1.09. 3.00)
Bacterial respiratory infection	0 (0/10)	NA	—	
Viral respiratory infection	3.0 (13/429)	1.46 (0.75, 2.84)	—	
Upper respiratory infection	3.1 (12/391)	1.47 (0.05, 2.89)	3.7 (20/542)	0.85 (0.51, 1.43)
Gastrointestinal disease with dehydration	2.4 (4/169)	1.02 (0.36, 2.83)	4.7 (14/296)	1.19 (0.67, 2.13)
Skin infection	2.7 (2/75)	1.13 (0.28, 4.61)	1.4 (1/69)	0.34 (0.05, 2.44)
FWS, likely bacterial infection	1.9 (1/52)	0.82 (0.11, 5.86)	4.3 (3/70)	1.05 (0.34, 3.25)
FWS, likely viral infection	0.6 (2/349)	0.20 (0.05, 0.84)	1.1 (4/357)	0.23 (0.08, 0.62)
Uncomplicated malaria	0 (0/166)	NA	2.8 (5/180)	0.65 (0.27, 1.61)
Antibiotic treatment recommended (non-severe disease)	1.3 (1/79)	0.59 (0.08, 4.29)	5.8 (24/415)	2.30 (1.35, 3.92)

**Fig 5:**
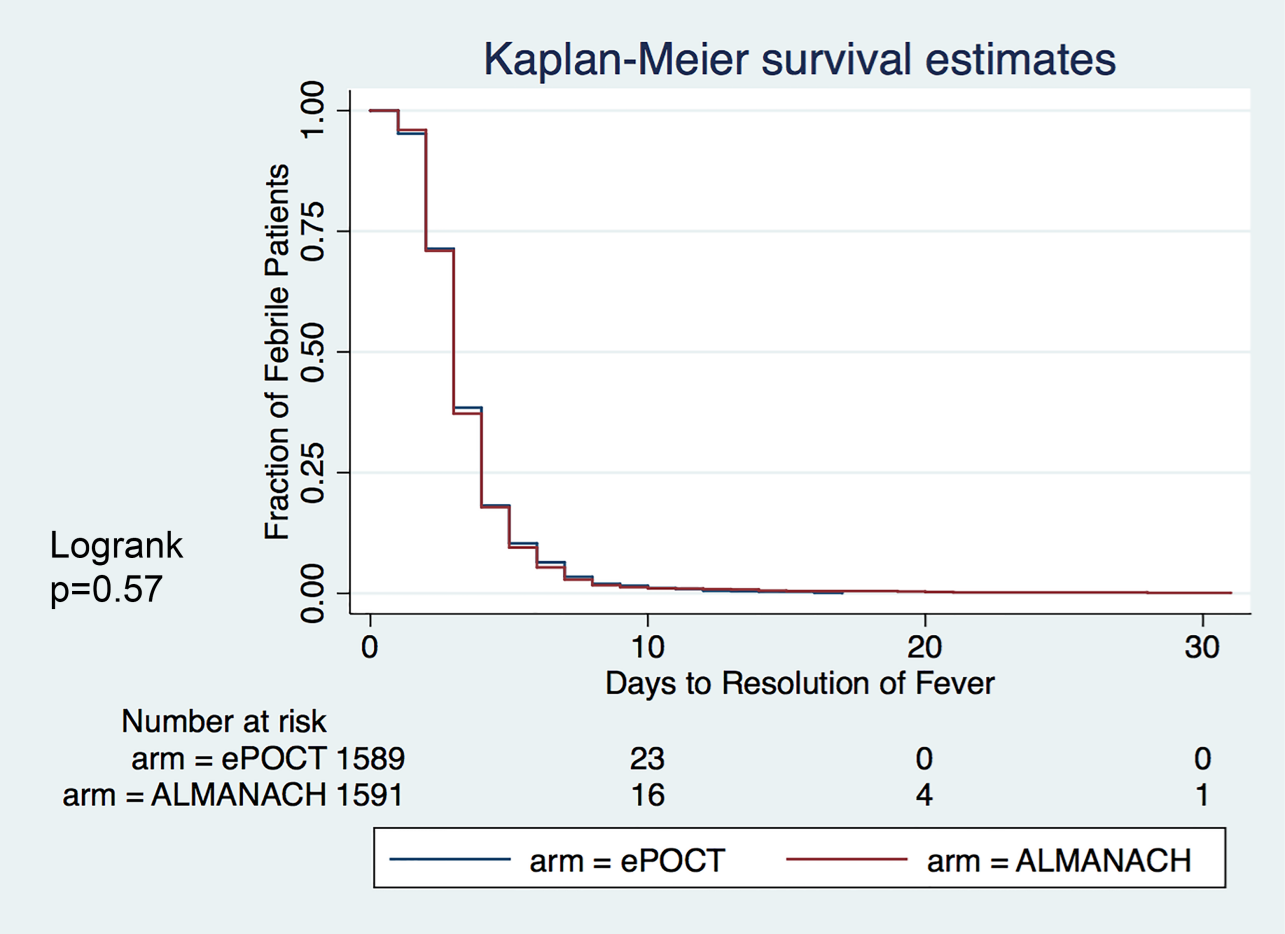
Kaplan–Meier survival estimates: Days to resolution of fever.

Days to resolution of fever was not different between the 2 arms (Fig 5).

FWS, fever without source; NA, not applicable; RR risk ratio.

### Clinical failure and severe adverse events: Comparison of the e-POCT arm with routine care

Table 7 shows the primary and secondary study outcomes for the comparison between the e-POCT arm and the routine care cohort (PP analysis). The results of the ITT analysis are provided in S6 Table. In the PP population, 4.6% (25/544) of patients treated by routine clinicians experienced clinical failure by day 7. There was a 49% lower relative risk of clinical failure in the e-POCT arm compared to routine care (RR 0.51, 95% CI 0.31, 0.84; RD −2.3, 95% CI −4.2, −0.4). Though there were fewer adverse events in the e-POCT arm (0.6%, 10/1,586) compared to routine care (1.1%, 6/544), this difference was not statistically significant (Table 7).

**Table 7:** Primary and secondary study outcomes for comparison between e-POCT and routine care (per-protocol population).

Outcome	e-POCT arm, percent (*n/N*)	Routine care cohort, percent (*n/N*)	Risk difference (95% CI)	Risk ratio (95% CI)	*p*-Value^1^
***Primary outcome***					
**Clinical failure by day 7**	2.3 (37/1,586)	4.6 (25/544)	−2.3 (−4.2, −0.4)	0.51 (0.31, 0.84)	0.007
***Secondary outcomes***					
**Primary referrals**	6.6 (104/1,586)	0.4 (2/544)	6.1 (4.9, 7.5)	17.83 (4.42, 72.02)	<0.001
**Antibiotic prescription at day 0**	11.5 (182/1,586)	94.9 (516/544)	−83.3 (−85.8, −81.0)	0.12 (0.11, 0.14)	<0.001
**Severe adverse events by day 30**	0.6 (10/1,586)	1.1 (6/544)	−0.4 (−1.4, 0.5)	0.57 (0.21, 1.57)	0.27
Secondary admissions	0.4 (7/1,586)	1.1 (6/544)	−0.6 (−1.6, 0.2)	0.40 (0.13, 1.19)	0.09
Deaths	0.2 (3/1,586)	0.2 (1/544)	0.0 (−0.4, 0.4)	1.03 (0.11, 9.87)	0.98

^1^Chi-squared test.

The main reduction in clinical failure between the e-POCT arm and routine care occurred by day 3 (RR 0.34, 95% CI 0.18, 0.67; Tables 5 and 8)—there was no significant difference in clinical failure for days 4–7 (RR 0.84, 95% CI 0.37, 1.90). We found no association between the diagnosis given by the routine clinician on day 0 and clinical failure (Table 9). Time to resolution of fever was not statistically different between the e-POCT arm and the routine care cohort (S3 Fig).

**Table 8:** Details of clinical failure by day 7 in the routine care cohort.

Criterion for clinical failure	Clinical failure	Secondary admission
**Day 0–3**		
Coma/convulsion	1	1
Hypoxemia/severe tachypnea	3	1
Other severe symptom	3	2
Cough and tachypnea/chest indrawing	10	
**Total at day 0–3, percent (*n/N*)**	**3.1 (17/544)**	**0.7 (4/544)**
**Day 4–7**		
Hypoxemia/severe tachypnea	—	
Severe anemia	—	
Persistent fever	4	2
Cough and tachypnea/chest indrawing	2	
Diarrhea/vomiting	1	
Significant skin infection	1	
**Total at day 4–7, percent (*n/N*)**	**1.5 (8/527)**	**0.3 (2/540)**

The 1 death in the routine care cohort occurred on day 20 of follow-up.

**Table 9:** Association between clinical failure and diagnosis given by routine clinicians at day 0.

Routine clinician diagnosis	Clinical failure by day 7, percent (*n/N*)	RR (95% CI)
Severe disease	0 (0/2)	—
Pneumonia	6.5 (2/31)	1.44 (0.36–5.82)
Upper respiratory infection	4.6 (15/325)	1.01 (0.46–2.20)
Gastrointestinal disease	4.7 (5/106)	1.03 (0.40–2.69)
Skin infection	0 (0/23)	—
Urinary tract infection	5.0 (3/60)	1.10 (0.34–3.57)
Viral infection	0 (0/12)	—
Malaria	8.2 (7/85)	2.10 (0.90–4.87)

RR, risk ratio.

### Identification of patients with severe disease at inclusion

The results of the POCTs are summarized in Table 10. The algorithm classifications are provided in Table 11. Compared to the control ALMANACH algorithm, e-POCT categorized around twice as many patients as having severe disease (and recommended referral) at day 0: 6% (103/1,586) versus 3% (55/1,583) (Table 11). A large proportion of this difference was attributable to the severe malnutrition and severe anemia classifications: e-POCT identified 3.5% (55/1,586) of patients as having severe malnutrition, while ALMANACH identified 0.1% (1/1,583; RD 3.4, 95% CI 2.5, 4.3). With e-POCT, 1.3% (20/1,586) were classified as having severe anemia using Hb testing, versus 0.3% (5/1,583) based on clinical symptoms in the ALMANACH arm (RD 0.9, 95% CI 0.3, 1.6). In the routine care cohort, only 0.4% (2/544) of patients were given a severe diagnosis by the routine clinician (Table 9).

**Table 10:** Results of point-of care tests.

Characteristic	*N*	e-POCT arm	ALMANACH arm
***POCTs for identification of patients with severe disease***			
SaO2 < 90%	1,591	0.3 (4/1,591)	—
Severe tachycardia	1,591	0.4 (6/1,591)	
Hemoglobin (g/l), mean (SD)	1,591	97 (15)	—
Hemoglobin < 60 g/l	1,591	1.3 (20/1,591)	—
***POCTs for identification of patients in need for antibiotic treatment***			
**CRP (mg/l)**	823		
0–9		63.4 (522/823)	—
10–39		28.4 (234/823)	—
40–79		5.8 (48/823)	—
≥80		2.3 (19/823)	—
**PCT (μg/l)**	407		
<0.5		70.3 (286/407)	—
0.5–0.9		8.6 (35/407)	—
1.0–1.9		5.2 (21/407)	—
2.0–3.9		3.9 (16/407)	—
≥4.0		12.0 (49/407)	—
**CRP ≥ 80 mg/l and PCT ≥ 4.0 μg/l**	406	0.7 (3/406)	
**Positive Typhidot, day 0**	152	—	0.7 (1/153)
**Positive urine dipstick^1^**	370		13.5 (50/370)
***Other POCTs***			
mRDT positive	3,182	12.0 (191/1,591)	11.6 (186/1,591)
HIV-1/2 antibody positive	2,917	1.4 (21/1,466)	1.1 (16/1,451)

Values are percent (*n/N*) unless otherwise indicated.

^1^Positive urine nitrite and/or urine leucocyte.

CRP, C-reactive protein; mRDT, malaria rapid diagnostic test; PCT, procalcitonin; POCT, point-of-care test; SaO2, oxygen saturation.

**Table 11:** Algorithm disease classifications.

Disease classification	e-POCT arm, percent (*n*), *N* = 1,586	ALMANACH arm, percent (*n*), *N* = 1,583
**Severe infections**	6.5 (103)	3.5 (55)
CNS danger signs	0.6 (9)	0.7 (11)
Severe pneumonia	0.8 (13)	1.4 (22)
Severe anemia	1.3 (20)	0.3 (5)
Severe malnutrition	3.5 (55)	0.1 (1)
Severe skin infection	NA	0.8 (12)
Severe dehydration	0.4 (6)	0.1 (2)
Other severe disease	NA	0.1 (2)
**Respiratory infections**		
Upper respiratory tract infection	24.7 (391)	34.2 (542)
Clinical pneumonia	NA	20.8 (330)
Viral lower respiratory tract infection	27.0 (429)	NA
Bacterial lower respiratory tract infection	0.6 (10)	NA
**Fever without focus**		
FWS, viral infection	22.0 (349)	22.6 (357)
FWS, bacterial infection^1^	3.3 (52)	4.4 (70)
**Other infections**		
Uncomplicated malaria	10.5 (166)	11.4 (180)
Gastrointestinal infection with dehydration	10.7 (169)	18.7 (296)
Skin infection	4.7 (75)	4.4 (69)

Please refer to Table 1 for criteria used by the respective algorithms for classifications; note that a patient can have more than 1 disease classification.

^1^“FWS, bacterial infection” in the ALMANACH arm was composed of the following diagnoses: urinary, 47; typhoid, 1; ear infection, 22.

CNS, central nervous system; FWS, fever without source; NA, not applicable.

### Antibiotic prescription

In the e-POCT arm, 11.5% (182/1,586) of participants were prescribed an antibiotic treatment at day 0, versus 29.7% (470/1,583) in the ALMANACH control arm (RR 0.39, 95% CI 0.33, 0.45; Table 4). An additional 4% (53/1,404) and 4% (49/1,113) of patients who had not been prescribed an antibiotic at day 0 received antibiotics between day 1 and day 6 in the e-POCT and ALMANACH control arms, respectively. Out of these antibiotic prescriptions after the initial consultation, 47% (25/53) in the e-POCT arm and 65% (32/49) in the ALMANACH arm received antibiotics through consultation with non-study physicians.

**Fig 6:**
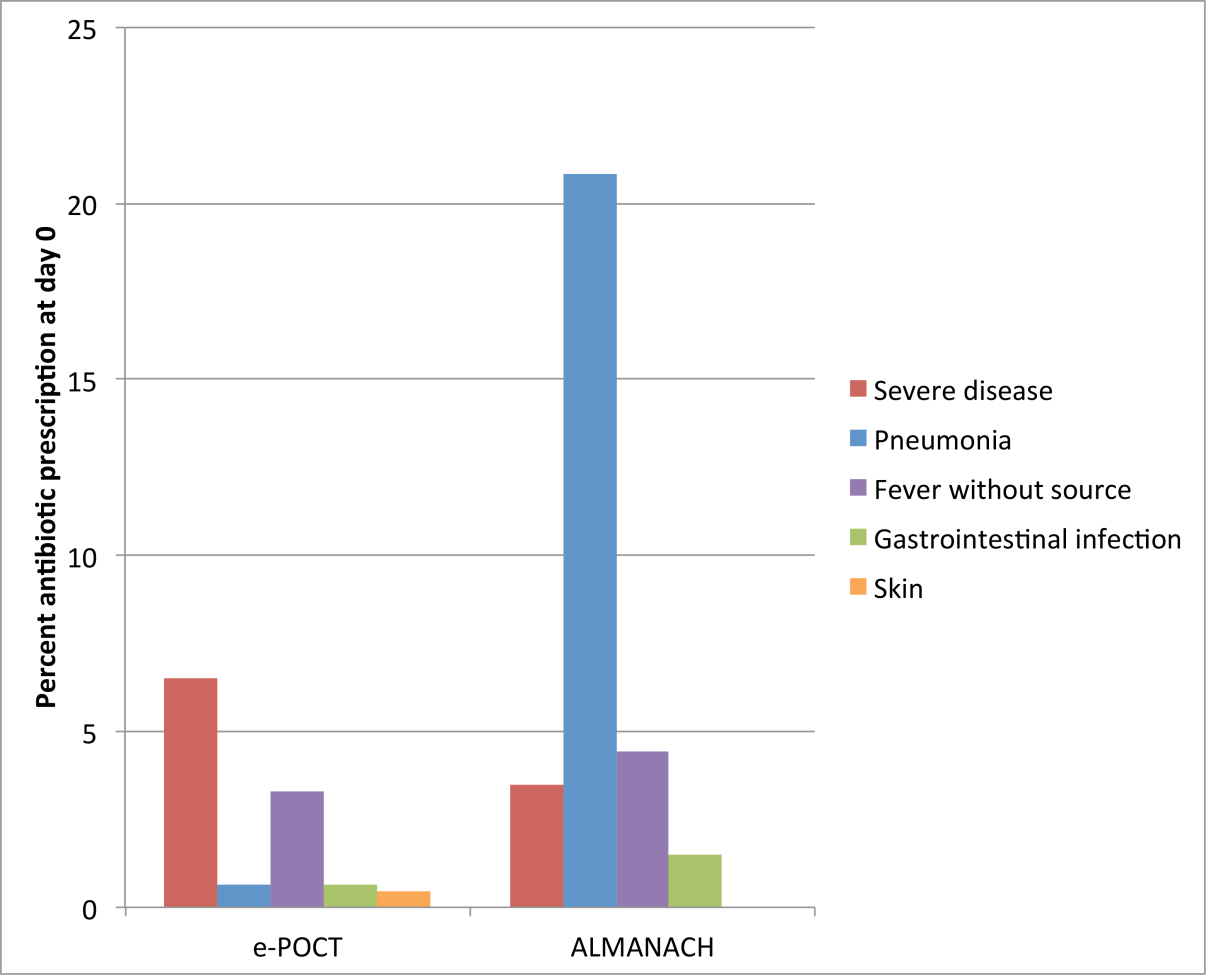
Percent of patients with antibiotic prescription at day 0 according to reason for antibiotic prescription and study arm. For e-POCT and ALMANACH, antibiotic prescription was determined by the algorithm classification.

The majority (57%, 103/182) of antibiotic prescriptions in the e-POCT arm resulted from severe classification (Fig 6). In the ALMANACH control arm, (non-severe) clinical pneumonia (see Table 1 for definition) was the leading classification for antibiotic treatment (70%, 330/470), while a severe classification was the reason for 12% (55/470) of these prescriptions (Fig 6). In contrast to that, bacterial LRTI (see Table 1 for definition) was the classification for only 6% (10/182) of antibiotic prescriptions in the e-POCT arm (Fig 6). Importantly, among patients with non-severe classification, antibiotic prescription was associated with clinical failure in the ALMANACH arm, but not in the e-POCT arm (Table 6). In the routine care cohort, upper respiratory tract infection was the most common diagnosis reported by the routine clinician resulting in antibiotic prescription (47%, 245/516 prescriptions), followed by diarrhea (17%, 88/518 prescriptions) and uncomplicated malaria (12.5%, 65/516 prescriptions).

### Use of point-of-care tests in the e-POCT arm

In all, 0.3% (4/1,591) of children were identified as having hypoxemia, 0.4% (6/1,591) severe tachycardia, and 1.3% (20/1,591) severe anemia (Table 10). Among children with non-severe signs and symptoms, 2.3% (19/1,591) met the CRP cutoff criterion (≥80 mg/l) for antibiotic treatment: out of the 19 children with high CRP (≥80 mg/l), 53% (10) presented with respiratory symptoms, and 47% (9/19) had fever without localizing signs. Another 2.9% (46/1,591) of patients with fever without localizing symptoms and low CRP values had PCT values of ≥4.0 μg/l, hence meeting the criterion for antibiotic treatment. In sum, a total of 13% (52/401) of children with fever without localizing symptoms were classified as having a likely bacterial infection using PCT and CRP testing in e-POCT, compared to 17% (70/424) in the ALMANACH arm using clinical signs, urine dipstick testing, and a rapid typhoid diagnostic test (Fig 6). There was no statistically significant difference in the risk of clinical failure in patients without localizing symptoms between the 2 arms: 0.7% (3/401) for e-POCT versus 1.4% (6/415) for ALMANACH (RR 0.52, 95% CI 0.13, 2.05).

## Discussion

In this multicenter, randomized, controlled non-inferiority trial including 3,739 children with febrile illnesses in Dar es Salam, Tanzania, we showed that e-POCT, a novel patient management algorithm using host biomarker POCTs, was non-inferior to a reference electronic IMCI-based algorithm (ALMANACH) in terms of clinical outcome. e-POCT actually achieved a reduction of 43% in the proportion of clinical failures by day 7 and a reduction of 58% in the proportion of severe adverse events compared to ALMANACH, while substantially lowering the proportion of antibiotic prescriptions from 30% to 11%. e-POCT categorized more patients as having severe disease on day 0. Using e-POCT, the pattern of antibiotic prescription was shifted away from non-severe respiratory infections towards patients with severe disease when compared to the IMCI-based control algorithm. We also observed routine care among children in Dar es Salaam. Compared to children treated per routine care, e-POCT resulted in a 49% reduction in relative risk of clinical failure. In the routine care cohort, 95% of children were prescribed an antibiotic at day 0. The high proportion of antibiotic prescriptions is in line with previous studies in Dar es Salaam [2].

Though the study was designed as a non-inferiority trial, the very low attrition rate makes a type I error unlikely when interpreting the results towards superiority. One could argue that a superiority trial would have been more appropriate from the start. However, we opted for a non-inferiority design, since it had already been demonstrated that ALMANACH was superior to routine care in terms of clinical outcome [2]. In our study, e-POCT also achieved superior clinical outcome compared to routine care. We hence did not expect an additional benefit in terms of clinical outcome. Rather, given the innovative features of e-POCT, we wanted to assess its safety and evaluate whether e-POCT would provide additional benefits in terms of detection of severe illness in children and reduction of antibiotic prescription compared to ALMANACH. We used an individual randomization scheme for the main comparison between e-POCT and ALMANACH, instead of cluster randomization, since the available clusters in the study region were limited. We were concerned about bias from a strong inter-cluster correlation. Given that study clinicians swapped algorithms every 2 weeks, and given that the electronic format guaranteed full algorithm adherence, the overall concern about a bias towards the null was low. The criteria used to define clinical failure were based on previous pneumonia management trials [23,24]. The validity of some of the criteria used for non-severe disease classifications should certainly be reevaluated for future trials. For example, one could argue that having persistent “clinical pneumonia” on day 3 actually does not represent clinical failure but may rather be the natural course of illness of a viral respiratory tract infection [25]. Similarly, patients with persistent fever after several days have in fact a high likelihood of having a viral infection, such as Epstein–Barr virus or *Cytomegalovirus* infection [26]. As expected, “negative outcome from an infection (clinical failure),” as well as severe adverse events (secondary hospitalizations and deaths) were rare in this study conducted in the primary care setting. Nevertheless, the potential implications for health outcomes for children with infections are substantial given the high burden of disease from infections: the number needed to treat to prevent 1 more clinical failure for e-POCT compared to ALMANACH in this study was 57; given that acute febrile illnesses represent the vast majority of consultations in the pediatric outpatient setting in Tanzania, this would correspond to about 1 averted clinical failure per health center per day. We included febrile children only, instead of all children presenting with acute medical complaints, since children without fever have a very low risk of having an infection that requires antibiotics [2]. Consequently, the results should be generalizable even more to non-febrile children.

In terms of using host biomarkers of inflammation for the management of febrile illness, this was the first trial to our knowledge that assessed the safety of using CRP and/or PCT testing to decide on antibiotic prescription in children in a resource-limited country. The use of CRP and PCT testing within an electronic algorithm in our study even improved clinical outcome. One recent open, randomized trial evaluated the impact of using a CRP POC testing in Vietnam among patients with respiratory symptoms, including 287 children less than 6 years of age [18]. This trial did not find a difference in clinical outcome, but was underpowered to detect such a difference because sample size calculations were done based on reduction of antibiotic prescription. Several randomized trials have assessed CRP and PCT for deciding on antibiotic prescription for respiratory infections in adults [27–30]. A series of studies assessed the accuracy of CRP and PCT in diagnosing bacterial infections in children in an outpatient setting in well-resourced settings [17,31]. These studies found moderate diagnostic accuracy for both CRP and PCT. However, one has to bear in mind that diagnostic gold standards are often imperfect. For example, for pneumonia, the most recognized current gold standard is “WHO endpoint pneumonia,” i.e., consolidation on chest radiograph [32]. However, only a (undefined) proportion of WHO endpoint pneumonia is bacterial in origin. Besides the 1 trial in Vietnam that included both children and adults, all studies of CRP and PCT in children have focused on analytical performance; none have assessed whether using these tests would change patient outcome. Though the POCTs used by e-POCT are used widely in pediatric practice, they are not available in routine care in Tanzania as of yet. Having the POCTs available, as on a single lateral-flow platform, would be ideal. This will be part of future efforts to make e-POCT scalable.

In terms of impact on antibiotic prescription when using host biomarker POCTs, a much smaller effect in terms of reduction in antibiotic prescription can be expected when these POCTs are provided without guidance (i.e., performed on all patients). For example, in the Vietnam trial, the reduction of antibiotic prescription observed was from 74% to 68% by day 14 [18]. Such a small reduction in prescription rate could be achieved on clinical grounds alone: in our study 43% (392/920) of patients in the e-POCT arm with cough did not meet clinical criteria for CRP testing and were categorized as having an upper respiratory infection. This reduction in antibiotic prescriptions achievable by the use of clinical signs alone was also demonstrated in a cluster-randomized trial assessing the use of ALMANACH [2]. Similarly, a recent cluster-randomized trial in pediatric primary care in Belgium assessing the use of CRP as a screening tool to rule out serious bacterial infection concluded that only children at higher risk for serious bacterial infection after clinical assessment should be tested using CRP [33].

To make the best use of CRP and PCT testing, we decided to include them in a patient management tool. It guided clinicians on which patients to select for testing and how to use the result in the clinical context. We also employed higher cutoffs (80 mg/l for CRP and 4 ug/l for PCT) to rule in patients likely require antibiotic treatment (instead of ruling out patients with a very low probability of having a bacterial infection using a lower CRP cutoff, such as 10 mg/l in the Vietnam trial [18]). This approach was based on analyses of host biomarker results from a Tanzanian study of the etiology of fever [32] and on the fact that patients in outpatient settings have a very low pre-test probability of having a bacterial infection [15]. e-POCT indeed selected few patients as requiring antibiotic treatment with this strategy: 1.2% (10/833) of patients with non-severe respiratory classifications and 13% (53/402) of patients with fever without localizing symptoms.

e-POCT also uses POCTs for identifying children with severe disease: an oximeter to detect hypoxemia and severe tachycardia, and a POC hemoglobinometer to detect severe anemia. e-POCT indeed classified around twice as many children as having severe disease than the control algorithm. However, this was only partially due to the POCTs employed: only very few children with hypoxemia and/or severe tachycardia were identified. The use of oximeters as part of IMCI has been advocated. However, their utility at the peripheral healthcare level should be assessed further [34]. e-POCT diagnosed 4 times as many patients with severe anemia than the IMCI-based control algorithm (and only 1/5 patients given a severe anemia diagnosis using the control algorithm actually had a low Hb value). This confirms findings from previous studies that severe anemia cannot be detected using clinical signs [35]. However, severe anemia, in turn, is an important risk factor for death from severe infections and is associated with severe infections [36,37]. Given that children cannot be preselected for Hb testing based on clinical elements, all children would have to undergo Hb testing to detect the few children with severe anemia. This strategy will become feasible only once low-cost Hb tests can be deployed to the peripheral healthcare level—if possible, tests that can be directly connected to tablets. e-POCT also detected and referred more than 20 times as many children with a severe malnutrition classification using combined weight-for-age and mid-upper arm circumference testing (compared to clinical signs in the control arm). Severe malnutrition is recognized as an important risk factor for severe outcome for infections [38–40]. Our findings corroborate the 2014 IMCI recommendations (which had not been implemented in Tanzania at the time of the study) to use anthropometric criteria in addition to clinical signs to detect children with severe malnutrition. Overall, the adequacy of the referral criteria could not be assessed in this study, which is certainly a limitation. This is because decisions to admit a child were made by providers outside of the study, and no predefined criteria were used. Hospital-based care is often low in quality and not standardized. As a result, neither admission decisions nor admission diagnoses could be used as a diagnostic gold standard [41]. In terms of routine care, the comparison between the e-POCT arm and routine care had an important limitation in that we did not take any measures in the routine care cohort (such as provision of guidelines and additional training). In contrast, study clinicians in the e-POCT arm were asked to adhere to the e-POCT algorithm for treating patients. This comparison, however, may give an impression of e-POCT’s maximum achievable public health benefit in terms of clinical outcome and antibiotic prescription.

Overall, our data provide evidence that e-POCT not only reduced the proportion of antibiotic prescriptions but also increased the targeting of children in need for antibiotic treatment compared to ALMANACH. Antibiotic treatment was indeed shifted away from non-severe respiratory infections towards severe disease classifications. Furthermore, in addition to the overall improved clinical outcome using e-POCT, antibiotic prescription was associated with clinical failure in the ALMANACH control arm, but not in the e-POCT arm. This may be because children who received unnecessary antibiotic prescription in the ALMANACH control arm experienced antibiotic side effects, or the neglect of other important supportive treatments for viral infection (such as bronchodilator treatment or rehydration). This interpretation is further underlined by our observation that time to resolution of fever did not differ between the 2 arms, while clinical failure did. The vast majority of children in our study likely had viral infections. Since we did not use any antiviral agents in the study, the overall duration of the illness could not be altered using either electronic algorithm. However, better supportive care (such as provision of hydration and bronchodilator treatment) in the e-POCT arm likely resulted in a lower rate of complications from viral infections (such as dehydration or severe respiratory distress).

Our study has several limitations that need to be addressed in further studies. First, this was a multicenter but single region study, which limits the generalizability of our findings. Given the innovative character of e-POCT, we opted for a study setting where good oversight and pediatric backup could be guaranteed. Key algorithm components should be reassessed in different geographical settings and populations with higher HIV and malnutrition rates. Second, there are concerns about using CRP testing to detect bacterial infections in patients with malaria (malaria infection in itself leads to high levels of CRP and PCT [31,42]). However, antibiotic prescription is generally not indicated in patients with uncomplicated malaria. Bacterial co-infections are nevertheless common in patients with severe malaria [43]. Using enhanced severity criteria, such as Hb testing, may actually improve detection of severe malaria and may thus help to identify children in need of concomitant antimalarial and antibiotic treatment. Third, equipment-based treatment, rather than treatment based on clinical grounds alone, is faced with challenges in terms of supply chain, and possibly cost. Compared to ALMANACH and to routine care, the additional components required for running e-POCT are an oximeter, POC Hb testing, as well as POC CRP and PCT testing in selected subgroups. On the other hand, e-POCT does not require urine testing like ALMANACH. Tablets should also be provided for both e-POCT and ALMANACH; they are now becoming available for other tasks in many health facilities. However, the costs associated with such additional components may be outweighed by the costs that are associated with antibiotic overprescription on an individual and societal level [44]. Finally, the true utility of e-POCT can only be evaluated through open implementation studies since adherence to the algorithm will be an important factor in making use of its advantages in terms of clinical outcome and antibiotic prescription.

## Conclusion

e-POCT, an innovative electronic algorithm using host biomarker POCTs, has the potential to improve clinical outcome for children with febrile illnesses in low-resource settings while reducing antibiotic use through improved identification of children with severe infections and increased targeting of children in need of antibiotic prescriptions. Using CRP and PCT cutoffs, integrated into an overall disease management algorithm, for the management of children with respiratory infections and FWS was safe in terms of clinical outcome. Notwithstanding the need for replication of these findings in other geographical settings and further implementation studies, our results provide first evidence that such an innovative patient management approach is beneficial. Electronic algorithms in general are important tools to increase compliance with IMCI—the integration of POCTs would make even better use of such technologies. A key advantage of using host biomarker tests, as compared with a series of disease etiology tests, is that such an approach is likely more robust to seasonal and geographical variations in disease etiology. POCTs should include tests both for identification of patients with severe disease (e.g., severe anemia) and for detection of bacterial infections (such as CRP and PCT). To make the best use of these POCTs, they should be integrated into a patient management tool that helps clinicians not only select patient subgroups for which testing is useful, but also interpret results within an overall patient assessment. This will also allow the continuation of an integrated approach to the treatment of childhood infections as advocated by IMCI.

## Supporting information

S1 Fig Flowchart of literature search results and included publications. (PDF)

S2 Fig Schematic representation of ALMANACH algorithm. (PDF)

S3 Fig Days to resolution of fever (e-POCT arm and routine care cohort). (TIF)

S1 Table CONSORT checklist. (DOC)

S2 Table Literature search terms. (DOCX)

S3 Table Primary and secondary study outcomes for randomized study (intention-to-treat population). (DOCX)

S4 Table Mixed effects logistic regression (randomized study). (DOCX)

S5 Table Mantel–Haenszel estimates of the effect of clinician and health center on primary and secondary outcome measures (randomized study). (DOCX)

S6 Table Primary and secondary study outcome comparisons between the e-POCT arm and the routine care cohort (intention-to-treat population). (DOCX)

S1 Text Study protocol. (PDF)

S2 Text Statistical analysis plan. (PDF)

S3 Text Detailed description and discussion of the methods and evidence used for the development of the e-POCT algorithm. (DOCX)

## Abbreviations

CNS: central nervous system
CRP: C-reactive protein
FWS: fever without source
Hb: hemoglobin
HIV: human immunodeficiency virus
IMCI: Integrated Management of Childhood Illness
ITT: intention-to-treat
LRTI: lower respiratory tract infection
mRDT: malaria rapid diagnostic test
MUAC: mid-upper arm circumference
OR: odds ratio
PCT: procalcitonin
POC: point-of-care
POCT: point-of-care test
PP: per-protocol
RD: risk difference
RR: risk ratio
SaO2: oxygen saturation
WFA: weight-for-age
WFH: weight-for-length/height
WHO: World Health Organization
