# Supplementary material for: A novel electronic algorithm using host biomarker point-of-care tests for the management of febrile illnesses in Tanzanian children (e-POCT): A randomized, controlled non-inferiority trial
Source: PLoS Med. 2017 Oct 23;14(10):e1002411. doi: 10.1371/journal.pmed.1002411 (PMC5653205; doi:10.1371/journal.pmed.1002411)
Supplement: S2 Fig — (PDF) [file pmed.1002411.s002.pdf]

ALMANACH:

A new ALgorithm for the MANAgement of CHildhood illnesses

For children aged 2 months up to 5 years

PeDiAtrick project 2009 - 2012

Schematic representation for e-POCT study (control arm)

severe disease, IM antibiotic/antimalarial treatment	oral antibiotic/antimalarial treatment recommended	no antibiotic/antimalarial treatment recommended

MANAGEMENT OF **VERY SEVERE DISEASES**

CHECK FOR GENERAL DANGER SIGNS

Is the child:

convulsing now?

- Yes → **Give:** Anticonvulsant (Diazepam)
  - AND → Very severe disease (below)
- No ↓

having any General Danger sign?

LOOK
Lethargic/unconscious
Severe pallor
Jaundice
Cyanosis
Stiff neck
Severe wasting

ASK
Unable to drink/breastfeed
Vomits everything
Had convulsions (in the current illness)

- Yes →

Very severe disease:

The child needs **URGENT REFERRAL TO HOSPITAL**

Give PRE-REFERRAL TREATMENT:

First dose of IM ceftriaxone
IM artesunate
Low blood sugar prevention

AND

R E F E R   U R G E N T L Y

Exit

- No ↓

Does the child have **some Pallor?**

- Yes → **Anemia**
- No ↓

Does the child have **Fever ?**
By history
Or feels hot
Or axillary's temperature above 37°5C

- Yes → Duration of fever?
  - 7 days or more → **Persistent fever** → Refer for assessment *Exit*
  - Less than 7 days → Go to [illegible]: Assessment of febrile acute illnesses
- No → Go to [illegible]: Assessment of acute non febrile illnesses

Exit *If you reach this sign, you should refer the child witout completing the assessment.*
If nothing is specified you have to complete the entire assessment before prescribing appropriate treatment.

ASSESSMENT OF FEBRILE CHILDHOOD ILLNESSES

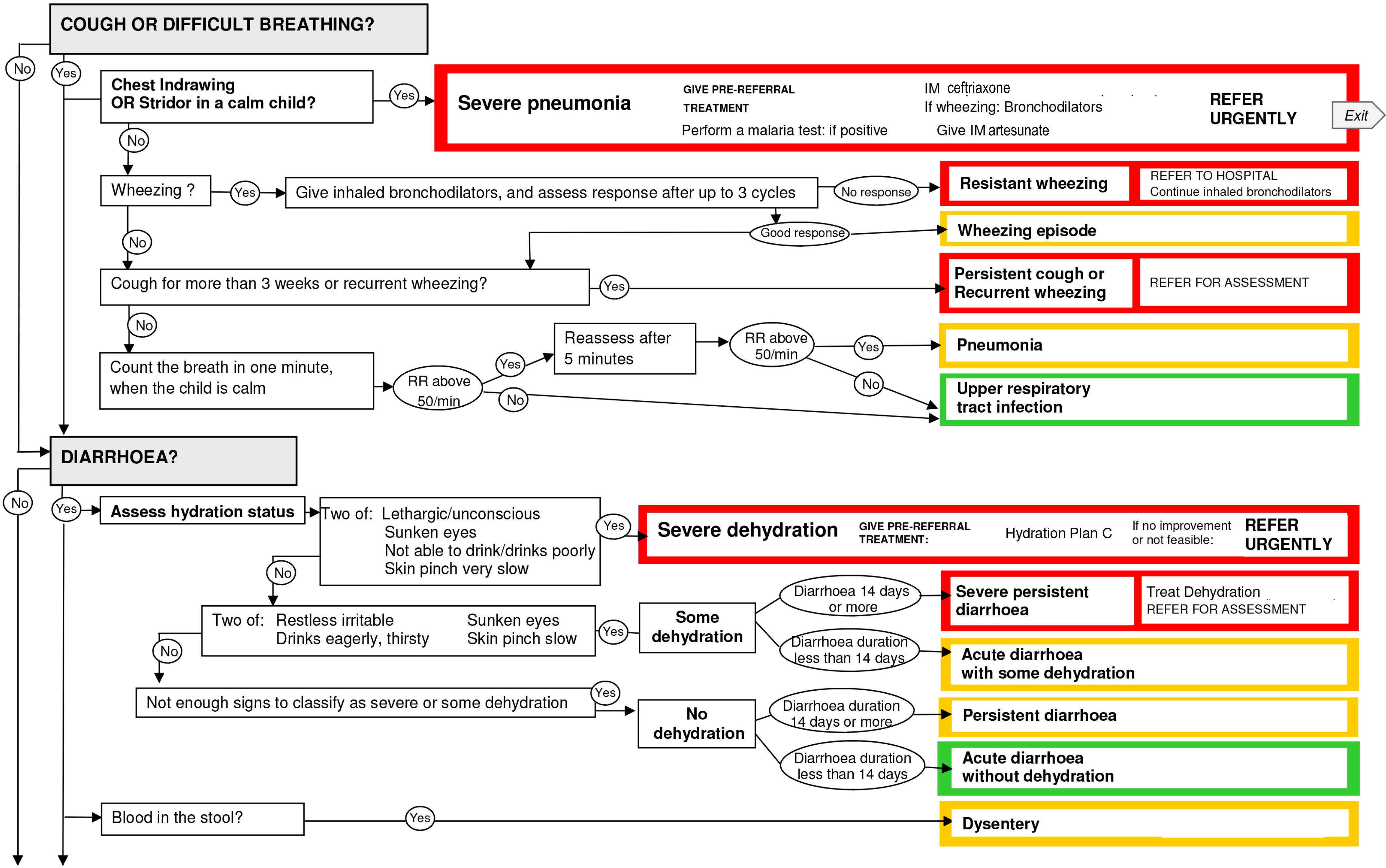

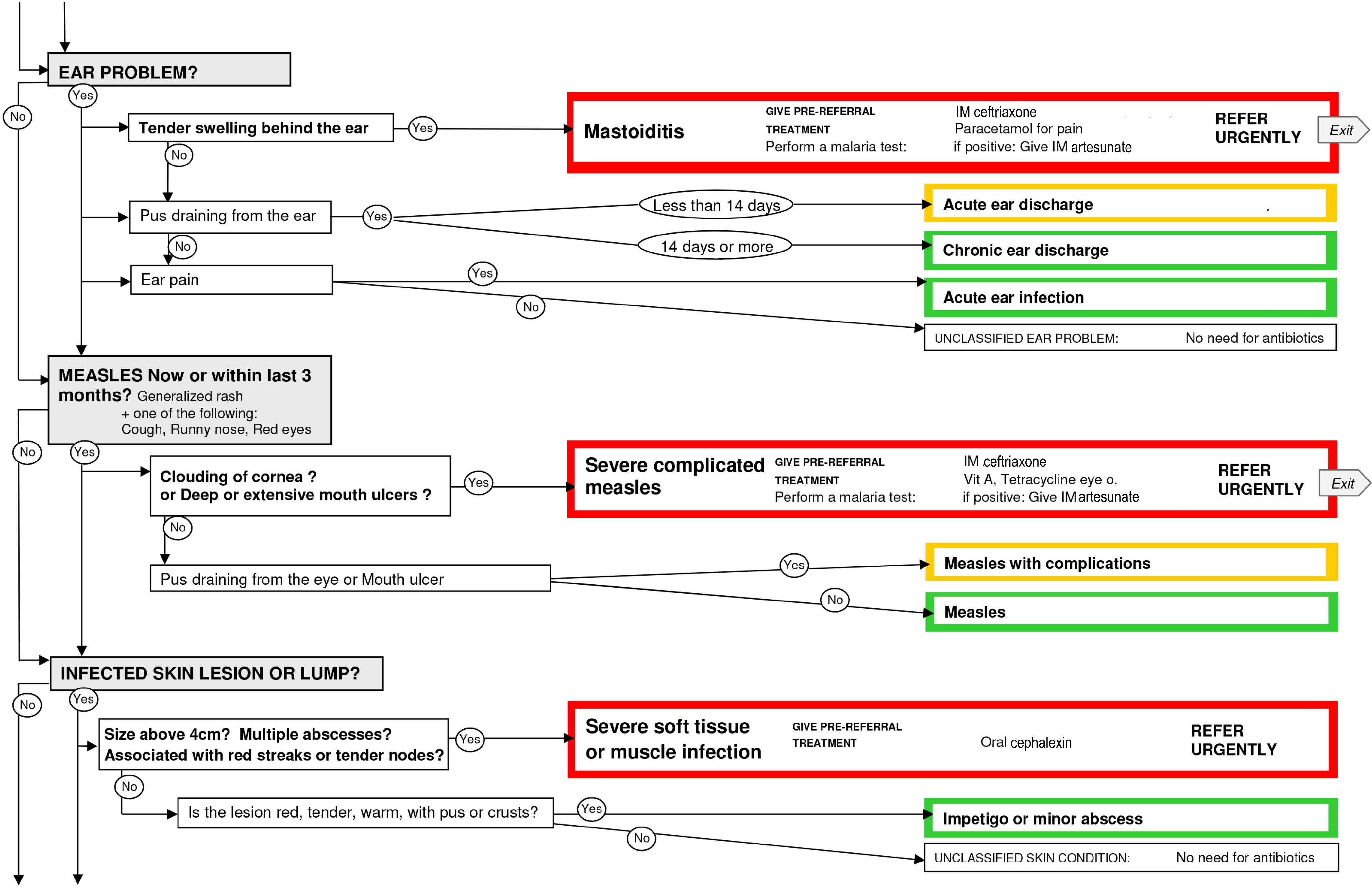
EAR PROBLEM?
Yes
No
Tender swelling behind the ear
Yes
No
Mastoiditis
GIVE PRE-REFERRAL TREATMENT
IM ceftriaxone
Paracetamol for pain
Perform a malaria test:
if positive: Give IM artesunate
REFER URGENTLY
Exit
Pus draining from the ear
Yes
No
Less than 14 days
14 days or more
Acute ear discharge
Chronic ear discharge
Ear pain
Yes
No
Acute ear infection
UNCLASSIFIED EAR PROBLEM:
No need for antibiotics
MEASLES Now or within last 3 months? Generalized rash
+ one of the following:
Cough, Runny nose, Red eyes
No
Yes
Clouding of cornea ?
or Deep or extensive mouth ulcers ?
Yes
No
Severe complicated measles
GIVE PRE-REFERRAL TREATMENT
IM ceftriaxone
Vit A, Tetracycline eye o.
Perform a malaria test:
if positive: Give IM artesunate
REFER URGENTLY
Exit
Pus draining from the eye or Mouth ulcer
Yes
No
Measles with complications
Measles
INFECTED SKIN LESION OR LUMP?
No
Yes
Size above 4cm? Multiple abscesses?
Associated with red streaks or tender nodes?
Yes
No
Severe soft tissue or muscle infection
GIVE PRE-REFERRAL TREATMENT
Oral cephalexin
REFER URGENTLY
Is the lesion red, tender, warm, with pus or crusts?
Yes
No
Impetigo or minor abscess
UNCLASSIFIED SKIN CONDITION:
No need for antibiotics

ASSESSMENT OF **FEBRILE** CHILDHOOD ILLNESSES

After this assessment **if the child has fever with no identified cause, perform the following:**

For children aged 2 months up to less than 2 years: → Urine dipstick

For children aged 2 years up to 5 years: → If dysuria → Urine dipstick

Urine dipstick:
- Leucocytes OR Nitrites positive → **Urinary tract infection (UTI)**
- Leucocytes AND Nitrites negative → **No Urinary tract infection**

For children aged 2 years up to 5 years: → Typhoid rapid diagnostic test

Typhoid rapid diagnostic test:
- Positive → **Possible intestinal bacterial disease**
- Negative → **Bacterial disease unlikely**

FOR ALL CHILDREN WITH FEVER OR HISTORY OF FEVER: CONSIDER MALARIA

CONSIDER MALARIA: Perform a malaria RAPID DIAGNOSIS TEST (mRDT)
If mRDTs are not available, perform a blood-slide.

- Positive → **Malaria** (oral antimalarial treatment)
- Negative → No Malaria

If you answered NO to all the questions, the child has fever with no obvious cause, no danger sign, and the malaria test is negative.
The child is likely to have a **Viral infection**. S/he does NOT need neither antibiotic nor antimalarial.
Prescribe symptomatic treatment for fever
Reassure the caretaker and advise him/her to return ımmediately if the child is not able to drink or becomes sicker.
Advise him/her to come back after 2 days if fever persists.
