# Supplementary figures and images for: A novel electronic algorithm using host biomarker point-of-care tests for the management of febrile illnesses in Tanzanian children (e-POCT): A randomized, controlled non-inferiority trial
Source: PLoS Med. 2017 Oct 23;14(10):e1002411. doi: 10.1371/journal.pmed.1002411 (PMC5653205; doi:10.1371/journal.pmed.1002411)

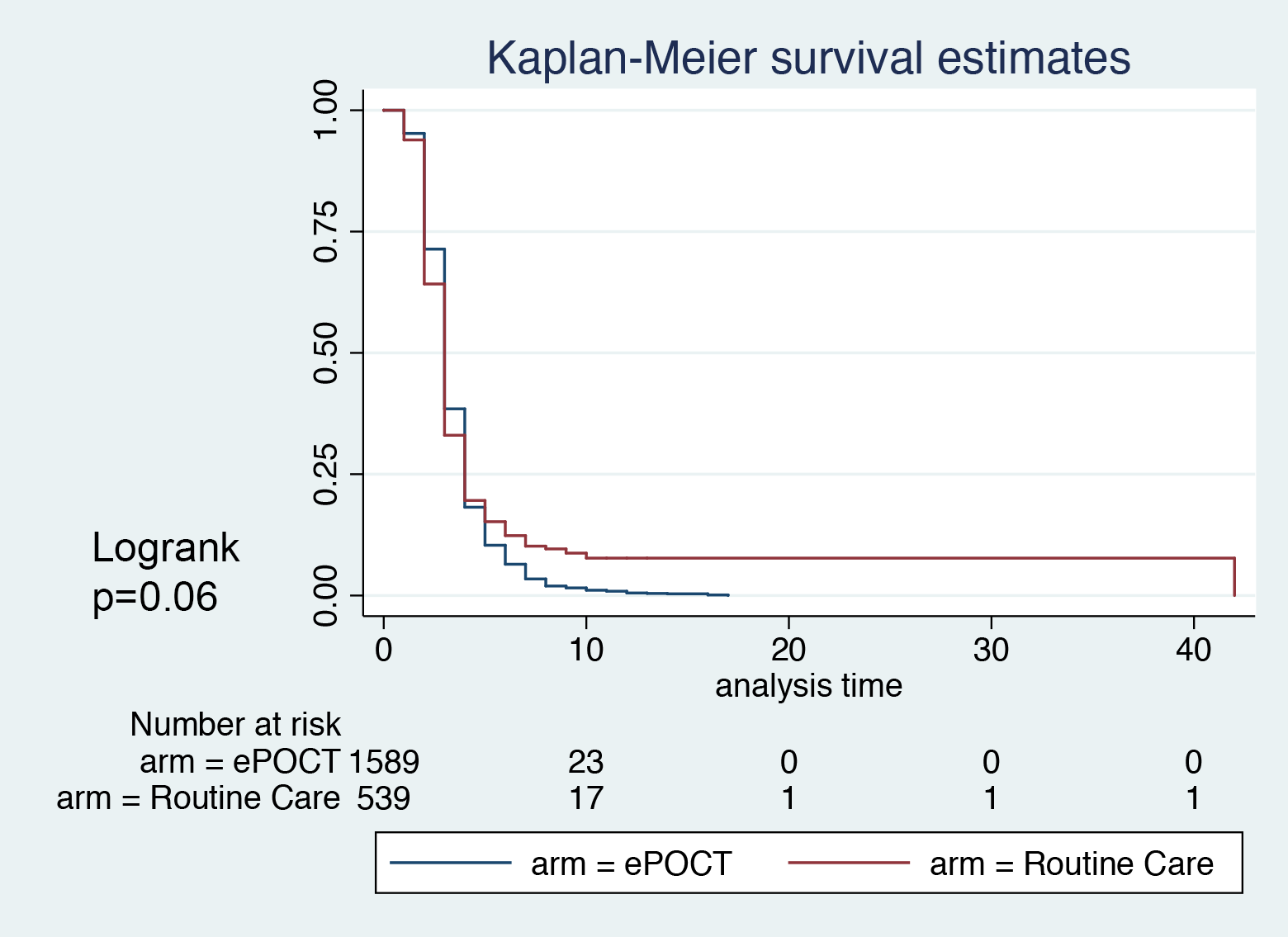

Supplement: S3 Fig — (TIF) [file pmed.1002411.s003.tif]
