# Supplementary material for: A novel electronic algorithm using host biomarker point-of-care tests for the management of febrile illnesses in Tanzanian children (e-POCT): A randomized, controlled non-inferiority trial
Source: PLoS Med. 2017 Oct 23;14(10):e1002411. doi: 10.1371/journal.pmed.1002411 (PMC5653205; doi:10.1371/journal.pmed.1002411)
Supplement: S3 Table — (DOCX) [file pmed.1002411.s006.docx]

| **S3 Table: Primary and secondary study outcomes for randomized study (ITT)** | | | | | |
| --- | --- | --- | --- | --- | --- |
|  | | **e-POCT**  **% (n/N)** | **ALMANACH**  **% (n/N)** | **Risk Difference**  **(95% CI)** | **Risk Ratio**  **(95% CI)** |
| **Primary Outcome** | |  |  |  |  |
| Clinical Failure by day 7 | | 2.9 (47/1596) | 4.9 (78/1596) | -1.9 (-3.3, -0.6) | 0.60 (0.42-0.86) |
| **Secondary Outcomes** | |  |  |  |  |
| Primary referrals | | 7.1 (114/1596) | 3.7 (59/1596) | 3.4 (1.9, 5.0) | 1.93 (1.42, 2.62) |
| Antibiotic prescription at day 0 | | 12.0 (192/1596) | 30.2 (483/1596) | -18.2 (-21.0, -15.5) | 0.40 (0.34, 0.46) |
| Severe adverse events by day 30 | | 1.3 (20/1596) | 2.3 (37/1596) | -1.1 (-1.9, -0.1) | 0.54 (0.32, 0.93) |
|  | Secondary admissions | 1.1 (17/1596) | 2.0 (32/1596) | -0.9 (-1.8, -0.8) | 0.53 (0.30, 0.95) |
|  | Deaths | 0.8 (13/1596) | 1.2 (19/1596) | -0.4 (-1.0, 0.3) | 0.68 (0.33, 1.38) |
