# Supplementary material for: A novel electronic algorithm using host biomarker point-of-care tests for the management of febrile illnesses in Tanzanian children (e-POCT): A randomized, controlled non-inferiority trial
Source: PLoS Med. 2017 Oct 23;14(10):e1002411. doi: 10.1371/journal.pmed.1002411 (PMC5653205; doi:10.1371/journal.pmed.1002411)
Supplement: S4 Table — (DOCX) [file pmed.1002411.s007.docx]

| **S4 Table: Mixed effect logistic regression (randomized study)** | | | | |
| --- | --- | --- | --- | --- |
|  | **Naïve Model** | | **Full Model** | |
| **Predictor** | **OR (exp B)** | **95% CI** | **OR (exp B)** | **95% CI** |
| Constant | 0.04*** | 0.37-0.84 | 1.07 ^-9^*** | 1.98^-14^-0.00 |
| **e-POCT intervention** | **0.56**** | **0.37-0.84** | **0.55**** | **0.36-0.84** |
| Clinician 1 |  |  | Reference |  |
| Clinician 2 |  |  | 1.78 | 0.56-5.60 |
| Clinician 3 |  |  | 1.03 | 0.32-3.37 |
| Clinician 4 |  |  | 0.60 | 0.29-1.24 |
| Age (month) |  |  | 0.98 | 0.96-1.00 |
| Weight for age z-score |  |  | 0.72*** | 0.62-0.85 |
| Body temperature (°C) |  |  | 1.52** | 1.14-2.04 |
| Respiratory rate |  |  | 1.03** | 1.01-1.05 |
| Variance health center level |  |  | **Estimate** | **SE** |
|  |  |  | 0.34 | 0.24 |
| Log likelihood | -447 | | -392 | |
