# Supplementary material for: A novel electronic algorithm using host biomarker point-of-care tests for the management of febrile illnesses in Tanzanian children (e-POCT): A randomized, controlled non-inferiority trial
Source: PLoS Med. 2017 Oct 23;14(10):e1002411. doi: 10.1371/journal.pmed.1002411 (PMC5653205; doi:10.1371/journal.pmed.1002411)
Supplement: S5 Table — (DOCX) [file pmed.1002411.s008.docx]

| **S5 Table: Results of Mantel–Haenszel estimates of the effect of clinician and health centers on primary and secondary outcome measures (randomized study)** | | |
| --- | --- | --- |
|  | **Mantel-Haenszel estimate (Pr>chi^2^)** | |
| **Outcome (PP)** | **Health Center** | **Clinician** |
| Clinical failure by day 7 | 0.57 | 0.71 |
| Primary referrals | 0.07 | 0.65 |
| Antibiotic prescription | 0.25 | 0.70 |
| Severe adverse events (total) | 0.97 | 0.82 |
| Secondary hospitalizations | 0.78 | 0.57 |
| Deaths | 0.77 | 0.92 |
