# Supplementary material for: A novel electronic algorithm using host biomarker point-of-care tests for the management of febrile illnesses in Tanzanian children (e-POCT): A randomized, controlled non-inferiority trial
Source: PLoS Med. 2017 Oct 23;14(10):e1002411. doi: 10.1371/journal.pmed.1002411 (PMC5653205; doi:10.1371/journal.pmed.1002411)
Supplement: S6 Table — (DOCX) [file pmed.1002411.s009.docx]

| **S6 Table: Primary and secondary study outcomes for comparison between e-POCT arm and routine care (ITT)** | | | | | |
| --- | --- | --- | --- | --- | --- |
|  | | **e-POCT**  **% (n/N)** | **Routine Care**  **% (n/N)** | **Risk Difference**  **(95% CI)** | **Risk Ratio**  **(95% CI)** |
| **Primary Outcome** | |  |  |  |  |
| Clinical Failure by day 7 | | 2.9 (47/1596) | 5.1 (28/547) | -2.1 (-4.2, -0.1) | 0.58 (0.36-0.91) |
| **Secondary Outcomes** | |  |  |  |  |
| Primary referrals | | 7.1 (114/1596) | 0.9 (5/547) | 6.2 (4.7, 7.7) | 7.81 (3.21, 19.03) |
| Antibiotic prescription at day 0 | | 12.0 (192/1596) | 94.9 (28/547) | -82.9 (-85.3, -80.4) | 0.13 (0.11, 0.14) |
| Severe adverse events by day 30 | | 1.3 (20/1596) | 1.6 (9/547) | -0.4 (-1.5, 0.8) | 0.76 (0.34, 1.66) |
|  | Secondary admissions | 1.1 (17/1596) | 1.6 (9/547) | -0.5 (-1.8, 0.5) | 0.65 (0.29, 1.44) |
|  | Deaths | 0.8 (13/1596) | 0.7 (4/547) | 0 (-0.7, 0.9) | 1.11 (0.36, 3.40) |
