# Supplementary material for: A novel electronic algorithm using host biomarker point-of-care tests for the management of febrile illnesses in Tanzanian children (e-POCT): A randomized, controlled non-inferiority trial
Source: PLoS Med. 2017 Oct 23;14(10):e1002411. doi: 10.1371/journal.pmed.1002411 (PMC5653205; doi:10.1371/journal.pmed.1002411)
Supplement: S1 Text — (PDF) [file pmed.1002411.s010.pdf]

Electronic algorithms based on host biomarkers Point of Care Tests to decide on admission and antibiotic prescription in Tanzanian febrile children (e-POCT)

Study Protocol

**Version Number**	4.2	**Document Date**	12 July 2015
**Principle Investigator**	Valérie D'Acremont, +41795562551, valerie.dacremont@unibas.ch		
**Co-Principal investigator**	Frank Kagoro, +255 782243001, fkagoro@ihi.or.tz		
**Co-Principal investigator**	Kristina Keitel-Hasler, +255788816306, kristina.keitel@unibas.ch		
**Co-Principal investigator**	Willy Sangu, +255 22 2128800, wsangu@yahoo.com		
**Funding Agency**	Swiss National Science Foundation		

Confidentiality Statement

This document contains confidential information. The contents may not be used, divulged or published without prior written consent of the principal investigator of the study.
This information cannot be used for any other purpose than the conduct and evaluation of the clinical investigation by the investigator(s), the regulatory authorities and members of the ethics committees.

1 GENERAL INFORMATION

I. List of investigators and other persons involved

Names	Title	Institution	Position	Function in study
Valérie D'Acremont	MD, PhD	Swiss Tropical and Public Health Institute Switzerland	Senior Lecturer of Tropical and Infectious diseases	Leader in Switzerland
Kristina Keitel-Hasler	MD, MPH	Boston Children's Hospital, USA<br>Harvard Medical School, USA<br>Swiss TPH, Switzerland	Clinical Fellow, Infectious Diseases<br>Research Fellow<br>Guest Scientist	Leader in Tanzania
Frank Kagoro	MD	Ifakara Health InstituteTanzania	Research Officer	Collaborator
Willy Sangu	DDMO, Paediatric	Ilala Municipal Council, Dar es Salaam, Tanzania	Curative Coordinator	Collaborator
Blaise Genton	MD, PhD	Swiss TPH, Switzerland	Professor of Tropical and internal Medicine	Collaborator
Kevin Kain	MD, PhD	Toronto University, Canada	Director, Sandra Rotman Centre for Global Health	Biomarkers laboratory work
Amanda Ross	PhD	Swiss TPH, Switzerland	Statistician	Statistical analyses
Aurelio Di Pasquale	PhD	Swiss TPH, Switzerland	Bioinformatics	Programing and data management
Alain Gervaix	MD	Geneva University Hospital, Switzerland	Director, Pediatric Emergency Department	Clinical Advisor

II. Signatures

Name	Signature	Date

III. Table of Contents

1 General Information 2

2 Background information and rationale, preliminary data 9

2.1 Infections are the main cause of under-five mortality 9

2.2 Shortcomings of current clinical algorithms for the management of fever in children 9

2.3 Changes in disease patterns and drug resistance: decrease in malaria prevalence, increase in bacterial drug resistance 10

2.4 Less febrile episodes caused by malaria: what are the causes of 'non-malaria fevers'? 11

2.5 The path towards improved fever management algorithms 12

2.6 Rationale for the clinical study design 24

3 Clinical Study Objectives and Hypotheses 24

3.1 Objectives 24

3.2 Hypotheses 25

4 Study design 25

4.1 Primary and secondary outcome measures 25

4.2 Design description 26

4.3 Intervention 28

4.4 Measure to minimize bias 29

5 Study population 30

5.1 Study site and source population 30

5.2 Enrolment 30

5.3 Inclusion criteria 30

5.4 Exclusion criteria 31

5.5 Definition of clinical failure 31

5.6 Criteria for discontinuation of the study 31

5.7 Participants in sub-cohort 32

6 Study procedures 32

6.1 Clinical study schedule 32

6.2 Screening 32

6.3 Enrolment 33

6.4 Inclusion visit 33

6.5 Scheduled follow-up visits 33

6.6 Unscheduled Follow-up Visits 33

6.7 Final follow-up Interview 33

6.8 Participant Evaluation 33

6.9 Laboratory tests 36

6.10 Clinical care 37

7 Assessment of safety **38**

8 Statistics **38**

8.1 Definition of outcome measures *38*

8.2 Justification of number of study subjects *38*

8.3 Statistical analysis *39*

8.4 Data management *39*

9 Duties of the investigator **40**

9.1 Investigator's confirmation *40*

9.2 Damage coverage *40*

10 Ethical considerations **40**

10.1 Independent Ethics Committee (IEC) *41*

10.2 Evaluation of the risk-benefit ratio *41*

10.3 Subject information and consent *41*

10.4 Subject Confidentiality *41*

10.5 Subjects requiring particular protection *42*

10.6 Compensation for inconvenience *42*

11 Quality control and quality assurance **42**

11.1 Data and safety monitoring board *42*

12 Capacity buiding at the Ifakara Health Institute and the Swiss TPH **42**

13 Dissemination of results and publication policy **43**

13.1 Dissemination to scientific community *43*

13.2 Information of community and policy makers *43*

14 Literature **44**

15 Appendices **51**

15.1 Consent form *51*

15.2 Data collection forms *51*

IV. Abbreviations / Glossary of Terms

Abbreviations:

ARI	Acute respiratory infection
CAP	Community-acquired pneumonia
CLSI	Clinical and Laboratory Standards Institute
CRP	C-reactive protein
CXR	Chest radiograph
EBV	Ebstein-Barr Virus
EUCAST	European Committee on Antimicrobial Susceptibility Testing
Hb	Hemoglobin
IHI	Ifakara Health Institute
IMCI	Integrated Management of Childhood Illnesses
IV	intravenous
OPD	Outpatient department
PCT	Procalcitonin
POCT	Point of Care Test
RDT	Rapid Diagnostic Test
RR	Respiratory Rate
SaO2	Oxygen Saturation
SBI	Serious bacterial infection
Swiss TPH	Swiss Tropical and Public Health Institute
WHO	World Health Organization

Definitions of terms used for the purpose of the present study:

Life-threatening infection	Infection likely to lead to death if left untreated or infection with high risk of complications or sequelae.
Serious bacterial infection	Sepsis due to bacteremia (including typhoid), other systemic bacterial infections (e.g. rickettsiosis, leptospirosis), urinary tract infection, bacterial pneumonia, bacterial meningitis, other localized bacterial infections with high risk of complications (e.g osteomyelitis).
Unspecific fever	Acute febrile illness for which no clear source of infection can be identified on the basis of the clinical assessment and a malaria POCT. This includes children with no history or present cough, no history or present diarrhea and no significant skin infection.
Diarrhea	Three or more unformed stools per day.
Significant skin/ soft tissue infection	Skin (or mucosal) infection severe enough to be the possible cause of the fever and thus requiring antibiotic treatment. The following are considered significant skin/soft tissue infections:<br>• an abscess of more than 4 cm<br>• multiple abscesses<br>• abscess located on the face<br>• extensive impetigo<br>• cellulitis<br>• pyomyositis
Rational use of antimicrobials	Antimicrobials are used rationally when a child with an infection requiring an antibiotic or antimalarial treatment is prescribed such a medicine and when a child with an infection <u>not</u> requiring an antibiotic or antimalarial treatment is <u>not</u> prescribed such a medicine.

Synopsis

**Study Title**	Electronic algorithms based on host biomarkers Point of Care Tests to decide on admission and antibiotic prescription in Tanzanian febrile children (e-POCT).
**Indication**	Acute febrile illness in a child aged 2 months to less than 5 years
**Intervention**	Use of the e-POCT tool for the clinical management of febrile episodes. The e-POCT tool is an electronic algorithm that integrates key clinical elements with the results of malaria and host biomarkers point-of-care test results (including oximetry).
**Study Rationale**	Infections continue to be the main cause of child mortality, especially in resource-poor settings. Health professionals in developing countries have limited ability to identify correctly those children at risk of dying and those in need of antibiotics among children presenting with acute fever to outpatient health facilities. The main reasons for this shortcoming are limited clinical skills and time, unavailability of diagnostic tests (laboratory or X-ray) and non-adherence to practice guidelines. Child mortality is therefore higher than it should be. Another consequence has been the over-prescription of antibiotics resulting in an increase in drug resistance. Emergence of drug resistance is one of the major public health threats worldwide. Approaches to improve management of infections have largely relied on improving the detection of causative pathogens. However, new approaches based on host biomarkers have emerged to help distinguishing between 1) severe and mild infections and 2) invasive disease and mere colonization, regardless of the microorganism involved. Unlike usual clinical signs, whose diagnostic inaccuracy has been largely demonstrated, these objective measurements will facilitate clinician's assessment and hence appropriate management of patients. High costs from incremental pathogen testing are also avoided, which makes such a strategy potentially attractive for implementation at peripheral health facility and community level.
**Study Objectives**	**General Objective**<br>The goal is to improve the health outcome of children with acute fever through rapid and accurate identification of those children at increased risk of life-threatening infections and those children that would most likely benefit from antibiotics, through the use of point-of-care technologies appropriate for implementation at primary care level. Through this approach we also aim at a more rational use of antimicrobials, and therefore mitigation of the development of antimicrobial resistance and drug side effects, as well as reduction in of misallocation of scarce health resources that results from inappropriate antibiotic use, inappropriate referral to higher level of care, and unnecessary inpatient treatment<br><br>**Specific Objectives**<br><br>**Primary Objective**<br>To compare the clinical outcome of febrile children 2-59 months of age managed using e-POCT (intervention arm), ALMANACH (reference control arm) and routine practice (routine control arm).<br><br>**Secondary Objectives**<br>To compare the rational use of antimicrobials in treating febrile children using e-POCT, ALMANACH and routine care.<br>To compare the performance of e-POCT, ALMANACH and routine care in identifying children at risk for life-threatening infection among febrile children.

	To assess the diagnostic performance of new-generation host-biomarkers in identifying children at risk for life-threatening infection and for clinical failure among febrile children (e-POCT and ALMANACH arms).<br>To measure the proportion of febrile children with hypoxemia, stratified by diagnostic classification (e-POCT arm).<br>To assess the diagnostic performance of new generation host biomarkers in identifying children in need for antibiotics among febrile children (e-POCT and ALMANACH arms).
**Primary Endpoint**	The primary outcome measure is the proportion of clinical failure by day 7 compared among the 3 study arms.
**Secondary Endpoints**	**Secondary outcome measures** are as follows:<br>• proportions of secondary hospitalization and death by day 30 compared among the 3 study arms.<br>• proportions of children prescribed an antibiotic and/or antimalarial treatment at day 0 and by day 7 compared among the 3 study arms.<br>• proportions of children with hypoxemia, stratified by diagnostic classification (e-POCT arm)<br>• performance of combinations of host biomarkers in identifying children at risk for life-threatening infections and for clinical failure among children presenting with fever (e-POCT and ALMANACH arms).<br>• proportion of primarily admitted children compared among the 3 study arms.
**Exploratory Endpoints**	Performance of combinations of host biomarkers in identifying children in need for antibiotic treatment, by type of infection (ALMANACH arm).<br>Accuracy of a new automated respiratory rate device in measuring respiratory rate in children with fever compared to gold standard (manual count).
**Study design**	Randomized controlled non-inferiority trial investigating the e-POCT tool among children 2-59 months of age with fever. Children will be enrolled from outpatient departments of selected health facilities in Dar es Salaam, Tanzania. Study participants will be randomly assigned to be managed by e-POCT (Arm 1, intervention) or by ALMANACH (Arm 2, reference control). Randomization will be at patient level. In parallel, a smaller number of children will be enrolled and managed according to routine care (Arm 3, routine control). We chose to include a routine care arm in order to have a reference point in case of an unusual epidemic that would occur during the year of the study.<br>All children (enrolment arms 1-3) will be evaluated at the time of enrolment (day 0) and have follow-up evaluations on day 3 and 7 (and 14 if not cured at day 7), or at any time in between in case of clinical deterioration according to the caretakers. All patients will also be followed up on day 30 via a phone interview.<br>Patients with unspecific fever and/or severe illness from arm 2 (ALMANACH) will also be included into a sub-cohort study that aims at investigating new host biomarkers profiles according to the type of infection.
**Sample size**	Sample size calculation based on non-inferiority of e-POCT compared to ALMANACH (level of significance 95%, power 80%), assuming 10% clinical failure rates in both groups and a difference between arms of +/-3% (lost of follow up 15%). 1500 children will thus be included in both the ePOCT and ALMANACH arms.<br>For the routine care arm, 500 children will be included to ensure that the distribution of main complaints is not significantly different from the 2 other arms.

<table>
<tr><td>Interim Analyses</td><td>Interim analyses to compare the clinical failure rates by day 7 between arm 1 (intervention) and arm 2 (reference control) will be performed on the first 100 and 500 patients included in each arm. The absolute difference in clinical failure rates should be no more than 5%. The stopping rules are based on an alpha of 0.025 for a one-sided confidence interval. The examples use clinical failure rates of 5% and 10% (based on previous study results) in the control ALMANACH arm.
<table>
<tr><th>number of patients per arms 1 and 2</th><th>number of failures expected in ALMANACH arm</th><th>number of failures in e-POCT arm if 5% worse than ALMANACH (acceptable)</th><th>upper CI bound for 5% more failures: STOP if more than this number</th></tr>
<tr><td colspan="4">ALMANACH failure rate = 5%, e-POCT not worse than 10%</td></tr>
<tr><td>100</td><td>5</td><td>10</td><td>40</td></tr>
<tr><td>500</td><td>25</td><td>50</td><td>81</td></tr>
<tr><td colspan="4">ALMANACH failure rate= 10%, e-POCT not worse than 15%</td></tr>
<tr><td>100</td><td>10</td><td>15</td><td>52</td></tr>
<tr><td>500</td><td>50</td><td>75</td><td>114</td></tr>
</table>
</td></tr>
<tr><td>Study Duration</td><td>Inclusion of patients up to the targeted sample size.</td></tr>
<tr><td>Schedule</td><td>Preparation of the study: 6 months (starting March 2014)
Inclusion of patients: ca 16 months
Data analysis and writing: 12 months</td></tr>
<tr><td>Statistical Analyses</td><td>
Difference between the proportions of children with clinical failure by day 7 in the 3 study arms.
Difference between the proportions of children with secondary hospitalizations and who died by day 30 in the 3 study arms.
Risk ratio of the proportions of children prescribed antibiotics and antimalarials at day 0 and by day 7 in the 3 study arms.
Diagnostic performance of 1) individual host biomarker(s) based on crude positive and negative likelihood ratios, and on the area under the curve of receiver operator characteristic (ROC) curves, and 2) combined biomarkers based on sensitivity and specificity generated by classification and regression trees (CART), to predict presence and development of a life-threatening disease, clinical failure, or an infection requiring antibiotic treatment.
Risk ratio of the proportions of children primarily admitted in the 3 study arms.
</td></tr>
</table>

2 BACKGROUND INFORMATION AND RATIONALE, PRELIMINARY DATA

2.1 Infections are the main cause of under-five mortality

Infections are the major cause of under-five mortality worldwide: in 2010, 64% of deaths were attributed to infectious diseases with pneumonia, malaria, and sepsis as main causes, particularly in Africa[1]. Under five mortality rate in Tanzania has decreased in last decade but remains high: it was estimated at 81 per1000 live births during the period of 2007 to 2010[2]. Infections are the leading cause for paediatric outpatient consultations in Africa. In a recent Tanzanian study 84% of children under five attending a public outpatient facility in Dar es Salaam complained of fever [3]. The key task facing clinicians in these settings is distinguishing children who are more likely to have either serious infection (e.g., meningitis, pneumonia), or complications of infection (eg, dehydration from viral gastroenteritis, hypoxemia from viral bronchiolitis) from the vast majority of children with self-limiting or minor infections who can safely be managed on an ambulatory basis.

2.2 Shortcomings of current clinical algorithms for the management of fever in children

An early and accurate diagnosis of serious infection in children is essential to reduce morbidity and mortality. There is currently no adequate clinical tool for the management of fever in children in resource-poor settings. Children are managed according to symptom-based clinical guidelines such as the WHO Integrated Management of Childhood Illness (IMCI) guidelines. The IMCI treatment algorithms was developed in the 1990's and consist of an evidence-based approach to the assessment, classification and treatment of the most common causes of childhood mortality. When used effectively, its positive impact on mortality could be demonstrated in one study in Tanzania conducted from 1997 to 2002 as well two recently published studies conducted in Egypt between 2000 and 2006 and in India between 2007 and 2010.[4]. Also, IMCI's positive impact on quality of care was shown in Tanzania [5] and other places[6]. These studies concluded that IMCI has the potential to improve quality of care, and to reduce under-five mortality and cost treatment costs, and reduce—when implemented correctly.

However, the actual impact of IMCI worldwide has been less than anticipated due to limited uptake of the intervention, especially among the world's most poor[7]. There are several reasons for this including the following:

- In Tanzania and elsewhere health worker adherence to algorithms is uneven and often quite low, both in rural and urban settings[5],[8].
- Where health workers are implementing IMCI, assessments are frequently incomplete, and children requiring urgent referral are missed. For instance, in an observational study among 77 IMCI-trained health care workers only 12% of observed health workers checked general danger signs in every child, and only 18% assessed all the main symptoms in every child. Only 46.8% of children with severe classifications were correctly identified [9].
- The cost and time for training in the use of algorithms (11-16 days of initial provider training is required) has limited their uptake worldwide. Further, revision of algorithms in response to changing disease patterns and drug resistance has also been limited by these resource-constraints: retraining and reprinting of all materials are costly. Consequently outdated algorithms are often used. To face this challenge efforts have been made to provide countries with electronic versions of the guidelinesthat can be adapted to local needs and updated in real-time. However,retraining of health workers on each new version remains an issue.
- The current practice of IMCI algorithms requires health workers to follow flow diagrams, understand and use accessory information on each page, and accurately flip through up to 8-10 pages of the chart book. In actual practice, most health workers decide not to use the charts due to the embarrassment of using a chart booklet in front of patients.
- IMCI guidelines lack specificity in their recommendation for antibiotic prescription in some diagnostic classifications, due to the lack of field-applicable diagnostic tools. For example for pneumonia, current IMCI guidelines recommend antibiotic prescription to all children presenting with "cough or difficult breathing" and "fast breathing" leading to overtreatment

with antibiotics[10]. In contrast, clinical signs that could point to the need for referral to a higher level of care for parenteral antibiotic treatment or supportive measures such as oxygen therapy are currently not included in IMCI guidelines[11].

- Bacterial infections such as typhoid fever or urinary tract infection are not covered by the IMCI algorithm. These infections are an important cause of fever in children. Even if their actual contribution to morbidity and mortality is not well known, negative outcomes of these infections are feared by health care workers (Rambaud-Althaus C., *personal communication*). They are a major motivation for clinicians to over-prescribe antibiotics.

A careful revision the IMCI algorithm and its overall strategy for management of childhood infections is urgently needed to improve adherence, quality of care and ultimately health outcomes.

2.3 Changes in disease patterns and drug resistance: decrease in malaria prevalence, increase in bacterial drug resistance

During the past 20 years since the first IMCI protocols were developed, patterns of disease and drug resistance has changed dramatically. As for malaria, global disease prevalence has been reduced substantially during the last decade[12,13], secondary to rapid scaling-up of effective preventive measures. Given this reduction in malaria prevalence, the traditional treatment approach, which consisted in presumptive treatment on the basis of symptoms alone, has led to an enormous wastage of medicines and accelerated emergence of drug resistance[14]. A series of studies by our group showed that treatment for malaria based on laboratory confirmation by RDT was safe: we demonstrated that management on the basis of a malaria RDT result of travellers in Switzerland and of children in Tanzania and Papua New Guinea was equivalently safe compared to microscopy result and presumptive treatment, respectively [15-17]. This pivotal work, as well as the result of a systematic review on the true proportion of malaria among fever episodes in Africa,[18] triggered the switch of the WHO malaria case management policy from presumptive treatment to treatment based on laboratory confirmation, usually with RDT[19]. Laboratory testing for all suspected malaria cases is required henceforward as underlined by the WHO policy on malaria case management[20]. Implementation of malaria RDTs at large scale now allows targeted treatment of only those malaria cases confirmed by diagnostic test--rather than presumptive treatment based on clinical symptoms alone. Where RDTs for malaria are available, antimalarial drugs are used more rationally, potentially decelerating the development of drug resistance[3]. The flipside of this positive development in malaria management has been a substantial increase in prescription of antibiotics: fever episodes that were thought to be malaria are now presumed to be bacterial infections--though the majority of children suffer from viral infections and do not require antibiotic treatment (see Section 2.4). For example, after malaria RDT implementation in 2008 in Dar es Salaam, Tanzania, 87% of febrile children under the age of five were prescribed an antibiotic [3]. The same phenomenon has been observed in Uganda[21]. Adequate clinical tools for management of febrile episodes that are not caused by malaria ('non-malaria fevers') that would give guidance to clinicians for prescription of antibiotic treatment, are indeed lacking [22].

As a result antibiotic resistance has increased dramatically and is a particular problem in low resource settings [23-24]. In Tanzania, there is recent evidence that common bacteria are multidrug resistant to many first-line antibiotics. For example, 68% of pneumococcal isolated in the nose and throat of healthy children attending the child health clinic at Muhimbili hospital, Dar es Salaam, had decreased susceptibility to penicillin[25]. Bacteria of all types isolated by blood culture from 2005 to 2009 in the same hospital showed high resistance to penicillin G (71%), tetracycline (64%), cefotaxime (63%) and ampicillin (62%). Moderate to high resistance was seen against chloramphenicol (45%), erythromycin (35%), ciprofloxacin (29%), co-trimoxazole (25%) and gentamicin (24%). Of *Staphylococcus aureus* isolates, 23% were resistant to methicillin[26]. In another study conducted in 2004-05 in Mwanza, Tanzania, all *Shigella* strains isolated in 2007 from diarrheal patients showed high level of resistance to ampicillin, tetracycline, co-trimoxazole and chloramphenicol; 5% and 2% were also resistant to amoxicillin-clavulanate and azithromycin but, at that time, all were still sensitive to ciprofloxacin[27]. Although the correlation between reduced antibiotic susceptibility in vitro and clinical response to treatment is not straightforward, data from Tanzania

shows that bacterial resistance has indeed clinical implications. Children infected with resistant microorganisms were more likely to die[28]. Although it is known that the inappropriate use of antibiotics by health workers has led to widespread drug resistance, multiple approaches used to reverse this trend have been largely ineffective. In a systematic review of the effectiveness of all possible intervention to promote rational use of antibiotics in developing countries, educational interventions successfully improved targeted antibiotic prescribing outcomes by only 20% and even these changes were not necessarily sustainable over time[29]. A holistic strategy is needed to contain antibiotic resistance. Improvement of clinician adherence to evidence-based guidelines is one proposed component.[30] Additional new strategies are urgently needed to have a chance to contain antibiotic resistance at global level.

2.4 Less febrile episodes caused by malaria: what are the causes of 'non-malaria fevers'?

To investigate causes of 'non-malaria fevers' our research group conducted the first comprehensive aetiology study of febrile episodes in African children: 1005 children <10 years (94% less than 5 years) presenting with fever were enrolled in two outpatient clinics in Tanzania [31]. A detailed medical history was taken and clinical examinations were done in a standardized way. Blood and naso-oropharyngeal samples were taken to perform rapid tests, serologies and blood culture as well as a wide range of molecular analyses for pathogens causing acute fever. Urine and stool analyses and CXR were done for specific subgroups of children. To ensure consistency, all investigations were performed according to a pre-defined algorithms and the final diagnosis was computer-generated. Based on the clinical presentation supplemented by 25’743 laboratory and radiological investigations, 1232 individual diagnoses were established, with 22.6% of the cases having more than one diagnosis. Overall, 62% of the cases had an ARI, of which 5% only had radiological pneumonia, 13% had a systemic bacterial, viral or parasitological infection (other than malaria and typhoid), 12% another viral infection isolated in the nasopharynx, 11% had malaria, 10% gastroenteritis, 6% urinary tract infection, 4% typhoid fever, 2% skin/mucosal infections and 0.2% meningitis. Only 3% of the cases remained undiagnosed (see

, where numbers are percentages of all diagnoses, not cases).

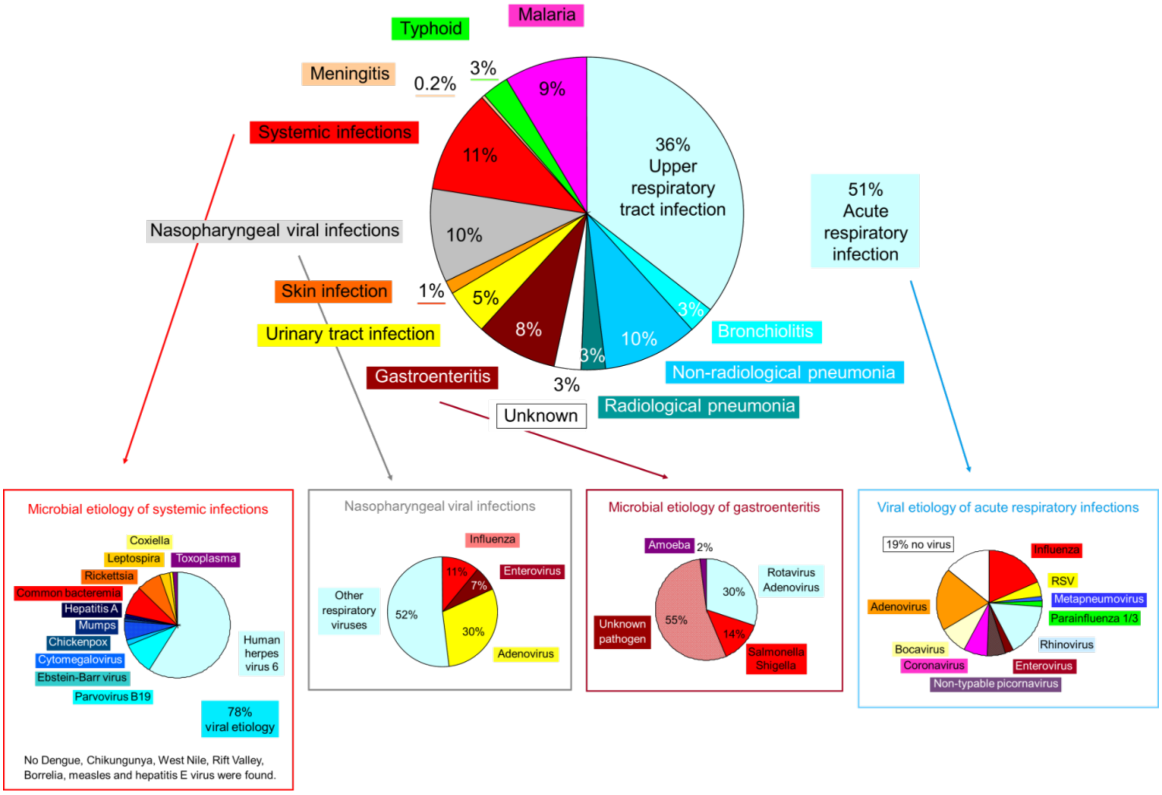

Figure 1: Distribution of all diagnoses (1232) in 1005 febrile children in two sites in Tanzania (numbers are percentages of all diagnoses, not cases)

Among the subgroup of 133 severe cases (based on WHO criteria) 51% had an ARI (of which 13% were radiological pneumonia), 28% malaria, 11% gastroenteritis, 11% a systemic infection, 8% typhoid, 5% UTI, 5% a nasopharyngeal viral infection, 2% meningitis and 2% no aetiology. Malaria, pneumonia and typhoid were overrepresented in severe cases compared to mild cases. The 3 most frequent reasons to be classified as severe were jaundice (32% of the children), respiratory distress (28%) and impaired consciousness (20%).

Diseases by type of pathogen

The distribution of illnesses by pathogen type(s) (bacterial, viral, parasitic) is presented in Figure 2A. shows the overlap among all pathogens identified, irrespective of the clinical and laboratory criteria used to determine final diagnoses. In total, 71% of the 1005 children had documented viral (the five most frequently identified being respectively influenza, rhinovirus, adenovirus, human herpesvirus 6 and coronavirus), 22% bacterial (urinary tract infections, typhoid fever, cutaneous or mucosal purulent infections, bacterial gastroenteritis and occult bacteraemia) and 11% parasitic (malaria, toxoplasmosis and amoebic gastroenteritis) diseases. None of the children were diagnosed with vector-borne diseases such as Dengue, Chikungunya, West Nile and Rift Valley fever.

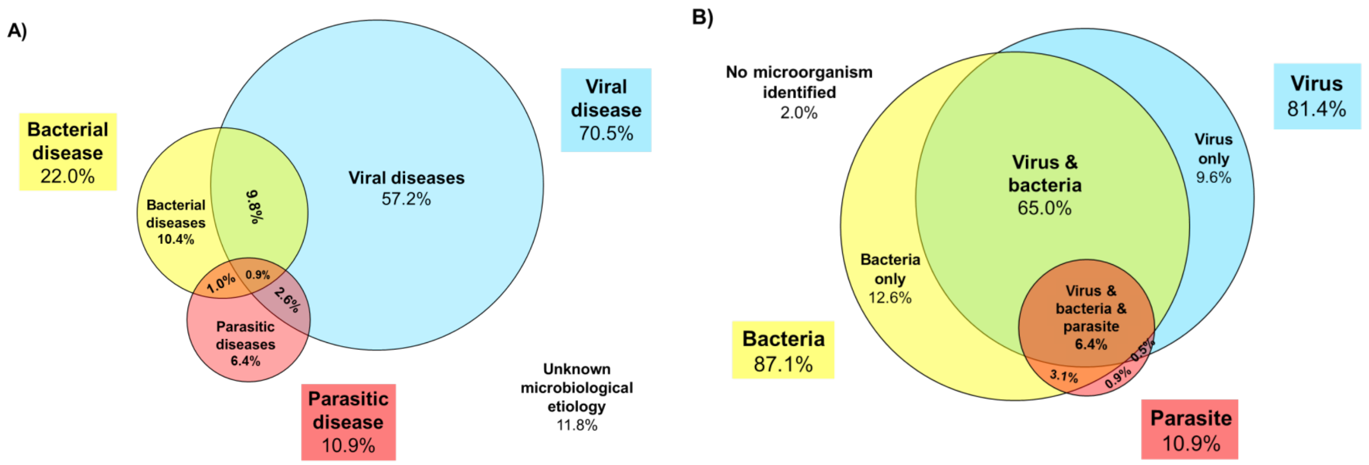

Figure 2: Overlap among viral, bacterial and parasitic diseases* (Figure A) and among all viruses, bacteria and parasites identified# (Figure B) in 1005 febrile children.

* Based on pre-defined case definitions combining clinical findings and laboratory results; # Based on any pathogen-specific positive laboratory test, regardless of case definitions

2.5 The path towards improved fever management algorithms

The difference depicted by the two graphs (**Error! Reference source not found.**) underscores the limitations inherent to a diagnosis made on the basis of etiologic laboratory findings alone. Modern microbiological diagnostic tools, such as PCR, are very sensitive at detecting pathogens (one or more bacteria and/or virus was found in 87% and 81% of the children respectively). However these methods are not able to differentiate carriage from infection (asymptomatic or incidental), infection from disease (symptoms or signs are present), or mild from severe disease. This over-detection leads to treatment of incidentally found pathogens and hence increases drug pressure and antimicrobial resistance. It is thus essential to combine laboratory findings with detailed clinical information. In addition the use of measurements of host biomarkers of inflammation or related to other pathways may allow to improve diagnostic accuracy and predict disease severity.

2.5.1 ALMANACH: electronic clinical algorithms integrating disease etiology with clinical parameters

The findings (epidemiology and predictors of diseases) of the 'Fever Study' were then integrated in a new clinical decision chart (ALMANACH)[32]. The ALMANACH algorithm is displayed in Figure 3. ALMANACH's safety was first assessed in a controlled clinical study (Pan African Clinical Trials Registry PACTR201011000262218)comparing the new algorithm to routine IMCI-based management in Tanzania.[33] 15% in the ALMANACH arm and 39% in the control arm were diagnosed as having an infection requiring antibiotic treatment, while 4% and 10%, respectively, had malaria (based on RDT). 97.3% (95%CI 96.1-98.0%) were cured at day 7 using ALMANACH versus 92.0% (95%CI 89.8-94.1%) using standard practice ($p<0.001$). Of 23 children not cured at day 7 using ALMANACH, 44% had skin problems, 30% pneumonia, 26% upper respiratory tract infection and 13% likely viral infection at day 0. Secondary hospitalization occurred for one child with ALMANACH and one child who eventually died using routine practice. At day 0, antibiotics were prescribed to 15% using ALMANACH versus 84% using routine practice ($p<0.001$). 2.3% versus 3.2% received an antibiotic after day 0. The conclusion was that management of children using ALMANACH improved clinical outcome and reduced antibiotic prescription by 80%. This achievement was made thanks to more accurate diagnoses, and hence better identification of children in need of antibiotic treatment or not. ALMANACH was then programmed into smartphones and electronic tablets to improve adherence of clinicians to the algorithm (SNF Joint Partnership n°124023, PI B. Genton, co-PIs V. D'Acremont& J. Kahama-Maro) (Figure 4). Electronic devices allowed automated generation of monthly reports and feed-back to health providers and district health authorities (Figure 5).

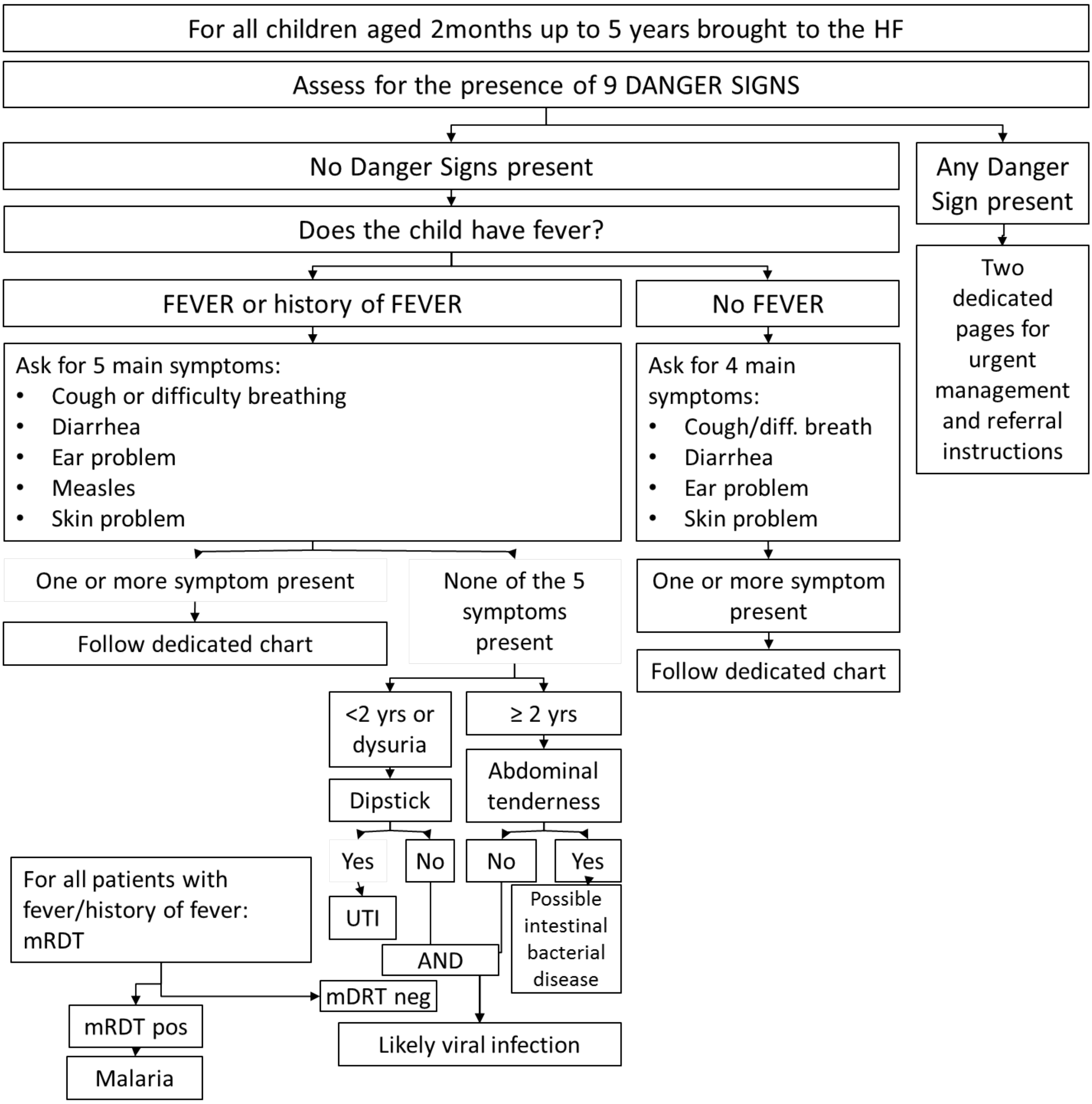

Figure 3: ALMANACH algorithm

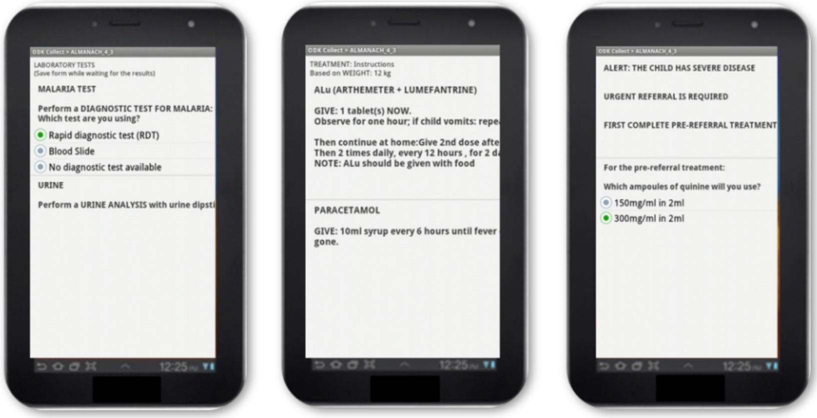

Figure 4: Interactive algorithm built in an electronic support (android application for smartphones and tablets using Open Data Kit Collect and OpenMRS).

PeDiAtrick project

ALMANACH Report 2012 TABLES

Dar es Salaam
Temeke district
KIZUIANI Dispensary

Diagnoses reported by clinicians

KIZUIANI 2012

Diagnoses	April	May	Jun	Jul	Aug	Sept
Malaria	135	83	36	20	25	31
URTI	102	186	105	31	48	51
Diarrhoeal diseases	102	137	85	36	32	50
Intestinal worms	0	2	1	0	0	0
Pneumonia	27	44	18	3	1	10
Eye infections	3	1	2	0	0	0
Ear infections	21	16	7	2	2	7
Skin infections	23	37	28	9	7	16
Asthma	0	0	0	0	0	0
UTI	28	30	11	7	7	7
Dysentery	0	7	5	0	3	4
Intestinal bact. Disease	0	25	5	1	1	14
Anemia	6	7	3	0	0	2
Malnutrition	0	0	0	0	0	0
Measles	4	4	0	1	0	2
Likely viral infection	28	82	64	29	21	35
Other diagnoses	11	17	15	2	2	3
TOTAL	490	678	385	141	149	232
Severe disease			24	11	6	10

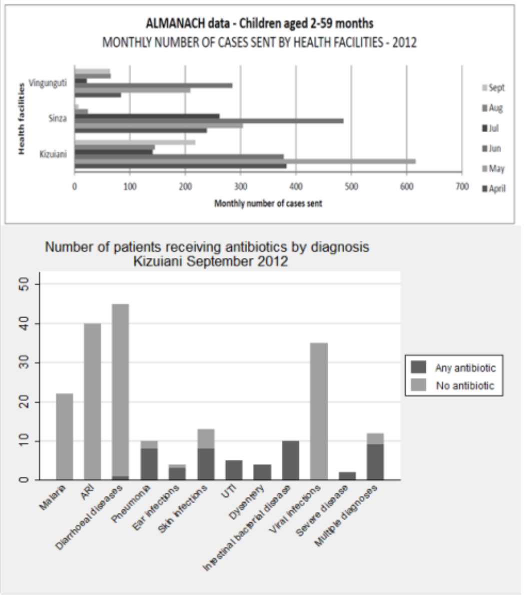

Figure 5: Different types of reports generated in real time through the implementation of ALMANACH on mobile phones and android tablets

Despite these enhancements under controlled study conditions, the uptake of the tool in routine practice was suboptimal: completing the entire algorithm was too time consuming and clinicians were unable to identify key signs such as tachypnea [34].

2.5.2 The next steps: rationale for development of e-POCT

2.5.2.1 How well do clinical parameters identify children with serious infections? The current evidence

One of the key tasks in outpatient settings, both in the developing and developed world, is to distinguish children who may have serious infections or complications of infections (e.g. meningitis, bacteraemia, hypoxia from bronchiolitis, dehydration from gastroenteritis) from the vast majority with self-limiting or minor infections who can safely be managed as outpatients or without antibiotics. To differentiate children who have a benign self-limiting viral infection from the small proportion with serious bacterial infections, several triage and management tools such as Emergency Severity Index,

the Paediatric Canadian Triage and Acuity Scale, Paediatric Risk of Admission Score, and the Paediatric Emergency Assessment Tool and The National Institute for Health and Clinical Excellence (NICE) guideline for the management of feverish illness in children under 5 years of age [35–40]. These guidelines are based on expert review as well as a series of primary studies on clinical/ laboratory prediction models [41–47]. All of these studies were performed in developed countries besides one study that assessed clinical predictors for severe infection in children under 2 months of age, an age group that will not be included in our study[48].

A large number of clinical predictors were studied in these primary research studies but no common set of predictors for identification of serious bacterial infections could be identified. Many of the identified predictors lack external validity. This is not surprising since a uniform pathophysiological disturbance in all serious infections is unlikely. Moreover, given the large number of potential predictors and the relative low prevalence of serious infections, data- driven analytic approaches may have resulted in a number of predictors identified by chance but miss true predictors. This may have caused limited external validity of previous studies [49]. For example, Craig et al. assessed the accuracy of clinical symptoms and signs for the diagnosis of serious bacterial infections - that is, pneumonia, urinary tract infection, and bacteraemia - in 15'781 children under the age of five presenting with fever[45]. Based on 40 clinical symptoms and signs a final model based on 26 items was developed. The performance of the final diagnostic models for each infection was acceptable, with all area under the curve calculations between 0.8 and 0.9. However, the large number of clinical variables which limits the feasibility of using the model in resource-poor settings. One other major finding of this study was that that clinical signs and symptoms contribute differently to predicting the risk of particular serious bacterial infections.

Thompson et el. evaluated the accuracy of a much small set of objective signs - that is routine vital signs obtained at emergency room triage - in identifying children with infections requiring antibiotic therapy or hospital admission. Capillary refill time had the highest positive LR, whereas all other vital signs had similar negative LR in the 0.7-0.9 range (Table 1).

	Sensitivity (95% CI)	Specificity (95% CI)	Positive LR (95% CI)	Negative LR (95% CI)
Temperature ≥39.0°C	27 (22 to 32)	87 (84 to 91)	2.1 (1.5 to 2.9)	0.8 (0.8 to 0.9)
Tachypnoea	55 (49 to 61)	57 (51 to 62)	1.3 (1.1 to 1.5)	0.8 (0.7 to 0.9)
Tachycardia	62 (57 to 68)	58 (53 to 63)	1.5 (1.3 to 1.7)	0.7 (0.6 to 0.8)
CRT >2 seconds	8 (4 to 12)	100 (99 to 100)	17.7 (2.4 to 132.5)	0.9 (0.9 to 1.0)
O_2 sats ≤94%	19 (15 to 24)	93 (90 to 95)	2.7 (1.7 to 4.1)	0.9 (0.8 to 0.9)

CRT, capillary refill time; LR, likelihood ratio; O_2 sats, oxygen saturations.

Table 1: Diagnostic characteristics of individual vital signs for identifying children with serious (conditions that were likely to be life-threatening if untreated or with high chance of life-threatening complications or sequelae, n=108) or intermediate (conditions that were not likely to be life-threatening, but were expected to last for longer than 10 days or have a non life-threatening complication, n=205) infection vs those with minor (conditions from which the child was expected to recover without sequelae, n=339) /no infection (n=48).[50]

In a systematic review by Thompson et al. the diagnostic performance of clinical features in identifying serious infection in children in developed countries that were assessed in individual studies up to 2010 [44]. The major findings are summarized in Figure 6 and Figure 7 which again underscores the heterogeneity of predictors. The strongest red flags for serious infection identified in this systematic review were reduced consciousness, convulsions, cyanosis, rapid breathing, and slow capillary refill.

	Study reference	Prevalence*	Age range	Likelihood ratio (95% CI)	
				Positive	Negative
Cyanosis	5	Low	<17 years	52·20 (10·50–258)	0·93 (0·85–1·03)
	33	High	3 months to 16 years	2·66 (1·73–4·10)	0·87 (0·82–0·93)
	45†	High	1 month to 15 years	50·20 (2·97–846)	0·88 (0·81–0·95)
Poor peripheral circulation	5‡	Low	<17 years	38·80 (11·20–134)	0·90 (0·80–1·02)
	42§	Intermediate	1 month to 5 years	4·71 (2·07–10·7)	0·52 (0·29–0·94)
	42§¶	Intermediate	1 month to 5 years	10·50 (5·00–22·1)	0·04 (0·03–0·60)
	33	High	3 months to 16 years	17·70 (2·36–132)	0·92 (0·88–0·96)
	41‡§	High	1 month to 5 years	11·70 (4·78–28·7)	0·55 (0·43–0·69)
	45†	High	1 month to 15 years	3·71 (2·32–5·93)	0·56 (0·44–0·73)
	24	High	1 –36 months	2·39 (1·50–3·82)	0·83 (0·73–0·94)
Crackles	5	Low	<17 years	6·00 (3·52–10·10)	0·72 (0·56–0·91)
	46||	Intermediate	2–59 months	1·51 (0·81–2·83)	0·92 (0·79–1·07)
Decreased breathing sounds	5	Low	<17 years	9·30 (4·42–19·70)	0·82 (0·68–0·98)
	46||	Intermediate	2–59 months	2·21 (0·89–5·50)	0·93 (0·84–1·04)
Short of breath	5	Low	<17 years	9·30 (5·83–14·80)	0·64 (0·48–0·85)
	46||	Intermediate	2–59 months	1·11 (0·70–1·74)	0·96 (0·78–1·18)
	24	High	1–36 months	3·60 (2·06–6·28)	0·81 (0·72–0·91)
Rapid breathing	5	Low	<17 years	9·78 (5·71–16·70)	0·70 (0·55–0·89)
	47||	Intermediate	<2 years	3·08 (2·41–3·94)	0·37 (0·23–0·60)
	33	High	3 months to 16 years	1·26 (1·07–1·49)	0·80 (0·68–0·94)

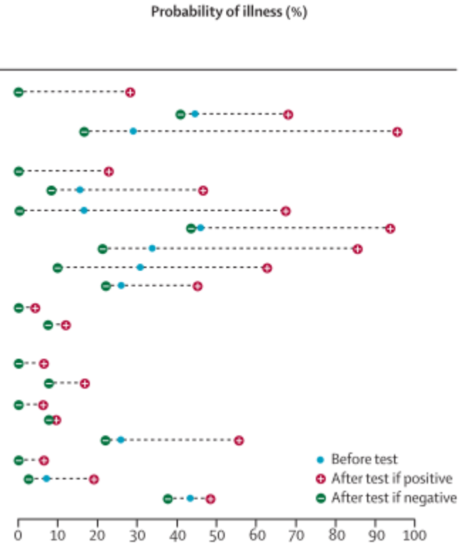

Figure 6: Potential warning signs for serious illness (positive likelihood ratio >5·0 in at least one study)—circulatory and respiratory features *Setting: low prevalence of serious infection (<5%); intermediate prevalence of serious infection (5–20%); high prevalence of serious infection (>20%). †Meningitis only. ‡Capillary refill more than 2 s. §Gastroenteritis causing dehydration only. ¶Digitally measured capillary refill. ||Pneumonia only. From [44]

	Study reference	Prevalence*	Age range	Likelihood ratio (95% CI)	
				Positive	Negative
Meningeal irritation	5	Low	<17 years	25·70 (3·09–213)	0·97 (0·91–1·03)
	44†	Intermediate	3 months to 6 years	275 (16·70–4526)	0·52 (0·35–0·76)
	48‡	Intermediate	>1 month to 16 years	13·90 (5·41–35·60)	0·61 (0·47–0·79)
	45†	High	1 month to 15 years	2·57 (2·16–3·06)	0·01 (0·00–0·15)
Petechial rash	5	Low	<17 years	12·50 (1·65–94·9)	0·97 (0·91–1·03)
	44†	Intermediate	3 months to 6 years	83·70 (4·50–1475)	0·86 (0·73–1·01)
	48‡§	Intermediate	>1 month to 6 years	9·00 (5·26–15·3)	0·28 (0·16–0·48)
	49‡§	Intermediate	≤15 years	7·00 (4·60–10·70)	0·19 (0·08–0·46)
	45†	High	1 month to 15 years	6·18 (2·68–14·30)	0·81 (0·73–0·91)
	32	Intermediate	1–36 months	8·90 (2·63–30·40)	0·75 (0·63–0·91)
Seizures	5	Low	<17 years	20·70 (4·83–88·60)	0·94 (0·86–1·03)
	43†¶	Intermediate	6 months to 6 years	5·90 (1·79–19·00)	0·80 (0·59–1·08)
	44†¶	Intermediate	3 months to 6 years	3·50 (1·69–7·17)	0·76 (0·58–1·00)
	45†	High	1 month to 15 years	1·68 (0·66–4·27)	0·96 (0·90–1·04)
Unconsciousness	5	Low	<17 years	19·80 (6·17–63·50)	0·91 (0·81–1·02)
	44†	Intermediate	3 months to 6 years	155 (9·03–2677)	0·73 (0·57–0·93)
Decreased skin elasticity	41||	High	1 month to 5 years	10·70 (3·87–29·8)	0·67 (0·56–0·81)
Hypotension**	49‡	Intermediate	≤15 years	9·40 (1·99–44·70)	0·74 (0·56–0·99)
Any abnormal finding in history or physical examination	29	Intermediate	<24 months	4·42 (2·87–6·80)	0·18 (0·71–0·44)

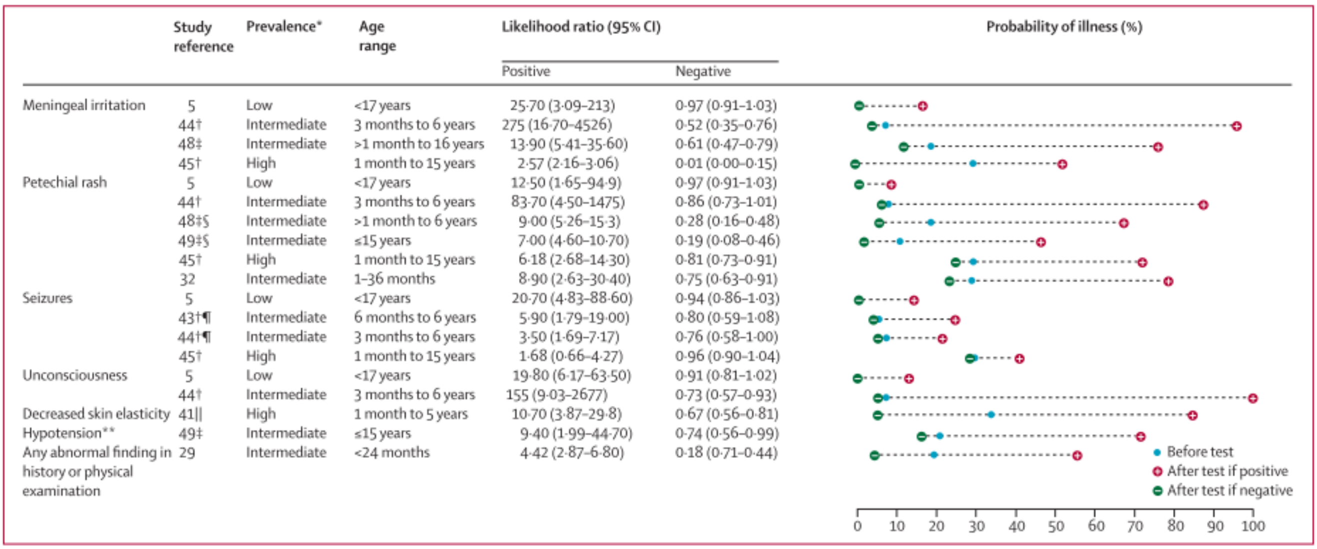

Figure 7: Potential warning signs for serious illness (positive likelihood ratio >5·0 in at least one study)—miscellaneous *Setting: low prevalence of serious infection (<5%); intermediate prevalence of serious infection (5–20%); high prevalence of serious infection (>20%). †Meningitis only. ‡Meningococcal infection. §Diameter more than 2 mm. ¶During examination. ||Gastroenteritis causing dehydration only. **Hypotension defined as 2 SD or more below the mean for age. From: [44]

None of the above studies have been validate in resource-poor settings. In resource-poor settings IMCI was introduced in the 1990s. IMCI relies on several clinical signs (history or clinical exam) to detect need for antibiotic management and referral to higher level of care. These signs were taken from disease-specific algorithms and combined by WHO experts. Aside from clinical predictors for pneumonia most included predictors were chosen based on expert clinical opinion [51].

General danger signs	Pneumonia	Dehydration	Fever
not able to drink or breastfeed	Respiratory rate over one minute	Sunken eyes	Duration of fever
vomits everything	Chest indrawing	Skin turgor	Stiff neck
History of convulsions	Stridor	Blood in stool	Runny nose
Lethargy/ unconciousness		diarrhea	Signs of measles

Table 2: clinical criteria that are assessed for febrile children in the IMCI algorithm.

Clinical signs were assessed in a series of studies shortly after IMCI introduction [51,52]. For example, Table 3 and Table 4 show the performance characteristics of IMCI in 440 Gambian children aged 2 month to 5 years when compared to gold standard based on clinical evaluation by a paediatrician, CXR and hemoglobin measurement [52].

	No. positive by fieldworker/ No. positive by physician	No. negative by fieldworker/ No. negative by physician	Sensitivity (%)	Specificity (%)
Pneumonia	67/83	318/357	81	89
Dehydration	8/12	413/428	67	96
Parasitaemia	27/31	32/409	87	8
Malaria	17/17	36/423	100	9
Measles	4/4	432/436	100	99
Otitis	16/53	374/387	30	97
Malnutrition	111/124	285/316	89	90
Referral or admission	36/79	335/361	45	93

Table 3: Numbers of children with a particular diagnosis made by a physician and by a field worker according to the IMCI algorithm. Sensitivities and specificities are calculated using the physician's diagnosis made with the help of laboratory investigations as "gold" standard. [52].

Referred by *both* algorithm *and* the physician:		Referred by algorithm but *not* by the physician:		*Not* referred by algorithm but admitted by the physician:	
Diagnosis	*n*	Diagnosis	*n*	Diagnosis	*n*
Pneumonia	18	URTI[a]	8	Pneumonia	19
Malnutrition	5	Gastroenteritis	3	Gastroenteritis	8
Bronchiolitis	4	Asthma	3	Sepsis/PUO[a]	4
Gastroenteritis	4	Malnutrition	3	Osteomyelitis/ septic arthritis	3
Sepsis	2	Bronchiolitis	2	Cellulitis	3
Osteomyelitis	1	Upper airway obstruction	2	Malaria	3
Malaria	1	Malaria	1	Malnutrition	1
Anaemia	1	Anaemia	1	Intestinal obstruction	1
—		Measles	1	Congenital heart disease	1
—		Normal	1	—	
Total	36	Total	25	Total	43

[a] URTI: upper respiratory tract infection. PUO: pyrexia of unknown origin.

Table 4: Primary diagnosis of children referred by algorithm and/or by the physician [52]

For pneumonia a series of studies was undertaken in developing countries that examined predictive accuracy of clinical signs for pneumonia: these included a history of cough or breathlessness, inability to feed, raised respiratory rate, lower chest wall indrawing, fever, and tachycardia [53–57]. These studies have been the foundation for pneumonia detection in developing countries and were later integrated into IMCI[59]. In a series of impact studies it was shown that the pneumonia algorithm was very sensitive in identifying children with pneumonia but lacked negative predictive value resulting in overtreatment with antibiotics [60]. A major shortcoming of the WHO clinical pneumonia assessment is that it does not include hypoxemia--a major risk factor for mortality (see Section 2.5.2.2).
Since all more recent studies investigating the accuracy of various clinical and laboratory signs in assessing children with serious infections were performed in the developed world we evaluated the performance of IMCI and ALMANACH in identifying children with bacterial infections in the Tanzanian 'Fever Study' dataset[31]. We applied ALMANACH and IMCI algorithms retrospectively to the children enrolled in the 'Fever Study'. We compared those children who should have received antibiotics based on the etiological diagnosis determined through complex investigations in the 'Fever Study' versus antibiotic treatment based on clinical parameters in the two clinical algorithms (Figure 8). For IMCI, the overlap was poor (12% of total patients). As for ALMANACH overlap is better (18% of total patients) but at the cost of a greater percentage of antibiotic prescriptions.

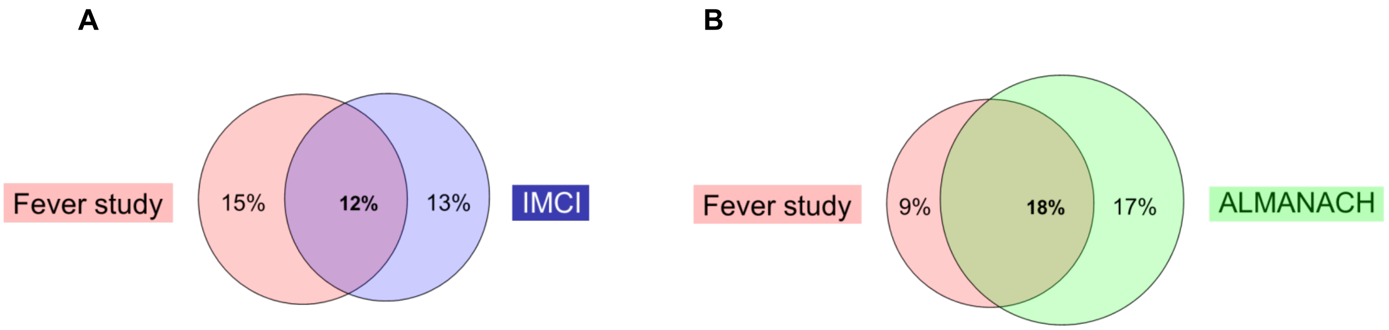

Figure 8: Overlap between the children that should be treated by antibiotic according to the findings of the 'Fever Study' and according to the IMCI algorithm (Figure A) or the ALMANACH algorithm (Figure B) applied to the same children *Source: D'Acremont et al, Meeting on non-malaria fevers, Gates Foundation, Seattle 20 Oct 2011.*

In summary, management algorithms of fever in resource-poor settings built on clinical predictors alone will likely remain unsatisfactory given the limited diagnostic value of a small set of clinical predictors that could be assessed reliably be health care workers. With ALMANACH we demonstrated that rational use of antibiotics can probably not be improved with electronic algorithms using clinical predictors and pathogen-specific POCTs only. ALMANACH was also too complex to be implemented into routine practice in resource-poor settings.

2.5.2.1.1 The way forward: combination of clinical parameters with POCTs

We therefore propose to focus on the added diagnostic value of new variables such as oximetry and other host biomarkers. We believe that the way forward is to create a simplified decision chart including only few critical clinical parameters complemented by few etiologic tests (such as malaria) and host biomarkers POCTs that could identify children at risk for life-threatening infections and those in need for antibiotic treatment (see Section 2.5.3).

2.5.2.2 Oximetry

Hypoxemia is a common and treatable complication of childhood respiratory and non-respiratory infections in developing countries and is associated with a 2- to 5-fold increase in mortality [61].In a review by Subhi et al. the median prevalence of a pooled analysis of hypoxemia (oxygen saturation

≤90%) in WHO-defined severe and very severe pneumonia was 13.3% (IQR 9.3 - 37.5%)[62]. In a Kenyan study, children with hypoxemia were four times more likely to die while children with radiological pneumonia had the same risk of death[63]. No clinical sign can safely replace oximetry and oximeters are now readily available in developing countries. In infants, a respiratory rate ≥70/min, grunting, and retractions were the best clinical predictors of hypoxemia. In older children, a respiratory rate of ≥60/min was the single best predictor, but had still an overall accuracy of only 70%[64]. Moreover, the presence of hypoxemia predicted radiographic pneumonia with a sensitivity and specificity of only 71% and 55%, showing clearly that the need for oxygen goes beyond documented pneumonia. Hypoxemia also complicates acute conditions other than pneumonia: in the same review by Subhi et al., the prevalence was 3-17% in children with malaria, 2-5% in children with diarrhoea and 18%-23% in sick neonates.[62] In a recent cohort of children presenting at peripheral level with febrile illnesses, oximetry was systematically performed.[17] In this cohort, the proportion children that were found to be hypoxemic (oxygen saturation <94%) with severe pneumonia, severe malaria and severe illness due to unspecific fever was 29%, 9% and 3%, respectively. In children with non-severe pneumonia, malaria and unspecific fever, the proportions were 6%, 2.4% and 2.3%, respectively (Nicolas Senn, personal communication). Screening using pulse oximetry with provision of oxygen for hypoxemia is known to improve quality of care and reduce mortality (for pneumonia, by 35%). It is sustainable and affordable in developing countries.[65] Oximetry has traditionally been considered merely as a tool for hospital-level of care and has only recently be looked at as potentially useful for the peripheral level of care: in Bangladesh, 360 children with severe pneumonia, of whom 53% were hypoxemic (oxygen saturation <95%), were assigned randomly to receive either ambulatory or hospital care; 88% of the children were successfully managed in the ambulatory group compared to 96% in the hospital care group.[66] Authors concluded that severe childhood pneumonia (without severe malnutrition) can be successfully managed at ambulatory clinics, except for children who require prolonged oxygen therapy. Oximetry, using a higher oxygen saturation threshold (e.g. 94% rather than 90-92%), could also be used as a triage tool at community level, not necessarily to provide oxygen but to detect children at risk for life-threatening hypoxemia that require urgent referral to a higher level of care.[65]

2.5.2.3 Challenges in accurate measurement of paediatric respiratory rate

Tachypnea is an important clinical sign for detection and triage of pneumonia as demonstrated by several studies in resource-poor settings (**Error! Reference source not found.**) [53-57]. However, in children in developing countries, the assessment of respiratory signs shows marked variation between different pairs of clinicians working in the same paediatric service[68]. The usefulness of respiratory rate measurements under such conditions is hence questionable. Several strategies to improve manual count (such as counting beads) have had limited success in increasing inter-observer reliability. UNICEF is currently evaluating automated respiratory rate devices that would be legible by android-based smartphones[69]. Such devices would thus have a great potential to improve fever management in children.

2.5.2.4 Biomarkers

The term ‘biomarker’ is most often used to refer to a test performed on some body fluid (e.g., blood, urine and cerebrospinal fluid) that provides clinicians with patient information not readily obtainable otherwise using current diagnostic or monitoring modalities [70]. Biomarkers can be used for diagnostic purposes or stratification (i.e. classification of disease severity).

CRP and PCT

CRP is one of the earliest discovered biomarkers used to diagnose infection, so named for its ability to precipitate from serum in the presence of pneumococcal cell wall C-polysaccharide. CRP is an acute-phase reactant found in the blood that is produced by hepatocytes in the setting of infection or tissue injury. CRP production is triggered by cytokines (IL-1, IL-6 and TNF-α) and levels increase within 4–6 h of an inflammatory stimulus. Serum CRP concentration doubles approximately every 8 h from that stimulus and peaks at around 36–50 h. It has a short half-life of 4–7 h [70].

PCT is a precursor for the hormone calcitonin, which is produced in the thyroid to regulate serum calcium concentrations. When the body is challenged with infection, however, significant production of PCT by non-thyroidal tissues occurs throughout the body. Although the exact proximal stimuli that mediate PCT secretion are unknown, evidence suggests that early inflammatory signals such as TNF-α, IL-1β and IL-6 play a role. Elevations in PCT are generally observed before CRP rises and levels peak within a much shorter time frame. Additionally, when the patient responds appropriately to therapy, PCT levels return to normal much quicker than those of CRP [70].

CRP and PCT have emerged as promising markers for the diagnosis of bacterial infections. They are available as POCTs that are implementable at peripheral level[71]. A recent review (that only includes studies from Northern countries) of the diagnostic value of laboratory tests in identifying serious infections in febrile children showed that CRP yielded a positive LR of 3.15 and a negative LR of 0.33 (five studies). To rule in serious infection, cut-off levels of 2 ng/mL for PCT (two studies, positive LR 13.7 and 3.6) and 80 mg/L for CRP (one study, positive LR 8.4) were recommended; lower cut-off values of 0.5 ng/mL for PCT or 20 mg/L for CRP were necessary to rule out serious infection[72]. In 377 Malawian children presenting with meningitis or pneumonia, the best predictors of serious bacterial infection were CRP (AUC 0.81) and PCT (AUC 0.86). The best marker for predicting death was PCT (AUC 0.61).[73] A study from Mozambique found that, in the absence of malaria parasites, PCT (AUC 0.90) and CRP (AUC 0.87) predicted invasive bacterial pneumonia when compared to viral pneumonia in severely ill children. Combination of both markers did not improve diagnostic profile, and none of the two markers could predict mortality.[74] In the Gambia, CRP (AUC 0.83) was better than PCT (AUC 0.73) at identifying children with radiological endpoint pneumonia among those with clinical pneumonia,[75]. We had similar findings in the 'Fever Study' (CRP: AUC 0.86 and PCT: AUC 0.71, see below).

CRP or PCT results have been used to support clinical decision making for the initiation and discontinuation of antibiotic therapy. Randomized controlled trials have demonstrated the feasibility of such a strategy in ARI patient populations and different settings ranging from primary care to emergency departments and hospital wards. In a systematic review and individual patient data meta-analysis of 14 trials, no increased risk for mortality or treatment failure was found when PCT was used to guide initiation, and duration of antibiotic treatment in patients with ARI compared with control patients.[76] In terms of efficacy, a lower rate of treatment failure for patients allocated to the PCT group was found, both in the emergency department setting and in patients with CAP. A consistent reduction of antibiotic use was observed in PCT groups, mainly due to lower prescription rates in primary care setting and lower duration of antibiotic courses in emergency departments. Regarding children, three randomized controlled trials have been published that used PCT-based algorithms to guide antibiotic treatment. The first one, investigating children aged 1-36 months with unknown source of fever attending hospital emergency departments in Canada, found no difference between the intervention (PCT-based algorithm) and control, but there was no enforcement to withhold antibiotics if PCT was under a defined threshold.[77] The second one showed a significant benefit of PCT-based guidelines for managing children with neonatal sepsis.[78] The third one, investigating hospitalized children with CAP in Italy found that PCT guidance (using a threshold of <0.25 ng/mL) allowed a significant reduction in antibiotic prescriptions (86% of children treated with antibiotics in the PCT versus 100% in the control group), exposure to antibiotics (mean 5 versus 11 days), and antibiotic-related adverse events (4% versus 25%).[79] There was no significant difference in recurrence of respiratory symptoms and new antibiotic prescription in the month following enrolment between-groups. To our knowledge, a few studies are on-going which should shed further light on the benefits and harms of PCT use in paediatric populations,[80] but none is conducted in primary care setting in developing countries, and none use more than one biomarker.

New generation of biomarkers

Few studies have looked at the performance of biomarkers other than CRP and PCT [81]. Kevin Kain *et al.* have however recently tested 12 different biomarkers in children with malaria in Uganda. A panel of 3 biomarkers (Ang-2, PCT and sICAM-1), when measured at clinical presentation in children with severe malaria, successfully discriminated (sensitivity 91% and specificity 89%) between children who survived, from children who succumbed to their infection [82].

In the frame of a collaborative grant from Bill & Melinda Gates Foundation with Prof. Kevin Kain, Canada, we assessed the ability of biomarkers to identify children of the ‘Fever Study’ in need of antibiotics. In addition to the conventional ones (CRP and PCT), some promising angiogenic and endothelial-based biomarkers that relate to the underlying pathophysiology of critical illness were tested: angiopoietin-2, angiopoietin-1, von Willebrand Factor (vWF), vWF propeptide, vascular endothelial growth factor (VEGF), solulable Platelet-selectin (sP-selectin), solulable intercellular adhesion molecule-1 (sICAM-1), sEndoglin, serum fms-like tyrosine kinase 1 (sFLT-1), sTie-2, solulable troggering receptor expressed on myeloid cells 1 (sTREM-1), Chitinase-3-like protein 1 (CHI3L1), Interferon gamma-induced protein 10 (IP10), Platelet Factor 4, Interleukin 18-binding protein (IL18 bpa), Factor D, and Angiopoietin-like protein 3 (ANG-like 3).[82,83] After establishing best performing cut-offs, classification and regression trees (CART) were built to achieve optimal test performance in identifying children in need for antibiotics. Four biomarkers - CRP (AUC 0.86), CHI3L1 (AUC 0.80), PCT (AUC 0.71) and sP-selectin (AUC 0.72) - were found to significantly discriminate children with radiological endpoint pneumonia from children with cough and fast breathing but no consolidation on CXR. CART analyses generated a model based on CRP, sP-selectin and sEndoglin yielding a sensitivity of 93% and a specificity of 77%. Of the models including two different biomarkers only, the model that achieved the highest accuracy (sensitivity 87% and specificity 87%) was a combination of CRP and CHI3L1 (Figure 9).

Among children with unspecific fever, five biomarkers – IL18bpa (AUC 0.75), CRP (AUC 0.66), sEndoglin (0.65), PCT (AUC 0.61) and sTie-2 (AUC 0.6) - were found to significantly discriminate children with bacterial disease (of any level of severity) from children with viral illnesses. CART generated a model based on CRP and IL18bpa yielding a sensitivity of 90% but a specificity of only 64%. This is due to the heterogeneity of diseases causing unspecific fever. More detailed analyses showed that the biomarkers profiles are slightly different for each bacterial disease (urinary tract infection, typhoid fever, bacterial gastroenteritis and bacteremia), but that they all have in common that CRP was the biomarker with the best performance (better than PCT), especially to identify serious infections. Interestingly, when children had more than one bacterial disease at a time, the CRP was even higher. In conclusion, CRP was a good predictor of radiological pneumonia (better than PCT, as in the Gambian study)[75] and a combination of CRP and CHI3L1 was able to improve the accuracy further. Regarding children with unspecific fever, CRP was a better biomarker than PCT and had a good sensitivity when combined with IL18bpa. When analyzing each bacterial disease separately, CRP was each time the best predictor of bacterial infection, especially for serious infection. Even if it had limited specificity, it would save three-quarter of antibiotic prescriptions, if used in all children with unspecific fever. In summary, there are numerous candidates of such biomarkers that could potentially be used to identify children at risk for life-threatening infections. However, pediatric data on prediction of clinical outcome is currently missing (besides prediction of death among patients with severe malaria).

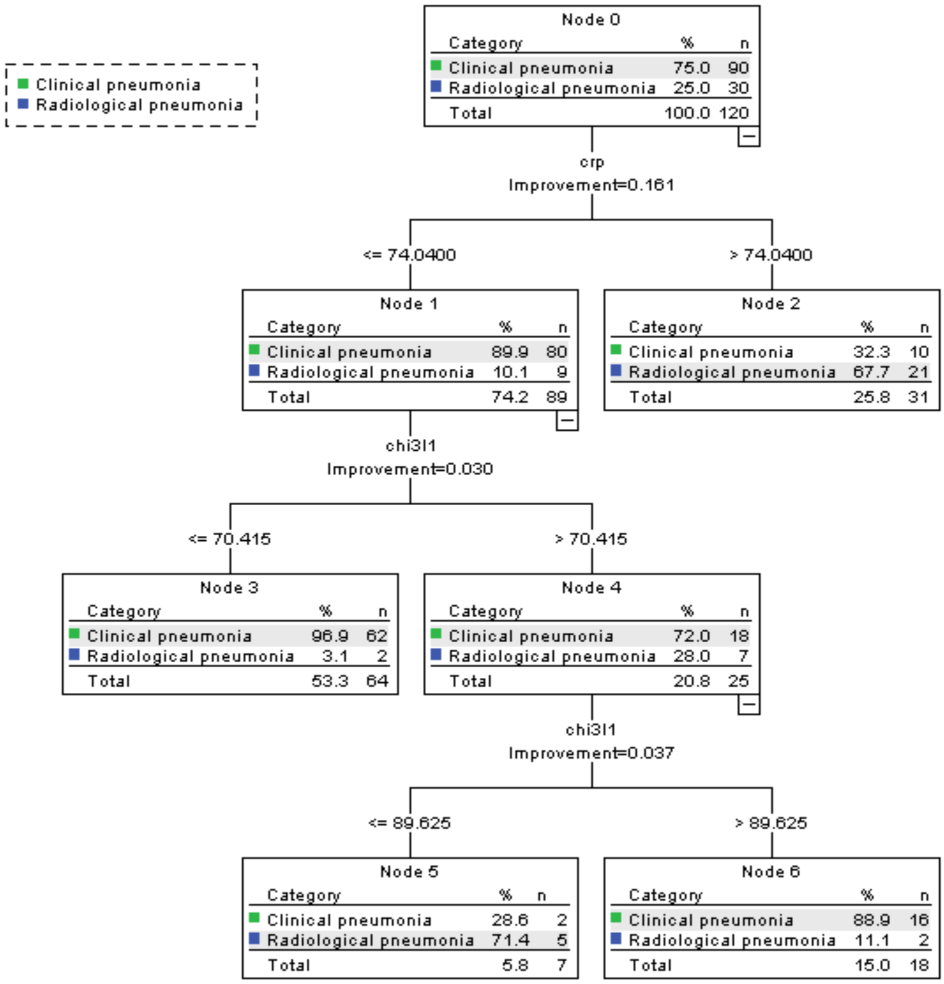

Figure 9: Classification tree analysis to predict radiological pneumonia with host biomarkers

Hemoglobin

Severe anaemia (defined as Hb<5g/dL) is one of the major presentations of severe malaria in children under five and a major contributor to the morbidity and mortality attributable to malaria: in a study among children with malaria in Malawi between 32 and 54% of malaria-related deaths were associated with severe anemia[84]. In a Kenyan study on blood transfusion in hospitalized children, the fatality rate of children admitted with severe anaemia was 18% (125/678) compared with 8% (136/1635) for children with Hb>5.0g/dl[85]. Prompt recognition of severe anaemia at admission is crucial as children benefit most from early transfusions: in the Kenyan study, the probability of mortality from severe anaemia was the same for all children who survived beyond day 1, irrespective of admission Hb or receipt of transfusion. Currently, IMCI guidelines rely on palmar pallor for diagnosis of anemia--a clinical sign that was shown to have poor accuracy for detection of anaemia [86]. Simple filed-applicable hemoglobin testing tools are widely available in resource-poor settings and would be a great asset to fever management tools. In the Tanzanian ‘Fever Study’, anaemia predicted not only malaria but also bacterial infection, with a positive likelihood ratio of 2.0 (95%CI 1.3 - 2.9).Up to now POC Hb measurement has not been integrated into clinical management of children with fever though it can be a valuable tool in identifying children with severe infections. We therefore propose integration of Hb measurement into a novel electronic management (e-POCT). Please refer to Section 2.5.3.

2.5.3 Proposed intervention: e-POCT, integrating clinical parameters with POCTs.

To overcome the above-illustrated shortcomings of current clinical management algorithms for fever we plan on testing a novel clinical management algorithm, e-POCT, that will integrate simple clinical signs with POCTs for selected biomarkers and pathogens (Figure 10). The purpose of this tool is to identify children at risk of complications from infections (e.g. septic shock, dehydration, meningitis) requiring antibiotic therapy and possibly admission. The algorithms includes: 1) simple clinical signs with the highest accuracy for identification of serious bacterial infection that should be acceptable to and understandable by the majority of health- care personnel throughout the developing world and should be reproducible within and between observers, 2) key host biomarker POCTs and 3) pulse oximetry. Respiratory rate will also be assessed with a simple automated device (pending availability of an appropriate automated device at the beginning of the study).

The exact final design of the algorithm will be fine-tuned based on further data analyses of the 'Fever Study' and during a study pilot phase. For fever with focal signs (such as skin infections and diarrhea) the algorithm will recommend treatment based on clinical assessment, similar to IMCI. For pneumonia, treatment guidelines will be based on clinical signs (including fast breathing) as well as oximetry and CRP measurement. For unspecific fever, a predictive model including clinical signs and host biomarkers as predictors for serious bacterial infections and for severe disease will be generated based on results from the fever study. Probability of disease will be calculated based on the model and thresholds for admission/antibiotic treatment will be set based on age. All these predictors will be built into an electronic algorithm that will estimate probabilities for each patient to benefit from admission and/or antibiotic treatment.

2.6 Rationale for the clinical study design

This study addresses limitations of currently available clinical management algorithm for children presenting with fever in resource-poor settings. The clinical algorithm to be studied has been carefully crafted based on current evidence of fever management in children less than five years of age. It is based on widely used clinical management tools such as the WHO IMCI guidelines as well as paediatric triage tools. CRP, PCT, oximetry and hemoglobin measurements are used routinely in the management of children with fever in the developed world. However, they have not been integrated into management tools for resource-poor settings. Overall, we expect no adverse events from the use of this new fever management tool which has the potential to be of immense benefit. However, it is essential to establish the safety of this revised tool in a controlled fashion before introducing it into routine practice. The choice to include a control arm (in this instance usual procedures of the health facility based on IMCI) can be questioned, especially so when the quality of care is known to be below standard. However, the control group is critical in our study to ensure safety of the new algorithm. Without control arm, we may miss a deleterious effect of the new algorithm on health outcomes. It is also important to document the impact on the number of antimicrobials prescribed: reducing unnecessary medicine intake is indeed an essential aspect of safety.

3 Clinical Study Objectives and Hypotheses

3.1 Objectives

3.1.1 General Objective

The goal of this project is to improve the health outcome of children with acute fever through rapid and accurate identification of those children at increased risk of life-threatening infections and those children that would most likely benefit from antibiotics, through the use of point-of-care technologies appropriate for implementation at primary care level. Through this approach we also aim at a more rational use of antimicrobials, and therefore mitigation of the development of antimicrobial resistance and drug side effects, as well as reduction in of misallocation of scarce health resources that results from inappropriate antibiotic use, inappropriate referral to higher level of care, and unnecessary inpatient treatment.

3.1.2 Specific Objectives

3.1.2.1 Primary Objective

To compare the clinical outcome of febrile children 2-59 months of age managed using e-POCT (intervention arm), ALMANACH (reference control arm) and routine practice (routine control arm).

3.1.2.2 Secondary Objectives

To compare the rational use of antimicrobials in treating febrile children using e-POCT, ALMANACH and routine care.

To compare the performance of e-POCT, ALMANACH and routine care in identifying children at risk for life-threatening infection among febrile children.

To assess the diagnostic performance of new generation host-biomarkers in identifying children at risk for life-threatening infection and for clinical failure among febrile children (e-POCT and ALMANACH arms).

To measure the proportion of febrile children with hypoxemia, stratified by diagnostic classification (e-POCT arm).

To assess the diagnostic performance of new generation host biomarkers in identifying children in need for antibiotic treatment among febrile children (e-POCT and ALMANACH arms).

3.2 Hypotheses

The hypotheses of this study are as listed.

- Clinical outcome of febrile children aged 2-59 months managed using e-POCT is not inferior to ALMANACH (and routine practice)
- The rational use of antimicrobials for treating febrile children using e-POCT is greater compared to ALMANACH (and routine practice)
- The performance of e-POCT in identifying children at risk for life-threatening infection among febrile children is greater when compared to ALMANACH (and routine practice)
- A combination of new generation host biomarkers will identify additional cases of children at risk for life-threatening infection among febrile children (e-POCT arm) when compared to CRP and PCT alone.
- A combination of new generation host biomarkers will identifying additional cases of children at risk for clinical failure among febrile children (e-POCT arm) when compared to CRP and PCT alone.
- The use of e-POCT will increase the proportion of febrile children identified as having a life-threatening infection when compared to ALMANACH and routine care.

4 STUDY DESIGN

4.1 Primary and secondary outcome measures

The **primary outcome measure** is the proportion of clinical failure by day 7 compared among the 3 study arms.

The **secondary outcome measures** are as follows:

- proportions of secondary hospitalization and death by day 30 compared among the 3 study arms.
- proportions of children prescribed an antibiotic and/or antimalarial treatment at day 0 and by day 7 compared among the 3 study arms.
- proportions of children with hypoxemia, stratified by diagnostic classification (e-POCT arm)

- performance of combinations of host biomarkers in identifying children at risk for life-threatening infections and for clinical failure among children presenting with fever (e-POCT and ALMANACH arms).
- proportion of primarily admitted children compared among the 3 study arms.

Exploratory outcome measure:

- Performance of combinations of host biomarkers in identifying children in need for antibiotic treatment, by type of infection (e-POCT and ALMANACH arms).
- Accuracy of a new automated respiratory rate device in measuring respiratory rate in children with fever compared to gold standard (manual count).

4.2 Design description

This is a randomized controlled non-inferiority trial investigating a new electronic point-of-care tool (e-POCT) for the management of acute febrile episodes among children 2-59 months of age. Patients will be recruited from the outpatient department of municipal/district government health facilities (health centers, dispensaries, and hospitals)in Dar es Salaam, Tanzania. The study sites will be chosen in collaboration with the CMOH of Dar es Salaam. The city centre of Dar es Salaam has been chosen because of its relative low endemicity for malaria allowing better investigation of febrile episodes caused by other diseases than malaria. The study will include children ≥2 months and <60 months of age who present with acute fever for less than 7 days. Study participants will be randomly assigned to be managed by e-POCT (Arm 1, intervention) or by ALMANACH (Arm 2, reference control). In parallel, patients attending the OPD of another municipal or district hospital will be included in the routine care control arm (Arm 3):

1500 patients will be included in each of the e-POCT (intervention) and ALMANACH (reference control) arms and 500 in the routine care control arm (see Section 8.2 for details of sample size calculation). All consecutive patients meeting the inclusion criteria will be included in arms 1 and 2 (total expected number per day per arm = 9). The first 3 consecutive patients (a third of the number included daily in arms 1 and 2) meeting the inclusion criteria will be included in the routine control arm 3 (for details on randomization refer to section 4.4). All children (enrolment arms 1-3) will be evaluated at the time of enrolment (day 0) and will have follow-up evaluations on day 3 and 7 (and 14 if not cured at day 7), or at any time in between in case of clinical deterioration according to caretakers evaluation. All patients will also be followed-up on day 30 via a phone interview to ask for secondary hospitalization or death. We expect to enroll patients for a period of 16 months starting September 2014.

Patients from arms 1 and 2 (e-POCT and ALMANACH) presenting with unspecific fever (expected number of patients: 1200) will also be included into a sub-cohort study that aims at investigating new host biomarkers to predict bacterial disease as described in Section 6.9.

Schematic of study design

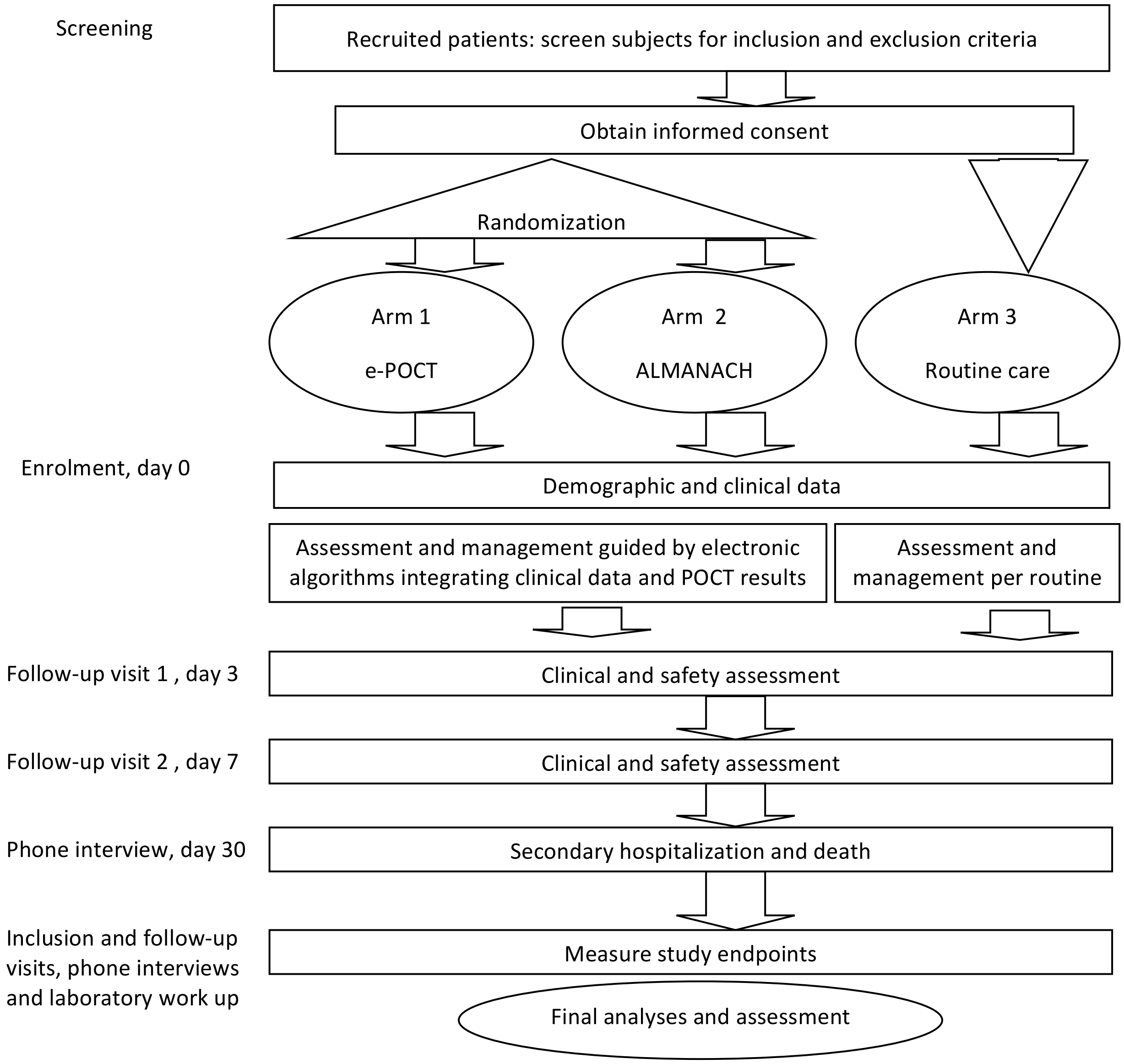

Activities	***Time Period***
Preparation of the study	*6 months (starting March 2014)*
Enrolment	*16 months*
Interim statistical analysis	*After 100 and 500 patients included in each of arms 1 and 2*
End of follow-up	*One month after end of enrolment*
Final statistical analysis	*Three months after end of enrolment*
Final study report	*Six months after end of enrolment*

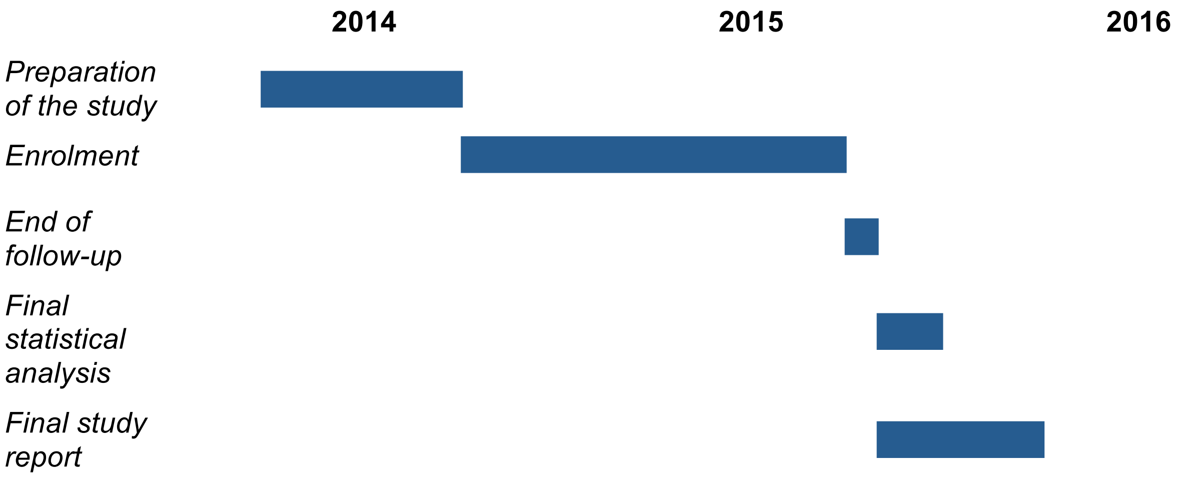

4.3 Intervention

In the intervention arm, patients will be managed by a study clinician who will use the new e-POCT tool. Compliance with the tool will be monitored through conditional electronic algorithms. This tool consists in an electronic algorithm that combines a few key clinical elements (demographics, symptoms and signs) with the result of a series of point-of-care test results to provide one or more diagnostic classification(s) with its(their) probability, recommended treatments, and advice on admission versus discharge home. The algorithm will be built into an android tablet using Open Data Kit collect and OpenMRS (as for ALMANACH) that will serve as an interactive triage tool. More specifically, the android application will be programmed to calculate the probability of serious bacterial infections according to the entered data. If this probability is higher than a pre-defined threshold (high), the diagnosis will be considered as certain and the clinician will be advised to prescribe the appropriate antibiotic and/or to admit the child. If the probability if lower than another pre-defined threshold (low), the diagnosis will be considered as excluded and antibiotics should be withheld and/or the child will be managed as outpatient. If the probability falls in between these two thresholds (high and low) the diagnosis will be considered uncertain, and further testing will be proposed to the clinician. If an antimicrobial or other medication (such as bronchodilators for reactive airway disease) is recommended, the algorithm will specify the medication name along with weight-based dosing recommendations. The oxygen saturation measured by oximeter will be automatically transferred to the tablet to guarantee adherence, avoid transcription errors and gain time (Figure 11).

All study personnel will receive detailed training on the correct use of e-POCT before the start of the study. The algorithm will be tested in a pilot phase before starting the study to fine-tune the content.

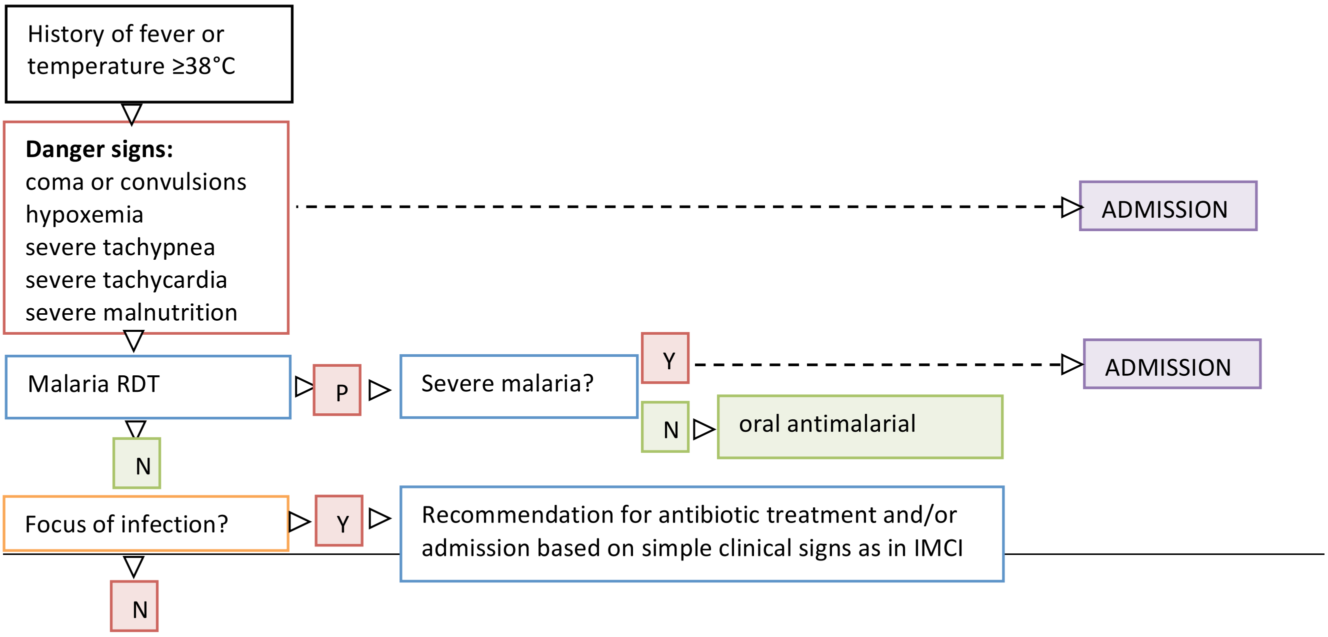

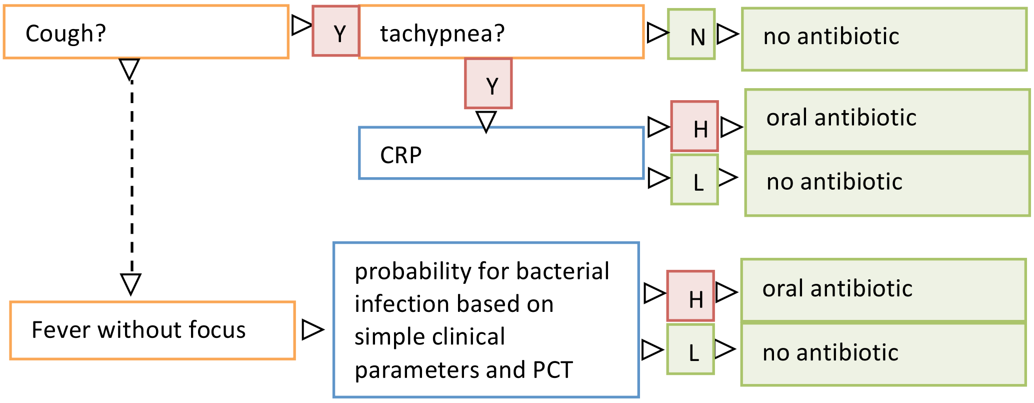

Figure 10: Algorithm based on a few symptoms and signs, POCTs and oximetry that will be used in the e-POCT arm. Y=yes, N=no, H=high, L=low; Branches of the algorithms are not mutually exclusive.

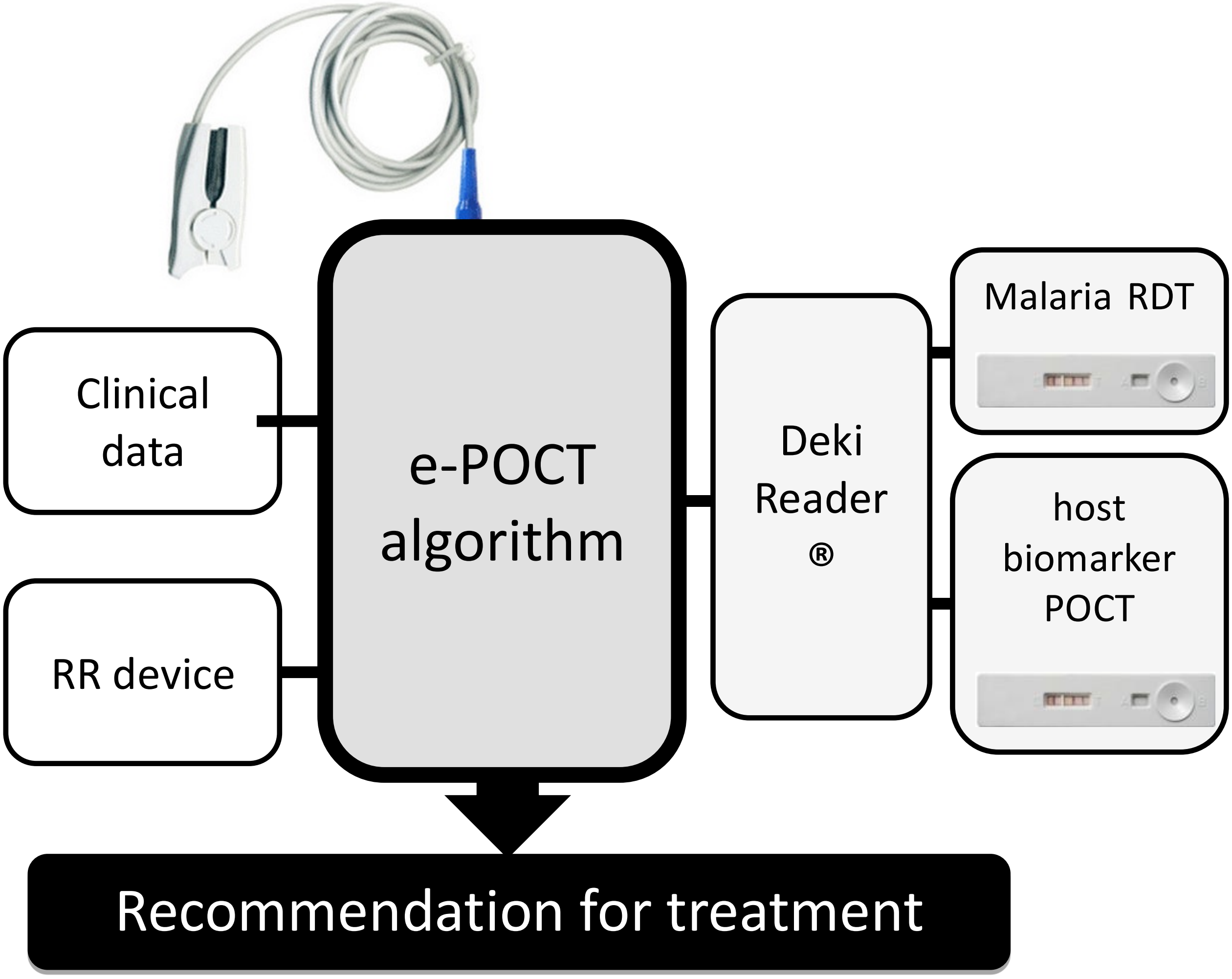

Figure 11: schematic representation of the e-POCT tool

4.4 Measure to minimize bias

Randomization between e-POCT (arm 1) and ALMANACH (arm 2) will be performed at patient level rather than at provider level. This will allow reducing the risk of bias due to a different capacity

between study clinicians in detecting the key clinical signs. Blocks of 14 days will be used to minimize bias introduced by contamination due to switching too frequently from one management tool to another. An interval of 14 days was chosen in order to limit variation of illnesses by seasonality. The risk of contamination between the ALMANACH and e-POCT arms is also limited by the fact that study clinicians will be trained to strictly follow the electronic clinical algorithms and compliance will be monitored by the electronic algorithms themselves. Study clinicians will be explained that the new tools (host biomarkers) available in the e-POCT arm have not been yet evaluated for the outpatient management of febrile children and are thus not recommended for that purpose in Tanzania. Therefore, to be able to assess the performance of these new tools (the objective of the present study), they should only be used in the e-POCT (intervention) and not in the ALMANACH (control arm).

To avoid bias in clinical outcome that could be induced through the ancillary study on performance of biomarkers to identify children with bacterial infections (secondary outcome), the sub-cohort will include all children with unspecific fever from both the intervention (e-POCT) and control (ALMANACH arms).

Patients in the routine care group (arm 3) will be enrolled in a separate outpatient department that is comparable in terms of deserved population and disease epidemiology. To involve a separate outpatient department is necessary to avoid inclusion bias and contamination in the management of patients between the intervention and the routine control group.

5 STUDY POPULATION

5.1 Study site and source population

Patient recruitment will take place in the OPD of the included health facilities in Dar Es Salaam. Dar Es Salaam is the commercial capital of Tanzania. It has relatively low endemicity for malaria: Parasite rates in the community are around 1-4% and only 6-12% of febrile patients are parasitemic[31]. Most patients seeking care at the study health facilities originate from the neighbourhood. Around

Around 5,000-10,000 children between 2 months and 5 years of age seek care at the OPD of these health facilities. Clinicians are instructed to use the national “Standard Treatment Guidelines (STG)” for management of febrile illness in children that are based on WHO IMCI. Patients requiring higher level of care such as parenteral antibiotics and oxygen therapy are admitted to the paediatric ward at the regional referral hospital in the same district.

5.2 Enrolment

This study will screen for enrolment among patients in the OPD of the included health facilities. All consecutive patients presenting to the OPD will be considered for enrolment in the study. Study participants will be identified at the triage areas of the OPD where axillary and tympanic temperatures will be measured. Eligible children will be offered enrolment in the study.

5.3 Inclusion criteria

All of the following inclusion criteria have to be met in order for a patient to be included in the study:

- Age ≥2 months and <60 months of age
- Written informed consent from the child’s parent or caregiver
- Axillary temperature ≥37.5°C and/or tympanic temperature ≥38.0°C
- History of fever for ≤7 days
- First consultation for the current illness
- Live in the catchment area of the health facility

5.4 Exclusion criteria

Participation exclusion criteria:

- Age 60 months or greater
- Age less than 2 months
- Weight less than 2.5kg
- Chief health problem is an injury, trauma or acute poisoning

5.5 Definition of clinical failure

Clinical failure is defined as a patients presenting with:

At any time between initial assessment and day 7:

Danger signs:

- Coma
- More than 2 convulsions within 24hr
- Inability to drink or breastfeed
- Hypoxemia
- Severe tachypnea
- Severe tachycardia

At day 3:

- History of cough and tachypnea
- History of cough and lower chest indrawing
- Significant dehydration

At day 7:

- Fever or temperature ≥38°C
- History of cough and tachypnea
- History of cough and lower chest indrawing
- Diarrhoea (≥ 3 unformed stools/day)
- Significant dehydration
- Serious skin infection
- A new significant symptom or sign related to the acute episode but not present at day 0.

The rate of clinical failure by day 7 will be calculated by censoring a child as failure if: i) he/she presents between day 1 and day 7 a danger signs that was not present at inclusion; ii) he/she meets between day 3 and day 6 the failure criteria of day 3; iii) if he/she meets at day 7 the failure criteria of day 7.

5.6 Criteria for discontinuation of the study

5.6.1 Discontinuation of individual subjects

At any time study participants may withdraw voluntarily from the study, from participating in study procedures, or from receiving any of the study interventions.

Study participant may also be discontinued from study treatment and assessments at any time, at the discretion of the investigator. Specific reasons for discontinuing a subject from the study are:

1. Withdrawal of informed consent.
2. Development of exclusion criteria, or other safety reasons during the study.
3. Incorrect enrolment or randomization of the subject.

If, for any reason, a subject is discontinued from the study before full completion of the study intervention, all planned follow-up visits and final interview on day 30 will attempt to be performed in order to assure full safety evaluation of the intervention. Discontinued subjects will not be replaced.

5.6.2 Discontinuation of the entire study

The principal investigator may terminate the study prematurely, either in its entirety or at a particular site, for reasonable cause provided that written notice is submitted a reasonable time in advance of

the intended termination. No advanced notice is required if the study is stopped due to safety concerns.

Stopping rules:

Interim analyses will be performed after inclusion of 100 and 500 patients in each of the intervention and reference control arms. The absolute difference in the clinical failure rate by day 7 should be no more than 5%, otherwise the study will be discontinued. The stopping rules are based on an alpha of 0.025 for a one-sided confidence interval. The calculations below are based on clinical failure rates of 5% and 10% in the control ALMANACH arm.

number of patients per arms 1 and 2	number of failures expected in ALMANACH arm	number of failures in e-POCT arm if 5% worse than ALMANACH (acceptable)	upper CI bound for 5% more failures: STOP if more than this number
ALMANACH failure rate = 5%, e-POCT not worse than 10%			
100	5	10	40
500	25	50	81
ALMANACH failure rate= 10%, e-POCT not worse than 15%			
100	10	15	52
500	50	75	114

5.7 Participants in sub-cohort

All enrolled children from Arms 1 and 2 (e-POCT and ALMANACH) who present with unspecific fever will be offered participation in a prospective sub-cohort study. These children will be managed according to the algorithm used in the corresponding arm. Blood will be drawn per guidelines below and blood culture will be performed to be able to introduce or adapt the antibiotic prescription if necessary. At the follow-up visit at day 7, blood maybe drawn by fingerprick. Viral and bacterial serologies and PCR will be performed on paired samples collected at day 0 and 7 to be able to retrospectively identify the cause of the fever. The results of the latter investigations are not expected to have any impact on the management of the acute episode because most of the children will be cured in between. They are however necessary to be able to retrospectively establish the precise cause of the febrile acute episode.

5.7.1 Cohort study design

Prospective cohort study.

5.7.2 Cohort study population

Study site, study population, inclusion and exclusion criteria are the same as for the general study as described in Sections 5.

6 STUDY PROCEDURES

6.1 Clinical study schedule

All children (enrolment arms 1-3) will be evaluated at the time of enrolment (day 0). In addition, children will have follow-up evaluations on day 3 and 7 (and 14 if not cured at day 7), or at any time in between in case of clinical deterioration according to the caretakers. A phone call will be done at day 30 to all patients to ensure they have not experienced an intercurrent hospitalization and are still alive.

6.2 Screening

Screen for enrolment will take place among patients attending the OPD of the selected health facilities. Study participants will be identified at the triage area of the OPD where axillary and tympanic temperature will be measured. If axillary temperature is ≥37.5°C and/or tympanic

temperature is ≥38°C, the other inclusion criteria will be checked for by a study recruiter. Eligible children will be offered enrolment in the study. The study clinician will then verify that all inclusion and exclusion criteria are met, explain the study purpose, procedures, benefits and risks in detail to the caretaker and answer to all his/her questions related to the study.

6.3 Enrolment

Written informed consent in Swahili will be obtained from the caregiver prior to any study related intervention.

6.4 Inclusion visit

All study participants will undergo clinical and laboratory evaluation at inclusion= day 0 as per section 6.8. Any concomitant medicines or treatments administered during the two weeks prior to enrolment will be recorded.

6.5 Scheduled follow-up visits

All participants will have follow-up visits at day 3 and An additional follow-up visit will be performed on day 14 if patients are not cured (as defined in Section 5.5) on day 7. Patients in all arms will undergo the same follow-up evaluation as per Section 6.8.

6.6 Unscheduled Follow-up Visits

Unscheduled follow-up visits will be performed at self-presentation of the enrolled patient to the health facilities and if clinical or laboratory evaluations are necessary. The same evaluation as for scheduled follow-up visits will be performed.

6.7 Final follow-up Interview

A phone interview will be performed on day 30 to verify final outcome of the patient, to assess for interim hospitalizations and to assure that the patient is still alive.

6.8 Participant Evaluation

6.8.1 Clinical data collection

A list of demographic and clinical data elements to be collected for each study arm is included in Appendix 15.2. For enrolment arm 3 (routine control) clinical data elements will be collected by a trained study health worker sitting next to the clinician and not interfering with the consultation, in order to ensure that data collection has no influence on routine clinical practice.

6.8.2 Clinical evaluation

For arms 1 and 2 (intervention and reference control), clinical evaluation will be performed by study clinicians for all enrolled children at day 0 and follow-up visits. Clinical evaluation for arm 3 (routine control) will be performed by routine clinicians at day 0 and by study clinicians at follow-up visits. Basic anthropometric measurements (weight and mid upper arm circumference - MUAC) as well as pediatric vital signs will be measured in all children enrolled in e-POCT and ALMANACH. If available an automated tool will be used for measurement of pediatric respiratory rate in the e-POCT arm. Clinical evaluation elements for each arm at day 0 and follow-up are included in Appendix 15.2 and 15.5.

6.8.3 Laboratory specimen collection

Type of specimens that will be collected are described below.

Blood

Blood will be drawn to be able to manage the child's acute febrile illness. An additional amount of blood will be drawn for research purposes. In most children blood draw will be performed by a fingerprick for POCTs (clinical management) and novel biomarker measurement (research purposes). The total amount collected by fingerprick is not to exceed 1 ml for patient comfort. In case the fingerprick is not feasible a venous blood draw will be performed.

For children included in the sub-cohort of the e-POCT and ALMANACH arms (ca 1200 children), as well as children with severe illness and with clinical failure, venous blood draw will be performed as described below.

Blood sample collection will be performed according to recommended limits for blood volume per patient weight. A single venous blood draw, required for clinical and research purposes, will not exceed more than 2.4 ml/kg according to KEMRI and WHO guidelines[87], of which 1.4 ml/kg will be used for clinical management and 1 ml/kg for research purposes.

Collected blood volume for clinical and research purposes will be limited to not exceed the lesser of 5ml/kg total blood with a maximum of 50 mL over an 8 week period and collection may not occur more frequently than 2 times per week as per KEMRI and WHO guidelines. A recent WHO summarized safety limits for blood draw in paediatric patients for research purposes [87]. Blood volumes were chosen in accordance with the 2008 KEMRI standards referenced in this publication as this is the only East African standard available. For patient in the sub-cohort with signs of severe anemia, baseline haemoglobin level will be checked using capillary blood sampling. If the baseline hemoglobin level is lower than 7 mg/dl, a single blood draw will be limited to not exceed the lesser of 5ml for a single blood draw and 10mL total blood with a maximum of 50 mL over an 8 week period.

	Weight			
	2.5-5kg	5-10kg	10-15kg	15-20kg
Maximum allowable volume for single blood draw for clinical and research purposes (mL)	6 - 12 ml	12 - 24 ml	24 - 36 ml	36 - 48 ml
Maximum allowable volume for single blood draw for research purposes (mL)	2.5 – 5 ml	5 – 15 ml	15 ml	15 ml
Max allowable blood volume for clinical and research purpose over 8 week period (mL)	12 - 25 ml	25 – 50 ml	50 ml	50 ml

Table 5 Limitations for blood draw volume

Urine

Per international guidelines, urine specimens will be collected in selected children using a specimen collection cup or by straight catheterization[88, 89]. Urine straight catherization has become the standard for diagnosing urinary tract infections in young children worldwide since urine bag culture alone is not sufficiently reliable for diagnosing urinary tract infections a result of the high contamination rate and high incidence of false-positive results. We choose to implement these international best practises in this study as it would be unethical to propose anything below international standards to our study population, especially given that urine catherization is a routinely used, safe, inexpensive (ca 1000TSH/ catheterization), and quick paediatric procedure. The study team (nurses and clinical officers) has been trained in this standard paediatric procedure by paediatricians and paediatric nurses. Catheter size will be chosen per routine standard operational procedures and are summarized in Table 6.

Age	Size (Fr)
<6 months	5-6
<1 year	6
<2 years	6-8
2-5 years	8

Table 6: examples or urinary catheter size by age

Collected urine specimens will be transferred to a specimen container prior to transfer to the laboratory.

Oropharyngeal (OP)/nasopharyngeal (NP) swabs. *Specimens will be collected with a flocked swab.*

Stool. *Specimens will be collected from diapers or in a stool collection hat and placed in a container for transfer to the laboratory.*

Specimens to be collected at inclusion visit (day 0)

Arm 1 (e-POCT):

- Blood (capillary blood for POCTs, except for children included in the sub-cohort in which venous blood will be drawn per above guidelines)
- urine in selected children with unspecific fever (to be able to retrospectively assess urine dipstick results in case of clinical failure)

Arm 2 (ALMANACH):

- blood (capillary blood for RDTs, except for children included in the sub-cohort in which venous blood will be drawn per above guidelines)
- urine (for urine dipstick in selected children of the entire arm and urine culture in selected children of the sub-cohort study)

Arm 3 (routine practice, control): none for study purposes, at the discretion of the routine clinician for clinical management

Children will be excluded from the study if it is not possible to collect blood. They will however be proposed the best possible clinical management and treatment. If no venous blood can be drawn from a child included in the sub-cohort of arms 1and 2, a fingerprick will be performed instead and he/she will remain included in the study and managed according to study procedures for the corresponding arm.The specific investigations required for the sub-cohort (aimed at identifying pathogens causing the fever) will however not be performed.

Specimens to be collected at follow-up visits (day 3, 7 and when necessary 14)

Arm 1 (e-POCT): capillary blood for POCTs (if necessary for clinical management of clinical failure) and for novel host biomarkers (except for patients included in the subcohort where venous draw will be performed to retrospectively establish the cause of the febrile illness).

Arm 2 (ALMANACH): capillary blood for POCTs and novel host biomarkers (except for patients included in the ALMANACH subcohort where venous draw will be performed to retrospectively establish the cause of the febrile illness).

Arm 3 (routine practice, control): none for study purposes, at the discretion of the routine clinician in case of clinical failure

For patients with severe illness and with clinical failure, venous blood will be drawn to be able to investigate the etiological cause of the failure.

6.8.4 Stored aliquots

Quantity permitting, aliquots of collected specimens will be stored for each patient. Written informed consent for storing of specimens and future use for analyses relevant for the management of febrile illnesses without further consent will be obtained from the parent or guardian. Specimens will then be frozen and part of them will be stored at the Ifakara Health Institute for 2 years and part at the Swiss Tropical and Public Health Institute for 15 years, after which all samples will be destroyed. More specifically, specimens will be stored at IHI for 2 years to conduct tests as specified in section 6.9. For tests specified in section 6.9 for which technical capacity is not available in the country at the moment, selected specimens will be sent to collaborating laboratories outside of Tanzania for further testing. For this purpose, specimens will be stored in Switzerland for up to 15 years. For any further

testing that is beyond the specified scope of this protocol, ethical clearance will be sought from IHI and NIMR. If the guardian does not sign the sample donation form, only investigations included in the present protocol will be performed and samples will be destroyed after 2 years.

In summary, for the 1000 children with documented infection included in the e-POCT intervention arm and the 1000 children with documented infection in the ALMANACH control arm, in whom a max of 1 ml of capillary blood will be drawn, about half of the blood (max 0.5 ml) will remain in Tanzania to perform all the necessary rapid diagnostic tests and the remaining blood will be sent to Switzerland for further analyses (see below). For the children with unspecific fever included in the sub-cohort of the e-POCT and ALMANACH arms, in whom venous blood will be drawn, the amount necessary for research purposes (e.g. 2.5 - 5 ml of blood in a child weighting 2.5 - 5 kg) will be sent to Switzerland. The remaining blood (e.g. 3.5 - 7 ml of blood in a child weighting 2.5 - 5 kg) will stay in Tanzania (see first and second rows of the Table 5 in section 6.8.3).

6.8.5 Laboratory processing

All laboratory tests will be overseen by IHI and Swiss TPH staff. Prior to implementation of the study, IHI and Swiss TPH scientists will establish standardized operating procedures for processing specimens from each of the planned specimen sources. Generally, sample handling, testing and storage will be done in Tanzania through the Ifakara Health Institute laboratory. However, for tests that require equipment or technical expertise not available in Tanzania at the time of the study or after completion of enrollment for tests using stored specimens, part of the specimens will be transferred to the Swiss Tropical and Public Health Institute to perform tests in Switzerland, and will be sent from Switzerland to other collaborating laboratories outside of Tanzania (e.g. Sandra Rotman Centre for Global Health in Canada for the host biomarkers) for further testing. However for capacity building the team is working with local Co-PI who is involved in every part of the study and it is expected that in future through knowledge sharing and training such tests might be available in Tanzania.

6.9 Laboratory tests

6.9.1 Rapid diagnostic tests

Pathogen (malaria and HIV in arm 1 and 2, typhoid in arm 2 and leucocytes/nitrites on urine in arm 2) and host biomarker (CRP, PCT and hemoglobin in arm 1) RDTs will be performed by trained study personnel on site.

6.9.2 Blood culture, serologies, molecular testing

To be able to identify the precise cause of the febrile episode as a reference standard for calculating test performance of emerging host biomakers for infection/inflammation, blood cultures will be performed according to standard procedures using the automated BACTEC system at the nearest laboratory where expertise for this procedure is available. Bacterial identification and antibiotic susceptibility will be performed when the culture is positive per CLSI/EUCAST guidelines. Further serological (such as EBV or CMV serologies) and molecular testing (such as *Rickettsia* PCR) aimed at establishing the cause of the fever episode may be performed if no cause of infection could be established by blood culture alone.

6.9.3 Emerging host biomarkers

Validation testing of the emerging host biomarkers, including inflammation, endothelial biomarkers, will be performed pending availability. Results of emerging tests will not be given to participants and will not be used clinically for fever management as they are not yet validated for clinical management.

6.9.4 Other tests

6.9.4.1 Other bacterial cultures

Routine cultures on specimens from skin and soft tissue infections may be performed to allow appropriate clinical management and to determine the epidemiology of bacterial drug resistance in the Tanzanian community. Bacterial surveillance cultures on NP and skin swabs as well as stool specimens may be performed to provide epidemiological data specific to Tanzania.

6.9.4.2 HIV testing

HIV voluntary counselling and testing will be offered to all children fulfilling testing criteria per Tanzanian IMCI guidelines, as well as to all children with unknown HIV status. Testing will be done according to age-based national guidelines. For infants older than 18 months, HIV rapid test will be performed. For infants less than 18 months with HIV exposure (maternal HIV status positive or rapid test positive) DNA PCR by dried blood spot testing will also be performed as per Tanzanian national guidelines. In case of reactive tests and indeterminate results, additional testing will be performed according to current national guidelines. All children with newly diagnosed HIV infection will be referred for HIV care services per national guidelines. If an infant tests positive for HIV and maternal status is unknown, the infant's mother will be offered HIV testing and referred to HIV care and treatment if positive.

For study purposes, unlinked anonymous testing for HIV will be performed on children not tested during study visits, after the end of the study, once the database and the samples will have been fully anonymized.

6.9.4.3 Chest ultrasound

Children with clinical pneumonia in arms 1 and 2 (e-POCT and ALMANACH) will undergo chest ultrasound to assess the presence of ultrasound findings compatible with pneumonia.This will allow the evaluation the potential of host biomarkers to predict documented pneumonia. Ultrasound rather than chest X-ray was chosen to avoid radiation exposure of the child [90].

6.10 Clinical care

For arms 1 and 2 (e-POCT and ALMANACH) treatment guidance will be provided based on pre-defined algorithms that use both clinical data and POCTs results. Diagnostic test results will be available to the primary treating clinician.

The electronic algorithm will integrate the clinical data and the results of the POCTs to provide guidance to the clinician for the decision to:

1. give antibiotic treatment
2. give antimalarial treatment
3. admit to the hospital

Ultimately, treatment decisions will remain the responsibility of the primary treating physician. Certain emergency treatments such as IV antimicrobials, IV hydration, and oxygen therapy will be given as required by the study clinician in the outpatient setting in order to bridge the time to admission to the hospital ward. Once admitted, all patients will be managed by hospital personnel per routine guidelines.

In arm 3 (routine care), clinical care will be left at the discretion of the clinician in charge. The study team will ensure that usual diagnostic tests, such as malaria RDT, and first-line medicines for febrile children are made available also for the control health facilities.

7 ASSESSMENT OF SAFETY

The safety of the new e-POCT tool will be assessed by comparing the clinical outcome by day 7 between children managed with e-POCT and with ALMANACH. The clinical outcome with e-POCT should be at least as good as with ALMANACH (non-inferiority trial). A comparison will also be made between the clinical outcome of children managed with e-POCT and with routine care, to ensure that the new tool is not provoking any harm compared to the present practice in Tanzania. Based on the experience with IMCI and with ALMANACH (an updated version of IMCI) in previous studies, we expect however that the clinical outcome of children will be better in the e-POCT and ALMANACH arms compared to the routine care arm. The whole study will be overseen by the DSMB board who will ensure, in consultation with the principal investigators, that all predefined safety criteria are met (see Section 11.1).

8 STATISTICS

8.1 Definition of outcome measures

The **primary outcome measure** is the difference between proportions of clinical failure (as defined in section 5.5) by day 7 compared among the 3 study arms and the s**econdary outcome measures** are the proportions of death and of secondary hospitalization by day 30, of appropriate antibiotic and antimalarial prescriptions by day 7, and of primary hospitalization, compared among the 3 study arms.

8.2 Justification of number of study subjects

Sampling

This study will enrol all consecutive eligible and consenting patients in the outpatient clinics of the included health facilities during the study period.

Sample Size Determination

The sample size calculation is based on non-inferiority. We wish to precisely estimate the difference in proportions with clinical failure in the e-POCT and ALMANACH groups in order to be able to conclude non-inferiority in the e-POCT arm. The confidence interval for the difference in proportions should not be wider than +/-3%. We assume that the proportion of clinical failure by day 7 is 10% is both groups.

The sample size has been calculated using the following equation for non-inferiority:

$$\varepsilon = p_1 - p_2$$

$$H_0: \varepsilon \leq \delta \ \ vs \ \ H_1: \varepsilon > \delta$$

$$k = n_1/n_2$$

$$n_2 = \frac{\left(z_\alpha + z_\beta\right)^2}{(\delta - \varepsilon)^2}\Big(\big(p_1(1-p_1)\big)/\big(k + p_2(1-p_2)\big)\Big)$$

where p_1= proportion of clinical failure by day 7 in control arm, p_2= proportion of clinical failure by day 7 in intervention arm, α=level of significance, β=1-power, δ=the acceptable difference in proportion of clinical failure between the 2 arms, n_1=number of patients in the control arm and n_2=number of patients in the intervention arm.

The following assumptions were made for sample size calculation:

p_1 ALMANACH	10%
p_2 e-POCT	10%
α	0.05
β	0.2
δ	0.03
n_1:n_2	1:1

The proportion of clinical failure by day 7 in control arm (p_1) is based on the results of the previous study where children were managed with ALMANACH, taking the sub-group of children having the same inclusion criteria as for the present study.

The clinical outcome by day 7 is expected to be better in the e-POCT arm compared to routine care and therefore a very small sample size is needed to be able to show that e-POCT is not inferior to routine care. The number of patients needed in the routine care arm has thus been calculated to ensure that the distribution of main complaints will not be significantly different between the 2 arms (assumptions: p_1= p_2=15%, α=0.05, β=0.2, δ=0.05, n_1:n_2=1:3).

	Sample size per arm	Sample size taking into account exclusions and drop-outs
e-POCT (=Arm 1)	1237	1500
ALMANACH (=Arm 2)	1237	1500
Routine care (=Arm 3)	420	500
**TOTAL number of patients included in the 3 arms**	**2894**	**3500**

8.3 Statistical analysis

Analyses will be conducted using commonly available software packages, such as SAS, STATA, R or SPSS. The main analyses will be as follows:

- Difference between the proportions of children with clinical failure by day 7 in the 3 study arms.
- Difference between the proportions of children with secondary hospitalizations and who died by day 30 in the 3 study arms.
- Risk ratio of the proportions of children prescribed antibiotics and antimalarials at day 0 and by day 7 in the 3 study arms.
- Diagnostic performance of 1) individual host biomarker(s) based on crude positive and negative likelihood ratios, and on the area under the curve (AUC) of receiver operator characteristic (ROC) curves, and 2) combined biomarkers based on sensitivity and specificity generated by classification and regression trees (CART), to predict presence and development of a life-threatening disease, clinical failure, or an infection requiring antibiotic treatment.
- Risk ratio of the proportions of children primarily admitted in the 3 study arms.

An intention to treat (ITT) approach will followed, i.e. statistical analysis of safety will be based on data from all patients who were randomized and from whom meaningful data were collected.

8.4 Data management

The investigator will prepare and maintain adequate and accurate source documents designed to record all observations and other pertinent data for each subject enrolled into the study. Study personnel will enter data related to the subject's visit into the protocol-specific electronic Case Report Form (eCRF). Subjects will not be identified by name in the study database but will be identified by a unique identifier. Data will be entered directly into an Open Data Kit (ODK) in a android tablet. The tablet has real-time error, range and consistency checks and date are transferred to the central database at regular intervals. Standard operational procedures for data quality and control will be developed and implemented. If a correction is required for an eCRF, the time and date stamps will track the person entering or updating eCRF data and creates an electronic audit trail.

To protect patient confidentiality, each patient in the study will be assigned a unique identifier. This unique identifier will be used for specimens and data collected in this study so that the data can be

linked. All personnel who have access to data forms will be asked to sign confidentiality agreements. Identifying information will not be entered in the electronic dataset. No patient names will be entered into analysis databases. All data collection instruments will be stored in closed, locked file cabinets with restricted access at the IHI/ Swiss TPH offices in Dar Es Salaam. The electronic database is safeguarded against unauthorized access by established security procedures; appropriate backup copies of the database and related software files will be maintained. Databases are backed up by the database administrator in conjunction with any updates or changes to the database. At critical junctures of the protocol (e.g., production of interim reports and final reports), data for analysis is locked and cleaned per established procedures.

9 DUTIES OF THE INVESTIGATOR

9.1 Investigator's confirmation

This study will be conducted in compliance with the protocol and the International Conference on Harmonisation Good Clinical Practice E6 (ICH-GCP).

All protocol modifications must be documented in writing. A protocol amendment can be initiated by any of the investigators. The Investigator will provide the reasons for the proposed amendment and will discuss with the Principal Investigator. Any protocol amendment must be approved and signed by the Principal Investigator and must be submitted to the Tanzanian Independent Ethics Committee (IEC) for information and approval, in accordance with local requirements, and to regulatory agencies if required.

9.2 Damage coverage

In the event of a side effect or injury to the participant, the principal investigator and institutions ensure that appropriate medical care, as determined by the Investigator or his/her designee, will be provided. All subjects of the study will have insurance coverage for research-related injuries and/or medical problems determined to result from receiving the intervention. Necessary treatment and proper follow-up care will be made available to the participant free of charge. Compensation will be guided by the respective insurance policies that adhere to the Tanzanian national guidelines.

10 ETHICAL CONSIDERATIONS

The principal investigators will ensure that this study is conducted in full compliance with the principles of the "Declaration of Helsinki" (as amended in Tokyo, Venice, Hong Kong, and South Africa), and with the laws and regulations of Tanzania. Human dignity will be preserved by adherence to the following ethical principles:

- Non-instrumentalisation: participating researchers guarantee to never use individuals simply as a means but always as an end of their own;
- Privacy: the ethical principle of not invading a person's right to privacy will be ensured;
- Non-discrimination: patients involved in the study will receive equal treatment, unless there are medical reasons that justify difference in treatment or distribution of health care resources;
- Informed consent: all patients involved in the study will express their consent after being adequately informed;
- The Precautionary principle: researchers will respect their moral duty of continuous risk assessment;
- All human samples will be coded in the enrolling centre and remain anonymous for the rest of the researchers involved in the project. In the same manner, all data obtained using these samples will follow the Directive 95/46/EC of the European Parliament and of the Council of

24 October 1995 on the protection of individuals with regard to the processing of personal data and on the free movement of such data.

10.1 Independent Ethics Committee (IEC)

The protocol of this study will be submitted for approval to the local ethical boards, i.e. institutional review board (IRB) of IHI and national Ethical Boards, i.e. national institute for medical research (NIMR), in Tanzania as well as to the Swiss Ethical board of Basel, EKBB. The principal investigators will submit the protocol with all necessary documents to these boards.

10.2 Evaluation of the risk-benefit ratio

The project has been already discussed in depth with the different partners, including the ethical considerations. When a project is aiming at improving adherence to universally accepted recommendations such as IMCI guidelines, there is no definite harm to be expected. On the reverse, there is a hope that patients, and especially children will benefit from the intervention planned. All procedures employed in the algorithm are part of routine paediatric clinical practice. The usual criticism of electronic-assisted diagnosis and treatment is that clinicians may feel less concerned and may not investigate the peculiarities of the patients they have to care. However, we have seen during our previous project that the level of care is in general quite poor and it is unlikely that such a tool could lead to worse care. What has been observed in the past with such tools is that the clinicians tend to acquire an automatism that is beneficial for the patient, as long as the automatism complies with updated guidelines, which will be the case in this study. These tools are also very useful as a teaching and training material. The choice to include a control arm (in this instance usual procedures of the health facility) could be questioned, especially so when the quality of care is known to be below standard. However, the control group is critical in our study to ensure safety of the new clinical algorithm. Without control arm, we may miss a deleterious effect of the new algorithm on health outcomes. It is also important to document the impact on the number of antimicrobials prescribed: reducing unnecessary medicine intake is indeed an essential aspect of safety.

10.3 Subject information and consent

Children eligible for this study will all be less than 5 years old, which is less than the age of consent or assent in Tanzania. Written informed consent will be obtained from the caregiver of eligible children prior to the collection of demographic data/contact information, initiation of screening and diagnostic testing, or collection of specimens for storage for future use. The caregiver will be given the opportunity to ask questions. If there is any part of the consent form not understood, the interviewer will attempt to provide additional explanation and clarification and will review the consent form up to a maximum of 3 times; if the caregiver repeatedly does not answer clarifying questions correctly, then the interviewer may conclude that the person does not understand or is not capable of providing consent. The caregiver will be given a study information sheet that summarizes the study and has study-related contact information. They will also be offered a blank copy of the consent form to keep for their records. The signed consent form will be retained and stored securely separate from the data collection forms. Consent forms and the study information sheet will be translated into Kiswahili by a certified translator or using the translation/back translation method. The translated forms will be assessed by a Tanzanian member of the IHI/ Swiss TPH staff or Ministry of Health who is fluent in Kiswahili and English to ensure their readability.

10.4 Subject Confidentiality

All records identifying the subjects will be kept confidential and, to the extent allowed by the applicable laws and/or regulations, will not be made publically available. To minimize the risk, all laboratory specimens, evaluation forms, reports, and other records will be identified by coded number only. All computer entry and networking programs will be performed encrypted and password-protected.

10.5 Subjects requiring particular protection

Pregnant women, human fetuses, or neonates: Pregnant women, human fetuses and neonates will be excluded from this study because this study will only include children older than two months and younger than 5 years of age.

Prisoners: Persons incarcerated at the time of study enrolment will not be enrolled in this study because this study enrolls only young children and because enrolment sites do not include prisons or prison health care facilities. However, children who may reside at a prison because their mother is incarcerated may be included in the study.

Children: Persons aged 2-59 months will be included in this study because this study aims to determine the optimal approach to managing fever in this age group.

10.6 Compensation for inconvenience

The participants will be compensated adequately to cover the travel costs to and from the health facility and the time spent to participate in this study.

11 Quality control and quality assurance

Clinical data will be entered directly into an open data kit (ODK) in an android tablet. The tablet has real-time error, range and consistency checks and data are transferred to the central database at regular interval. POCTs results will be directly entered into the tablet. All clinical information will be entered in a central data base that will include a patient ID and sample identification code. This central data base will be exchanged between IHI and Swiss TPH investigators. The results of the sample analysis will be added in the central data base using the patient ID and sample identification code for reconciliation. In order to avoid entry errors, regular check will be performed by the Principal Investigator. Data back-up will be performed on a regular base. We confirm that our study will be conducted according to the study protocol, good epidemiological practice and the applicable regulatory requirements. Any protocol amendments would be submitted for review to the ethics committees. Any unforeseen event posing a risk to the participants would be communicated to the ethical committees. The investigators have sufficient time to properly conduct and complete the study within the agreed study period and have available an adequate number of qualified staff and adequate facilities for the foreseen duration of the study to conduct the study properly and safely. Informed consent will be obtained from each participant and documented, the version approved by the ethics committees will be adhered to. The investigators will ensure the accuracy and completeness of the data reported in the study and will timely submit the required progress and final reports. Compliance to all processes will be regularly supervised by a senior scientist from the Swiss TPH.

11.1 Data and safety monitoring board

A formal Data and Safety Monitoring Board (DSMB) will be established to oversee the study that will include at least one local paediatrician. The DSMB will have a charter and formal stopping rules for the study. The DSMB will evaluate the data at the interim analysis and provide recommendations to the principal investigator with regard to continuation of the study.

12 Capacity buiding at the Ifakara Health Institute and the Swiss TPH

A Tanzanian recently graduated Medical Doctor has been employed by IHI to design, conduct and supervise the present study in collaboration with a Swiss Medical Doctor (paediatrician and infectious diseases fellow) who has several years of clinical and research experience in paediatrics and infectious diseases, as well as a Master in Public Health. They will mutually benefit from their clinical and research experience and increase their knowledge in terms of running a clinical study and

interpreting and disseminating the results. If the Tanzanian Medical Doctor proves to be competent during the first phases of the study, fulfils all University requirements and is motivated to do so, he will be offered to participate in a post-graduate master or PhD program. If the Swiss Medical Doctor proves to be competent during the first phases of the study, fulfils all University requirements and is motivated to do so, she will be offered to participate in a PhD program.

13 DISSEMINATION OF RESULTS AND PUBLICATION POLICY

13.1 Dissemination to scientific community

At the end of the study, findings will be summarized in chart profiles and discussed first with the clinicians involved in data collection. Interpretation of results will be put into perspective after discussions with a qualitative assessment from direct users and with the health authorities. The latter will be involved in the dissemination of results in the offices of the Tanzanian National Malaria Control Programme, the WHO country office and Ministry of Health. An international meeting involving clinical researchers, health systems researchers, paediatricians, e-heath technologists, POCT manufacturers, WHO experts, and members of UNICEF and PATH, will then be organized in Dar es Salaam on the theme of 'e-health and POCT: a change of clinical practice paradigm'. Appropriate technology platforms and usefulness of POCT in clinical practice (and surveillance) will be discussed and agreed upon for further development. In particular, harmonization of open-access platforms for surveillance, as well as clinical management, will be attempted. The relevance of proceeding into larger-scale implementation and evaluation of the tool, or of an optimized tool, in different settings will be discussed and potential interested collaborators in other developing countries (especially Africa) identified. Several scientific papers describing the results of the study will also be published in local and international peer and non-peered reviewed journals.

13.2 Information of community and policy makers

The multiple affiliations of the principal investigator and the long-term partnership built with the Ifakara Health Institute and health authorities (Tanzanian Ministry of Health, Dar es Salaam City Medical Office of Health and Ilala District Medical office) ensures that the innovative tool will then find its way into application in routine practice, if successful. The principal investigator is also part of the Operations Research Group of the iCCM task force, an association of multilateral and bilateral agencies and NGOs (UNICEF, WHO, Save the Children, USAID and MCHIP) working to promote integrated community level management of childhood illness. All operational research findings can thus be disseminated quickly through this important channel. An international agreement on the way forward should help to seek support for further development, evaluation and potential large-scale implementation of this tool from funders such as PATH and the Bill and Melinda Gates Foundation, the latter having already awarded a grant to us for the initial biomarker assessment of the Tanzanian aetiology of fever study. The prospect for applying this triage tool at large scale in developing countries is high since the PI takes part to all discussions on the management of febrile illnesses within WHO that lead to policy changes. If sufficient evidence accumulates to show that our approach is sound, we will certainly be invited by WHO or governments to contribute to discussion and propositions for a policy change. The Maternal and Child Health department of WHO is then expected totake the lead of updating the IMCI algorithm adapt the existing guidelines and training tools (that are already available to countries in electronic version to allow quick update and distance learning) and disseminate them to countries.
In parallel to a policy change, a public-private partnership should be built to ensure that the new biomarkers POCTs are made available at a competitive price for resource-limited countries, similar to what has been done for artemisin-combination therapies or other essential medicines or devices. The Foundation for Innovative New Diagnostics based in Geneva has been an important player to help making diagnostic devices reliable, available and affordable, and we are in permanent contact with them for the present and other projects. Because the tools we are developing have a potential application also in rich countries, manufacturers will be able to sell the POCTs at a differential price to recover investments. Once available on the market, countries could then buy these new POCTs through the new funding mechanisms of the Global Fund to fight AIDS, tuberculosis and malaria.

Indeed, in several countries, the Global fund has already engaged discussions to encourage and contribute to the implementations of the integrated management of febrile illnesses.

14 LITERATURE

1. Liu L, Johnson HL, Cousens S, et al. Global, regional, and national causes of child mortality: an updated systematic analysis for 2010 with time trends since 2000. *Lancet*. 2012;379(9832):2151–61. doi:10.1016/S0140-6736(12)60560-1.

2. National Bureau of Statistics (NBS) [Tanzania] and ORC Macro. *Tanzania Demographic and Health Survey 2010*. Dar es Salaam, Tanzania: NBS and ICF Macro; 2011.

3. D'Acremont V, Kahama-Maro J, Swai N, Mtasiwa D, Genton B, Lengeler C. Reduction of anti-malarial consumption after rapid diagnostic tests implementation in Dar es Salaam: a before-after and cluster randomized controlled study. *Malar J*. 2011;10(1):107. doi:10.1186/1475-2875-10-107.

4. Bryce J, Victora CG, Habicht J-P, Vaughan JP, Black RE. The multi-country evaluation of the integrated management of childhood illness strategy: lessons for the evaluation of public health interventions. *Am J Public Health*. 2004;94(3):406–15. Available at: http://www.pubmedcentral.nih.gov/articlerender.fcgi?artid=1448266&tool=pmcentrez&rendertype=abstract.

5. Tanzania IMCI multi-country evaluation health facility survey study group. The effect of Integrated Management of Childhood Illness on observed quality of care of under-fives in rural Tanzania. *Health Policy Plan*. 2004;19(1):1–10. doi:10.1093/heapol/czh001.

6. Rakha MA, Abdelmoneim A-NM, Farhoud S, et al. Does implementation of the IMCI strategy have an impact on child mortality? A retrospective analysis of routine data from Egypt. *BMJ Open*. 2013;3(1):1–10. doi:10.1136/bmjopen-2012-001852.

7. Victora CG, Huicho L, Amaral JJ, et al. Are health interventions implemented where they are most needed? District uptake of the integrated management of childhood illness strategy in Brazil, Peru and the United Republic of Tanzania. *Bull World Health Organ*. 2006;84(10):792–801. Available at: http://www.pubmedcentral.nih.gov/articlerender.fcgi?artid=2627500&tool=pmcentrez&rendertype=abstract.

8. Beracochea E, Dickson R, Freeman P, Thomason J. Case management quality assessment in rural areas of Papua New Guinea. *Trop Doct*. 1995;25(2):69–74. Available at: http://www.ncbi.nlm.nih.gov/pubmed/7778198. Accessed December 13, 2013.

9. Horwood C, Vermaak K, Rollins N, Haskins L, Nkosi P, Qazi S. An evaluation of the quality of IMCI assessments among IMCI trained health workers in South Africa. *PLoS One*. 2009;4(6):e5937. doi:10.1371/journal.pone.0005937.

10. Tanzanian Ministry of Health and Social Welfare and World Health Organization. Integrated Management of Childhood Illness.Chart Booklet, Dar es Salaam, Tanzania, 22 April 2012.

11. Addo-Yobo E, Anh DD, El-Sayed HF, et al. Outpatient treatment of children with severe pneumonia with oral amoxicillin in four countries: the MASS study. *Trop Med Int Health*. 2011;16(8):995–1006. doi:10.1111/j.1365-3156.2011.02787.x.

12. World Health Organization. *World Malaria Report 2012*.; 2012. Available at: http://www.who.int/malaria/publications/world_malaria_report_2012/report/en/index.html.

13. D'Acremont V, Lengeler C, Genton B. Reduction in the proportion of fevers associated with Plasmodium falciparum parasitaemia in Africa: a systematic review. *Malar J*. 2010;9:240. doi:10.1186/1475-2875-9-240.

14. English M, Reyburn H, Goodman C, Snow RW. Abandoning presumptive antimalarial treatment for febrile children aged less than five years--a case of running before we can walk? *PLoS Med*. 2009;6(1):e1000015. doi:10.1371/journal.pmed.1000015.

15. Rossi IA, D'Acremont V, Prod'Hom G, Genton B. Safety of falciparum malaria diagnostic strategy based on rapid diagnostic tests in returning travellers and migrants: a retrospective study. *Malar J*. 2012;11:377. doi:10.1186/1475-2875-11-377.

16. D'Acremont V, Malila A, Swai N, et al. Withholding antimalarials in febrile children who have a negative result for a rapid diagnostic test. *Clin Infect Dis An Off Publ Infect Dis Soc Am*. 2010;51(5):506–511. doi:10.1086/655688.

17. Senn N, Rarau P, Manong D, et al. Rapid diagnostic test-based management of malaria: an effectiveness study in Papua New Guinean infants with Plasmodium falciparum and Plasmodium vivax malaria. *Clin Infect Dis an Off Publ Infect Dis Soc Am*. 2012;54(5):644–651. doi:10.1093/cid/cir901.

18. D'Acremont V, Lengeler C, Genton B. Reduction in the proportion of fevers associated with Plasmodium falciparum parasitaemia in Africa: a systematic review. *Malar J*. 2010;9:240. doi:10.1186/1475-2875-9-240.

19. D'Acremont V, Lengeler C, Mshinda H, Mtasiwa D, Tanner M, Genton B. Time to move from presumptive malaria treatment to laboratory-confirmed diagnosis and treatment in African children with fever. *PLoS Med*. 2009;6(1):e252. doi:10.1371/journal.pmed.0050252.

20. World Health Organization. *Guidelines for the treatment of malaria. Second edition March 2010*.; 2010. Available at: http://www.who.int/malaria/publications/atoz/9789241547925/en/index.html.

21. Batwala V, Magnussen P, Nuwaha F. Antibiotic use among patients with febrile illness in a low malaria endemicity setting in Uganda. *Malar J*. 2011;10:377. doi:10.1186/1475-2875-10-377.

22. Baiden F, Webster J, Owusu-Agyei S, Chandramohan D. Would rational use of antibiotics be compromised in the era of test-based management of malaria? *Trop Med Int Health*. 2011;16(2):142–4. doi:10.1111/j.1365-3156.2010.02692.x.

23. Okeke IN, Laxminarayan R, Bhutta Z a, et al. Antimicrobial resistance in developing countries. Part I: recent trends and current status. *Lancet Infect Dis*. 2005;5(8):481–93. doi:10.1016/S1473-3099(05)70189-4.

24. Ashley EA, Lubell Y, White NJ, Turner P. Antimicrobial susceptibility of bacterial isolates from community acquired infections in Sub-Saharan Africa and Asian low and middle income countries. *Trop Med Int Heal TM IH*. 2011;16(9):1167–1179. doi:10.1111/j.1365-3156.2011.02822.x.

25. Moyo SJ, Steinbakk M, Aboud S, et al. Penicillin resistance and serotype distribution of Streptococcus pneumoniae in nasopharyngeal carrier children under 5 years of age in Dar es Salaam, Tanzania. *J Med Microbiol*. 2012;61(Pt 7):952–959. doi:10.1099/jmm.0.042598-0.

26. Moyo S, Aboud S, Kasubi M, Maselle SY. Bacteria isolated from bloodstream infections at a tertiary hospital in Dar es Salaam, Tanzania--antimicrobial resistance of isolates. *S Afr Med J*. 2010;100(12):835–838.

27. Temu MM, Kaatano GM, Miyaye ND, et al. Antimicrobial susceptibility of Shigella flexneri and S. dysenteriae isolated from stool specimens of patients with bloody diarrhoea in Mwanza, Tanzania. *Tanzan Health Res Bull*. 2007;9(3):186–189.

28. Blomberg B, Manji KP, Urassa WK, et al. Antimicrobial resistance predicts death in Tanzanian children with bloodstream infections: a prospective cohort study. *BMC Infect Dis*. 2007;7:43. doi:10.1186/1471-2334-7-43.

29. World Health Organization, Communicable Diseases. Interventions and strategies to improve the use of antimicrobials in developing countries : a review. 2001.

30. Okeke IN, Klugman KP, Bhutta ZA, et al. Antimicrobial resistance in developing countries. Part II: strategies for containment. *Lancet Infect Dis*. 2005;5(9):568–580. doi:10.1016/S1473-3099(05)70217-6.

31. D’Acremont V, Kilowoko M, Kyungu E, et al. Beyond malaria: etiologies of fever in outpatient Tanzanian children. *N Engl J Med*. 2014;370(9):809–817. doi:10.1056/NEJMoa1214482.

32. Rambaud-Althaus C, Shao A, Kahama-Maro J, Genton B D V. *Management of Childhood Illnesses: development of a new algorithm (ALMANACH) to promote evidence-based medicine and rational use of drugs.* Abstract book of the 7th ECTMIH, Barcelona 3-6 Oct 2011; 2011.

33. Shao A, Rambaud-Althaus C, Perri S, et al. Safety of a new ALgorithm for the MANAgement of CHildhood illness (ALMANACH) to improve quality of care and rational use of drugs. *Abstr Abstr B 7th ECTMIH, Barcelona 3-6 Oct 2011*.

34. Rambaud-Althaus C, Shao A, Swai N, et al. Smartphones to improve health workers performance and rational use of drugs for the management of childhood illnesses in a low resource setting. *Abstr Abstr B to 61th Annu Meet Am Soc Trop Med Hyg Atlanta, USA, 11-14 Nov 2012*.

35. National Institute of Health and Care Excellence. Feverish illness in children Assessment and initial management in children yonger than 5 years. *NICE Clin Guidel*. 2013;(May).

36. Gorelick MH, Lee C, Cronan K, Kost S, Palmer K. Pediatric emergency assessment tool (PEAT): a risk-adjustment measure for pediatric emergency patients. *Acad Emerg Med*. 2001;8(2):156–62. Available at: http://www.ncbi.nlm.nih.gov/pubmed/11157292. Accessed December 19, 2013.

37. Baumann MR, Strout TD. Evaluation of the Emergency Severity Index (version 3) triage algorithm in pediatric patients. *Acad Emerg Med*. 2005;12(3):219–24. doi:10.1197/j.aem.2004.09.023.

38. Tanabe P, Gimbel R, Yarnold PR, Adams JG. The Emergency Severity Index (version 3) 5-level triage system scores predict ED resource consumption. *J Emerg Nurs*. 2004;30(1):22–9. doi:10.1016/j.jen.2003.11.004.

39. Gouin S, Gravel J, Amre DK, Bergeron S. Evaluation of the Paediatric Canadian Triage and Acuity Scale in a pediatric ED. *Am J Emerg Med*. 2005;23(3):243–7. Available at: http://www.ncbi.nlm.nih.gov/pubmed/15915392. Accessed December 19, 2013.

40. Chamberlain JM, Patel KM, Pollack MM. The Pediatric Risk of Hospital Admission score: a second-generation severity-of-illness score for pediatric emergency patients. *Pediatrics*. 2005;115(2):388–95. doi:10.1542/peds.2004-0586.

41. Neuman MI, Monuteaux MC, Scully KJ, Bachur RG. Prediction of pneumonia in a pediatric emergency department. *Pediatrics*. 2011;128(2):246–53. doi:10.1542/peds.2010-3367.

42. Bleeker SE, Moons KG, Derksen-Lubsen G, Grobbee DE, Moll H a. Predicting serious bacterial infection in young children with fever without apparent source. *Acta Paediatr*. 2001;90(11):1226–32. Available at: http://www.ncbi.nlm.nih.gov/pubmed/11808890.

43. Maguire JL, Kulik DM, Laupacis A, Kuppermann N, Uleryk EM, Parkin PC. Clinical prediction rules for children: a systematic review. *Pediatrics*. 2011;128(3):e666–77. doi:10.1542/peds.2011-0043.

44. Van den Bruel A, Haj-Hassan T, Thompson M, Buntinx F, Mant D. Diagnostic value of clinical features at presentation to identify serious infection in children in developed countries: a systematic review. *Lancet*. 2010;375(9717):834–45. doi:10.1016/S0140-6736(09)62000-6.

45. Craig JC, Williams GJ, Jones M, et al. The accuracy of clinical symptoms and signs for the diagnosis of serious bacterial infection in young febrile children: prospective cohort study of 15 781 febrile illnesses. *BMJ*. 2010;340:c1594. doi:10.1136/bmj.c1594.

46. Nigrovic LE, Kuppermann N, Macias CG, et al. Clinical prediction rule for identifying children with cerebrospinal fluid pleocytosis at very low risk of bacterial meningitis. *JAMA*. 2007;297(1):52–60. doi:10.1001/jama.297.1.52.

47. Oostenbrink R, Moons KG, Donders AR, Grobbee DE, Moll HA. Prediction of bacterial meningitis in children with meningeal signs: reduction of lumbar punctures. *Acta Paediatr*. 2001;90(6):611–7. Available at: http://www.ncbi.nlm.nih.gov/pubmed/11440091. Accessed December 18, 2013.

48. The Young Infants Clinical Study Group. Clinical signs that predict severe illness in children under age 2 months: a multicentre study. *Lancet*. 2008;371(9607):135–42. doi:10.1016/S0140-6736(08)60106-3.

49. Oostenbrink R, Thompson M, Steyerberg EW. Barriers to translating diagnostic research in febrile children to clinical practice: a systematic review. *Arch Dis Child*. 2012;97(7):667–72. doi:10.1136/archdischild-2011-300667.

50. Thompson M, Coad N, Harnden A, Mayon-White R, Perera R, Mant D. How well do vital signs identify children with serious infections in paediatric emergency care? *Arch Dis Child*. 2009;94(11):888–93. doi:10.1136/adc.2009.159095.

51. Paxton LA, Redd SC, Steketee RW, Otieno JO, Nahlen B. An evaluation of clinical indicators for severe paediatric illness. 1996;1990(6):613–618.

52. Weber MW, Mulholland EK, Jaffar S, Troedsson H, Gove S, Greenwood BM. Evaluation of an algorithm for the integrated management of childhood illness in an area with seasonal malaria in the Gambia. *Bull World Health Organ*. 1997;75 Suppl 1(Supplement 1):25–32. Available at: http://www.pubmedcentral.nih.gov/articlerender.fcgi?artid=2486992&tool=pmcentrez&rendertype=abstract.

53. Shann F, Hart K, Thomas D. Acute lower respiratory tract infections in children : possible criteria for selection of patients for antibiotic therapy and hospital admission. 1984;62(5):749–753.

54. Cherian T, John TJ, Simoes E, Steinhoff MC, John M. Evaluation of simple clinical signs for the diagnosis of acute lower respiratory tract infection. *Lancet*. 1988;2(8603):125–8. Available at: http://www.ncbi.nlm.nih.gov/pubmed/2899187. Accessed December 18, 2013.

55. Campbell H, Byass P, Lamont AC, Forgie IM, O'Neill KP, Lloyd-Evans N GB, Campbell H, Byass P, et al. Assessment of clinical criteria for identification of severe acute lower respiratory tract infections in children. *Lancet*. 1989;1(8633):297–9. Available at: http://www.ncbi.nlm.nih.gov/pubmed/2563457. Accessed December 18, 2013.

56. Mulholland EK, Simoes EA, Costales MO, McGrath EJ, Manalac EM, Gove S. Standardized diagnosis of pneumonia in developing countries. *Pediatr Infect Dis J*. 1992;11(2):77–81. Available at: http://www.ncbi.nlm.nih.gov/pubmed/1741202. Accessed December 18, 2013.

57. Simoes EA, McGrath EJ, JI J, Hf H. Recognition of pneumonia by primary health care workers in Swaziland with a simple clinical algorithm. *Lancet*. 1991;340(8834-8835):1502–3. Available at: http://www.ncbi.nlm.nih.gov/pubmed/1361598. Accessed December 18, 2013.

58. Scott JAG, Wonodi C, Moïsi JC, et al. The definition of pneumonia, the assessment of severity, and clinical standardization in the Pneumonia Etiology Research for Child Health study. *Clin Infect Dis*. 2012;54 Suppl 2:S109–16. doi:10.1093/cid/cir1065.

59. World Health Organization. *Acute respiratory infections in children: case management in small hospitals in developing countries. A manual for doctors and other senior health workers.* Geneva Switzerland WHO Programme for the Control of Acute Respiratory Infections 1990.; 1990. Available at: http://www.popline.org/node/384044. Accessed December 19, 2013.

60. Sazawal S, Black RE. Effect of pneumonia case management on mortality in neonates, infants, and preschool children: a meta-analysis of community-based trials. *Lancet Infect Dis*. 2003;3(9):547–556. Available at: http://www.sciencedirect.com/science/article/pii/S1473309903007370. Accessed December 3, 2013.

61. Djelantik IGG, Gessner BD, Sutanto A, et al. Case fatality proportions and predictive factors for mortality among children hospitalized with severe pneumonia in a rural developing country setting. *J Trop Pediatr*. 2003;49(6):327–32. Available at: http://www.ncbi.nlm.nih.gov/pubmed/14725409.

62. Subhi R, Adamson M, Campbell H, Weber M, Smith K, Duke T. The prevalence of hypoxaemia among ill children in developing countries: a systematic review. *Lancet Infect Dis*. 2009;9(4):219–227. doi:10.1016/S1473-3099(09)70071-4.

63. Onyango FE, Steinhoff MC, Wafula EM, Wariua S, Musia J, Kitonyi J. Hypoxaemia in young Kenyan children with acute lower respiratory infection. *BMJ*. 1993;306(6878):612–615.

64. Onyango FE, Steinhoff MC, Wafula EM, Wariua S, Musia J, Kitonyi J. Hypoxaemia in young Kenyan children with acute lower respiratory infection. *BMJ*. 1993;306(6878):612–5. Available at: /pmc/articles/PMC1676956/?report=abstract. Accessed November 28, 2013.

65. Duke T, Subhi R, Peel D, Frey B. Pulse oximetry: technology to reduce child mortality in developing countries. *Ann Trop Paediatr*. 2009;29(3):165–175. doi:10.1179/027249309X12467994190011.

66. Ashraf H, Mahmud R, Alam NH, et al. Randomized controlled trial of day care versus hospital care of severe pneumonia in Bangladesh. *Pediatrics*. 2010;126(4):e807–815. doi:10.1542/peds.2009-3631.

67. Simoes EAF, Roark R, Berman S, Esler LL. Respiratory rate : measurement of variability over time and accuracy at different counting periods. 1991;(4):1199–1203.

68. English M, Murphy S, Mwangi I, Crawley J, Peshu N, Marsh K. Interobserver variation in respiratory signs of severe malaria. *Arch Dis Child*. 1995;72(4):334–6. Available at: http://www.pubmedcentral.nih.gov/articlerender.fcgi?artid=1511241&tool=pmcentrez&rendertype=abstract.

69. UNICEF. Pneumonia Diagnostics : Current Outlook and Perspectives. 2013;(July).

70. Standage S, Wong H. Biomarkers for pediatric sepsis and septic shock. *Expert Rev anti-infective …*. 2011;9(1):71–79. doi:10.1586/eri.10.154.Biomarkers.

71. Galetto-Lacour A, Zamora S., Gervaix A. Bedside Procalcitonin and C-Reactive Protein Tests in Children With Fever Without Localizing Signs of Infection Seen in a Referral Center. *Pediatrics*. 2003;112(5):1054–1060. doi:10.1542/peds.112.5.1054.

72. Van den Bruel A, Thompson MJ, Haj-Hassan T, et al. Diagnostic value of laboratory tests in identifying serious infections in febrile children: systematic review. *BMJ*. 2011;342:d3082. doi:10.1136/bmj.d3082.

73. Carrol ED, Mankhambo LA, Jeffers G, et al. The diagnostic and prognostic accuracy of five markers of serious bacterial infection in Malawian children with signs of severe infection. *PLoS One*. 2009;4(8):e6621. doi:10.1371/journal.pone.0006621.

74. Diez-Padrisa N, Bassat Q, Machevo S, et al. Procalcitonin and C-reactive protein for invasive bacterial pneumonia diagnosis among children in Mozambique, a malaria-endemic area. *PLoS One*. 2010;5(10):e13226. doi:10.1371/journal.pone.0013226.

75. Cheung Y-B, Zaman SMA, Ruopuro M-L, et al. C-reactive protein and procalcitonin in the evaluation of the efficacy of a pneumococcal conjugate vaccine in Gambian children. *Trop Med Int Heal TM IH*. 2008;13(5):603–611. doi:10.1111/j.1365-3156.2008.02050.x.

76. Schuetz P, Briel M, Christ-Crain M, et al. Procalcitonin to guide initiation and duration of antibiotic treatment in acute respiratory infections: an individual patient data meta-analysis. *Clin Infect Dis an Off Publ Infect Dis Soc Am*. 2012;55(5):651–662. doi:10.1093/cid/cis464.

77. Manzano S, Bailey B, Girodias J-B, Galetto-Lacour A, Cousineau J, Delvin E. Impact of procalcitonin on the management of children aged 1 to 36 months presenting with fever without source: a randomized controlled trial. *Am J Emerg Med*. 2010;28(6):647–653. doi:10.1016/j.ajem.2009.02.022.

78. Stocker M, Fontana M, El Helou S, Wegscheider K, Berger TM. Use of procalcitonin-guided decision-making to shorten antibiotic therapy in suspected neonatal early-onset sepsis: prospective randomized intervention trial. *Neonatology*. 2010;97(2):165–174. doi:10.1159/000241296.

79. Esposito S, Tagliabue C, Picciolli I, et al. Procalcitonin measurements for guiding antibiotic treatment in pediatric pneumonia. *Respir Med*. 2011;105(12):1939–1945. doi:10.1016/j.rmed.2011.09.003.

80. Galetto-Lacour A, Zamora S a, Andreola B, et al. Validation of a laboratory risk index score for the identification of severe bacterial infection in children with fever without source. *Arch Dis Child*. 2010;95(12):968–73. doi:10.1136/adc.2009.176800.

81. Lacour AG, Gervaix A, Zamora SA, et al. Procalcitonin, IL-6, IL-8, IL-1 receptor antagonist and C-reactive protein as identificators of serious bacterial infections in children with fever without localising signs. *Eur J Pediatr*. 2001;160(2):95–100.

Available at: http://www.ncbi.nlm.nih.gov/pubmed/11271398. Accessed December 19, 2013.

82. Erdman LK, Dhabangi A, Musoke C, et al. Combinations of host biomarkers predict mortality among Ugandan children with severe malaria: a retrospective case-control study. *PLoS One*. 2011;6(2):e17440. doi:10.1371/journal.pone.0017440.

83. Conroy AL, Glover SJ, Hawkes M, et al. Angiopoietin-2 levels are associated with retinopathy and predict mortality in Malawian children with cerebral malaria: a retrospective case-control study*. *Crit Care Med*. 2012;40(3):952–959. doi:10.1097/CCM.0b013e3182373157.

84. Tayiog TE, Wirima J, Mu RWS, Slutsbr L. In-hospital morbidity and mortality due to malaria-associated severe anaemia in two areas of Malawi with different patterns of malaria infection *. 1994:548–551.

85. Lackritz EM, Campbell CC, Ruebush TK, et al. Effect of blood transfusion on survival among children in a Kenyan hospital. *Lancet*. 1992;340(8818):524–8. Available at: http://www.ncbi.nlm.nih.gov/pubmed/1354285. Accessed December 19, 2013.

86. Chalco JP, Huicho L, Alamo C, Carreazo NY, Bada C a. Accuracy of clinical pallor in the diagnosis of anaemia in children: a meta-analysis. *BMC Pediatr*. 2005;5:46. doi:10.1186/1471-2431-5-46.

87. WHO | Blood sample volumes in child health research: review of safe limits. Available at: http://www.who.int/bulletin/volumes/89/1/10-080010/en/#. Accessed December 19, 2013.

88. Roberts KB. Urinary tract infection: clinical practice guideline for the diagnosis and management of the initial UTI in febrile infants and children 2 to 24 months. *Pediatrics* 2011;**128**:595–610. doi:10.1542/peds.2011-1330

89. Nijman RJM, Radmayr C, Tekgu S. Urinary Tract Infections in Children : EAU / ESPU Guidelines. 2014;**49**:1–13. doi:10.1016/j.eururo.2014.11.007

90. Reali F, Sferrazza Papa GF, Carlucci P, *et al.* Can lung ultrasound replace chest radiography for the diagnosis of pneumonia in hospitalized children? *Respiration* 2014;**88**:112–5. doi:10.1159/000362692

15 Appendices

15.1 Consent form

15.2 Data collection forms
