# Supplementary material for: A novel electronic algorithm using host biomarker point-of-care tests for the management of febrile illnesses in Tanzanian children (e-POCT): A randomized, controlled non-inferiority trial
Source: PLoS Med. 2017 Oct 23;14(10):e1002411. doi: 10.1371/journal.pmed.1002411 (PMC5653205; doi:10.1371/journal.pmed.1002411)
Supplement: S2 Text — (PDF) [file pmed.1002411.s011.pdf]

# Statistical Analysis Plan

**Electronic algorithms based on host biomarkers Point of Care Tests to decide on admission and antibiotic prescription in Tanzanian febrile children (e-POCT)**

## 1 Introduction

### 1.1 Preface

Infections continue to be the main cause of child mortality, especially in resource-poor settings. Health professionals in developing countries have limited ability to identify correctly those children at risk of dying and those in need of antibiotics among children presenting with acute fever to outpatient health facilities. The main reasons for this shortcoming are limited clinical skills and time, unavailability of diagnostic tests (laboratory or X-ray) and non-adherence to practice guidelines. Child mortality is therefore higher than it should be. Another consequence has been the over-prescription of antibiotics resulting in an increase in drug resistance. Emergence of drug resistance is one of the major public health threats worldwide. Approaches to improve management of infections have largely relied on improving the detection of causative pathogens. However, new approaches based on host biomarkers have emerged to help distinguishing between 1) severe and mild infections and 2) invasive disease and mere colonization, regardless of the microorganism involved. Unlike usual clinical signs, whose diagnostic inaccuracy has been largely demonstrated, these objective measurements will facilitate clinician's assessment and hence appropriate management of patients. High costs from incremental pathogen testing are also avoided, which makes such a strategy potentially attractive for implementation at peripheral health facility and community level.

### 1.2 Purpose of the analyses

These analyses will assess the safety of e-POCT in managing children with acute febrile illnesses in comparison with ALMANACH.

## 2 Study Objectives and Endpoints

### 2.1 Study Objectives

## General Objective

The goal is to improve the health outcome of children with acute fever through rapid and accurate identification of those children at increased risk of life-threatening infections and those children that would most likely benefit from antibiotics, through the use of point-of-care technologies appropriate for implementation at primary care level. Through this approach we also aim at a more rational use of antimicrobials, and therefore mitigation of the development of antimicrobial resistance and drug side effects, as well as reduction in of misallocation of scarce health resources that results from inappropriate antibiotic use, inappropriate referral to higher level of care, and unnecessary inpatient treatment

## Specific Objectives

### *Primary Objective*

To compare the clinical outcome of febrile children 2-59 months of age managed using e-POCT (intervention arm), ALMANACH (reference control arm) and routine practice (routine control cohort).

### *Secondary Objectives*

To compare the rational use of antimicrobials in treating febrile children using e-POCT, ALMANACH and routine care.

To compare the performance of e-POCT, ALMANACH and routine care in identifying children at risk for life-threatening infection among febrile children.

To assess the diagnostic performance of new-generation host-biomarkers in identifying children at risk for life-threatening infection and for clinical failure among febrile children (e-POCT and ALMANACH arms).

To measure the proportion of febrile children with hypoxemia, stratified by diagnostic classification (e-POCT arm).

To assess the diagnostic performance of new generation host biomarkers in identifying children in need for antibiotics among febrile children (e-POCT and ALMANACH arms).

## 2.2 Endpoints

## Primary

Proportion of clinical failure by day 7.

| *At any time between initial assessment and day 7:* | *At day 3:* | *At day 7:* |
|---|---|---|
| Coma<br>More than 2 convulsions within 24hr<br>Inability to drink or breastfeed<br>Hypoxemia<br>Severe tachypnea<br>Severe tachycardia | History of cough and tachypnea<br>History of cough and lower chest indrawing<br>Significant dehydration | Fever or temperature ≥38°C<br>History of cough and tachypnea<br>History of cough and lower chest indrawing<br>Diarrhea<br>Significant dehydration<br>Serious skin infection<br>A new significant symptom or sign related to the acute episode but not present at day 0. |

**TABLE 1: DEFINITION OF CLINICAL FAILURE**

## Secondary

- proportions of secondary hospitalization and death by day 30.
- proportions of children prescribed an antibiotic and/or antimalarial treatment at day 0.
- proportion of primarily referred/admitted children compared among the 3 study arms.
- proportions of children with hypoxemia, stratified by diagnostic classification (e-POCT arm)
- performance of combinations of host biomarkers in identifying children at risk for life-threatening infections and for clinical failure among children presenting with fever (e-POCT and ALMANACH arms).

### Exploratory

- Performance of combinations of host biomarkers in identifying children in need for antibiotic treatment, by type of infection (e-POCT and ALMANACH arms).

# 3 Study Methods

## 3.1 General Study Design and Plan

This is a randomized controlled non-inferiority trial investigating a new electronic point-of-care tool (e-POCT) for the management of acute febrile episodes among children 2-59 months of age. Patients will be recruited from the outpatient department of municipal/district government health facilities (health centers, dispensaries, and hospitals) in Dar es Salaam, Tanzania. The study sites will be chosen in collaboration with the CMOH of Dar es Salaam. The city centre of Dar es Salaam has been chosen because of its relative low endemicity for malaria allowing better investigation of febrile episodes caused by other diseases than malaria. The study will include children ≥2 months and <60 months of age who present with acute fever for less than 7 days. Study participants will be randomly assigned to be managed by e-POCT (Arm 1, intervention) or by ALMANACH (Arm 2, reference control). Randomization will be performed at patient level in blocks of four using sealed, opaque case report forms. In parallel, patients attending the OPD of a facility where recruitment for arms 1 and 2 don't take place will be included in the routine care cohort:

All consecutive patients meeting the inclusion criteria will be included. All children will be evaluated at the time of enrolment (day 0) and will have follow-up evaluations on day 3 and 7 (and 14 if not cured at day 7), or at any time in between in case of clinical deterioration according to caretakers evaluation. All patients will also be followed-up on day 30 via a phone interview to ask for secondary hospitalization or death.

Patients from arms 1 and 2 (e-POCT and ALMANACH) presenting with fever without source and severe disease will also be included into a sub-cohort study that aims at investigating new host biomarkers to predict bacterial disease.

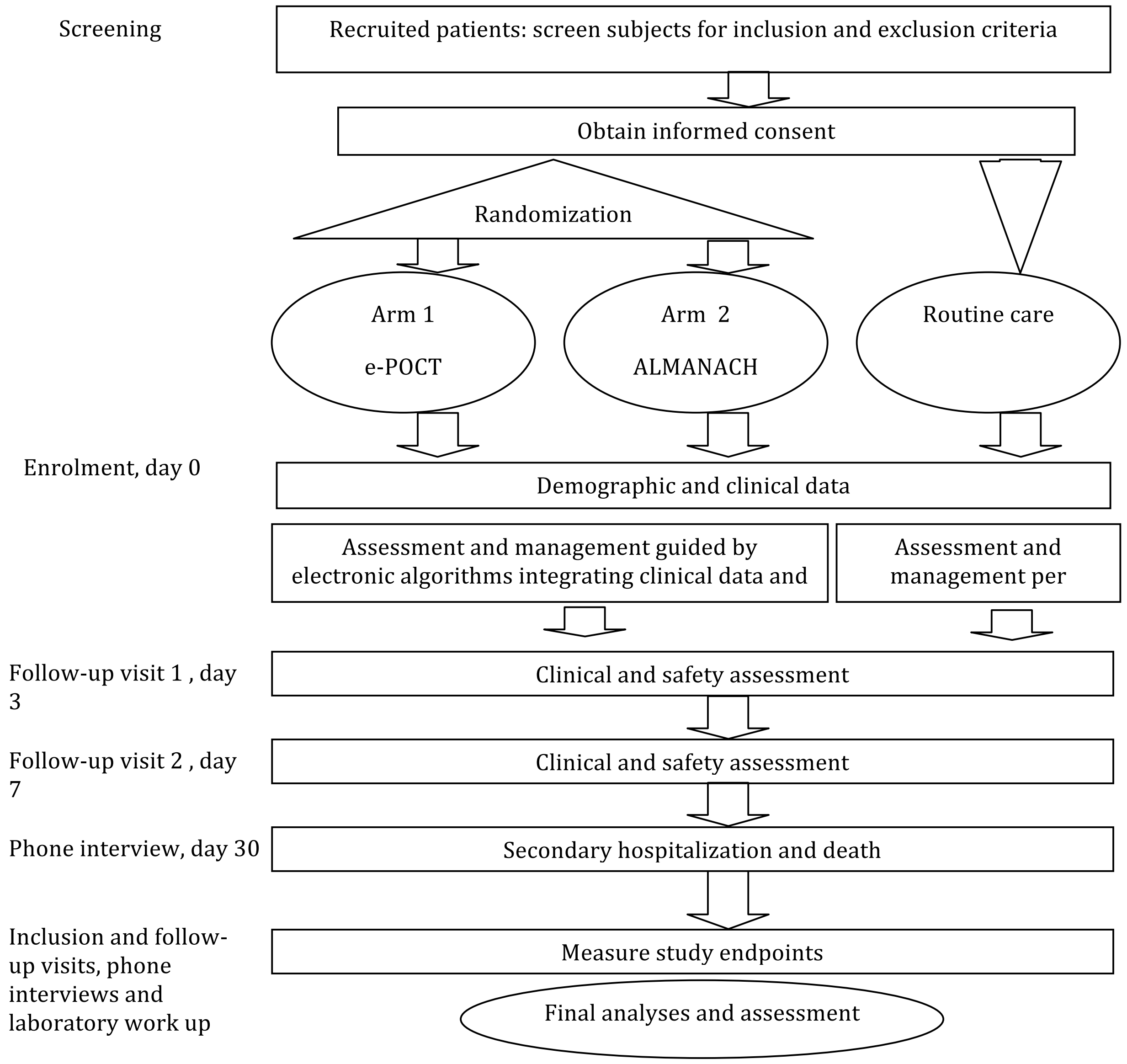
Screening
Recruited patients: screen subjects for inclusion and exclusion criteria
Obtain informed consent
Randomization
Arm 1
e-POCT
Arm 2
ALMANACH
Routine care
Enrolment, day 0
Demographic and clinical data
Assessment and management guided by electronic algorithms integrating clinical data and
Assessment and management per
Follow-up visit 1 , day 3
Clinical and safety assessment
Follow-up visit 2 , day 7
Clinical and safety assessment
Phone interview, day 30
Secondary hospitalization and death
Inclusion and follow-up visits, phone interviews and laboratory work up
Measure study endpoints
Final analyses and assessment

## 3.2 Non-Inferiority Design

A non-inferiority margin of 3% was chosen based on clinical reasoning, i.e. for comparison between arm 1 and 2, non-inferiority will be declared if the upper limit of the one-sided 97.5% confidence interval (CI) for the percent of clinical failure in the investigational group will not exceed a difference of 3% from the percent in the control groups [risk difference=3%], equivalent to a one-sided test with an alpha value of 0.025.

## 3.3 Inclusion-Exclusion Criteria and General Study Population

Patient recruitment takes place in the outpatient department (OPD) of health facilities (health centers, dispensaries, and district hospitals) in Dar Es Salaam. Dar es Salaam was chosen because of its relative low endemicity for malaria allowing better investigation of febrile episodes caused by other diseases than malaria. All consecutive patients presenting to the OPD are considered for enrolment in the study. Study participants are identified at the triage areas of the OPD where axillary and tympanic temperatures are measured. Eligible children are offered enrolment in the study.

***Inclusion criteria***

- Age ≥2 months and <60 months of age
- Written informed consent from the child's parent or caregiver
- Axillary temperature ≥37.5°C
- History of fever for ≤7 days
- First consultation for the current illness
- Live in the catchment area of the health facility

***Exclusion criteria***

- Weight less than 2.5kg
- Chief health problem is an injury, trauma or acute poisoning

The inclusion criteria were chosen to select children with acute febrile episodes (which are different from chronic episodes lasting more than a week). The age range is based on the WHO IMCI reference standard which covers children aged 2 months-5 years.

## 3.4 Randomisation and Blinding

Patients will be enrolled by the study clinicians and then randomized to one of the two management arms (e-POCT or ALMANACH). Randomization will be at patient level in blocks of four according to a computer-generated randomization list provided by an independent, off site researcher. Sealed, opaque forms will be used for allocation concealment and opened only after the patient's enrollment.

## 3.5 Study Variables

| | Baseline (day 0) | Day 3 (or any spontaneous follow-up in between), patient not cured | Day 7 follow-up (if patient not cured | Day 30 follow-up |
|---|---|---|---|---|
| Chief complaints | X | X | X | |
| Past medical history | X | | | |
| Weight | X | X | X | |
| Vital signs | X | X | X | |
| Variables required to compute clinical failure criteria | X | X | X | |
| POCTs per algorithm | X | X | | |
| Rapid typhoid test | | | X | |
| Full clinical assessment | | | X | |
| Blood samples for biomarker analysis | X | X | X | |
| Adverse event monitoring | X | X | X | X |

Assessments collected from enrolment (day 0) before day 2 will be identified as a day 1 visit; from days 2 to 5 as a day 3 visit, and from days 6 to 12 as a day 7 visit. For multiple measurements that occur within the same assessment time window, the assessment closer to the targeted day will be taken into account. The development of severe symptoms will be taken into account at any follow-up day. When a patient was evaluated on day 7 and cured, the development of severe symptoms after day 7 will not be counted as a clinical failure.

# 4 Sample Size

The sample size was calculated based on the primary outcome using the following equation for non-inferiority:

$$\varepsilon = p_1 - p_2$$

$$H_0 : \varepsilon \le \delta \quad vs \quad H_1 : \varepsilon > \delta$$

$$k = n_1 / n_2$$

$$n_2 = \frac{(z_\alpha + z_\beta)^2}{(\delta - \varepsilon)^2}\left((p_1(1-p_1))/(k - p_2(1-p_2))\right)$$

where $p_1$= proportion of clinical failure by day 7 in control arm, $p_2$= proportion of clinical failure by day 7 in intervention arm, α=level of significance, β=1-power, δ=the acceptable difference in proportion of clinical failure between the 2 arms, $n_1$=number of patients in the control arm and $n_2$=number of patients in the intervention arm.

The ePOCT sample (arm1) will be used to make both comparisons between arm 1 and 2 and between 1 and to routine care.

The following assumptions were made for sample size calculation:

ePOCT versus ALMANACH: $p_1$=$p_{2,}$=10%, α=0.025 β=0.2, δ=0.03, $n_1$:$n_2$=1:1.

The proportion of clinical failure by day 7 in control arm ($p_1$) is based on the results of the previous study where children were managed with ALMANACH, taking the sub-group of children having the same inclusion criteria as for the present study.

The clinical outcome by day 7 is expected to be better in the e-POCT arm compared to routine care and therefore a very small sample size is needed to be able to show that e-POCT is not inferior to routine care. The number of patients needed in the routine care arm has thus been estimated to ensure that the distribution of main complaints will not be significantly different between the 2 arms. For that purpose a ratio of 1:3 was chosen.

| | Sample size per arm | Sample size taking into account exclusions, clustering, and drop-outs |
|---|---|---|
| e-POCT (=Arm 1) | 1570 | 1600 |
| ALMANACH (=Arm 2) | 1570 | 1600 |
| Routine care | 533 | 550 |
| **TOTAL** | **2894** | **3750** |

**TABLE 2 SAMPLE SIZE CALCULATION**

# 5 General Considerations

## 5.1 Timing of Analyses
The final analysis will be performed once all subjects have completed the day 30 follow-up.

## 5.2 Analysis Populations

### 5.2.1 Full Analysis Population (Intention to Treat)
- All subjects randomized or recruited into the routine care cohort.

### 5.2.2 Per Protocol Population
- *All subjects who received the day 0 intervention/ are managed by the routine clinician at day 0 and complete the day 7 follow-up.*

The primary non-inferiority analysis will be based on the per protocol population. The analysis for the secondary outcomes will be done in the per protocol population unless the difference between the full and per protocol population is more than 1% and/or there are substantial differences between the study arms.

## 5.3 Covariates and Subgroups
The influence of covariates on the primary outcome will be assessed using logistic regression modelling and will include: health-center, clinician, demographics, and vital signs.

Subgroup analyses for patients with severe disease, respiratory symptoms, and fever without source will be performed depending on the number of subjects enrolled in these subgroups and the attainable power.

## 5.4 Missing Data
Based on experience from previous studies we expect few missing data for the primary and secondary outcome. Generally, it will be assumed that all patients lost to follow-up for the primary and secondary outcomes experienced the (negative) outcome.

## 5.5 Interim Analyses and Data Monitoring
An independent data safety and monitoring board will be established before inclusion of the first patient. Interim analyses will be performed after inclusion of 100 and 500 patients in each of the intervention and reference control arms.

*Stopping rules:*

The absolute difference in the clinical failure rate by day 7 should be no more than 5%, otherwise the study will be discontinued. The stopping rules are based on an alpha of 0.025 for a one-sided confidence interval. The calculations below are based on clinical failure rates of 5% and 10% in the control ALMANACH arm.

| number of patients per arms 1 and 2 | number of failures expected in ALMANACH arm | number of failures in e-POCT arm if 5% worse than ALMANACH (acceptable) | upper CI bound for 5% more failures: STOP if more than this number |
|---|---|---|---|
| *ALMANACH failure rate = 5%, e-POCT not worse than 10%* | | | |
| 100 | 5 | 10 | 40 |
| 500 | 25 | 50 | 81 |
| *ALMANACH failure rate= 10%, e-POCT not worse than 15%* | | | |
| 100 | 10 | 15 | 52 |
| 500 | 50 | 75 | 114 |

# 6 Statistical methods and tests, main tables and figures

A basic flowchart of the study will detail patients screened, patients randomized/enrolled, patients who received intervention, patients lost two follow-up. The ITT and PP populations will be specified.

Median follow-up time will be calculated per study arm.

| | Day 3 (median, IQR) | Day 7 (median, IQR) | Day 30 (median, IQR) |
|---|---|---|---|
| ePOCT | *pCRF3/ QC log* | *pCRF3/ QC log* | *pCRF3/ QC log* |
| ALMANACH | *pCRF3/ QC log* | *pCRF3/ QC log* | *pCRF3/ QC log* |
| routine | *pCRF3/ QC log* | *pCRF3/ QC log* | *pCRF3/ QC log* |

Table X: median follow-up time per arm

Baseline characteristics (demographics, vital signs, chief complaint) will be summarized according to the study arm.

| | ePOCT | ALMANACH | Routine |
|---|---|---|---|
| Age in months | *pRCF3, ePOCT* | *pCRF3, ALMANACH* | *pCRF3, eCRF7* |
| Chief complaint (n,%) | *pCRF3,eCRF8* | *pCRF3, eCRF8* | *pCRF3, eCRF8* |
| Underlying medical issues | *pCRF3,eCRF8* | *pCRF3,eCRF8* | *pCRF3, eCRF8* |
| Maternal education<br>-none<br>-primary<br>-post-primary<br>-post-secondary | *eCRF8* | *eCRF8* | *eCRF8* |
| Weight for age z-score | *pCRF3/epoct* | *pCRF3, ALMANACH* | *pCRF3, eCRF8* |
| Vital signs | *pCRF3/epoct* | *pCRF3, ALMANACH* | *pCRF3, eCRF8* |
| HIV status (children tested, children HIV pos) | *Epoct/eCRF09* | *eCRF09, eCRF08* | *eCRF09, eCRF8* |

| | | | |
|---|---|---|---|
| vaccinations not up to date | *eCRF10* | *eCRF10* | *eCRF10* |
| Received PCV vaccine | *eCRF10* | *eCRF10* | *eCRF10* |

Table X: baseline characteristics

For the primary outcome the proportion of clinical failure by day 7 will be compared between arm 1 and 2 and arm 1 and routine care. Non-inferiority will be declared if the upper limit of the one-sided 97.5% confidence interval (CI) for the percent of clinical failure in the investigational group will not exceed a difference of 3% from the percent in the control grou, ALMANACH, (risk difference=3%), equivalent to a one-sided test with an alpha value of 0.025.

For the secondary outcomes the risk of the following measures will be compared; risk ratios and risk differences will be calculated:

- Antibiotic prescription at day 0 (and by day 7)
- Severe adverse events (secondary hospitalization and deaths) by day 30
- Primary admissions (referrals)

| | ePOCT | ALMANACH/ routine |
|---|---|---|
| Clinical failure by day 7 | *pRCF3, ePOCT* | *pCRF3, ALMANACH* |
| Antibiotic prescription | *pCRF3,eCRF8* | *pCRF3, eCRF8* |
| Primary referrals/admission | *pCRF3,eCRF8* | *pCRF3,eCRF8* |
| Severe adverse events | *pCRF3,eCRF8* | *pCRF3,eCRF8* |
