# Supplementary material for: A novel electronic algorithm using host biomarker point-of-care tests for the management of febrile illnesses in Tanzanian children (e-POCT): A randomized, controlled non-inferiority trial
Source: PLoS Med. 2017 Oct 23;14(10):e1002411. doi: 10.1371/journal.pmed.1002411 (PMC5653205; doi:10.1371/journal.pmed.1002411)
Supplement: S3 Text — (DOCX) [file pmed.1002411.s012.docx]

**S3 Text: Detailed description and discussion of the methods and evidence used for the development of the e-POCT algorithm.**

1. **Methods**
   1. **Structured Literature Review**

We undertook structured literature searches to review publications on the key elements that required update from ALMANACH and IMCI. We focused on the following areas in an ambulatory care setting:

1. Identification of children with severe infections using clinical signs and/or available POC laboratory tests.
2. Management of children with respiratory infections, including identification of children with severe respiratory infections and those in need for antibiotic treatment.
3. The use of clinical signs and available POCTs to decide on antibiotic prescriptions for children with fever without focal symptoms (FWS).
4. Symptoms and signs for dehydration.
5. Diagnosis of severe malnutrition

We constructed three separate searches for questions 1-3, 4, and 5 (S1B Table) and merged the resulting records.

Papers reviewed were in English, and published between inception to June 2014 (which was when the construction of e-POCT was completed). We also checked publications since June 2014 for relevant updates to the retrieved evidence. Databases searched were PubMed and Embase. The literature search was supplemented by hand-checking references of filtered papers, and by snowball search of key papers in Google Scholar. Studies involving only infants below 3 months of age or only adults were excluded. Systematic reviews addressing the questions of interest were also considered.

- 1. **Algorithm construction**

ALMANACH, an electronic algorithm derived from IMCI, was used as a starting point for construction of the new algorithm. We used the evidence retrieved from the literature search to propose modifications when relevant. Diagnostic criteria to be included should not only have adequate accuracy at primary care level but also be assessable by health workers in resource-poor settings. A new decision tree, e-POCT was constructed including a detailed reference paper version that was used to program the algorithm onto an android-based electronic support tool using the Mangologic™ software (http://www.mangologic.com/). Each branch of the electronic algorithm was validated against a standardized set of theoretical patient scenarios. A final version was piloted during a pilot phase in primary care facilities in Dar es Salaam, Tanzania, that included 100 pediatric consultations, which were directly supervised by a medical doctor or pediatrician.

1. **Evidence retrieved and rationale for e-POCT algorithm construction**

S1C Fig displays the retrieved and included publications.

- 1. **Identification of children with severe infections: how can current referral criteria be improved?**
     1. **What is the evidence for currently employed IMCI danger signs?**

The 2014 IMCI algorithm recommends immediate hospital transfer in the presence of any one of the general or disease specific danger signs (Table 1, main text). These danger signs were chosen based on expert opinion and refined during the initial IMCI development studies [8,19,20]. They were then evaluated in two studies conducted in hospital outpatient departments in Kenya and Bangladesh between 1993 and 1995 [21,22]. These studies determined the accuracy of the IMCI danger signs to predict hospitalization when compared to clinical judgment of a medical doctor or clinical officer. The Kenyan (2799 children, 779 admissions) study found a sensitivity and specificity of 46% and 80%, respectively.—a similar range compared to what was observed in the initial development studies [19,20]. Stiff neck, stridor, corneal ulceration, and tender swelling behind the ear were detected in none of the children. The Bangladeshi study (668 children, 226 admissions) reported a sensitivity of 86% and a specificity of 64% [22]. The heterogeneity of reported accuracy is probably related to differences in the studied population and in the outcome assessment. Since the overall performance of IMCI severity criteria was low, we sought to identify additional studies that evaluated and complemented single criteria. In order to identify criteria with higher performance that would be implementable in primary care facilities in low resources settings, we looked at pediatric triage tools, severity scores predicting mortality, and diagnostic tools to identify serious infections.

- - 1. **Should additional signs from pediatric triage tools be considered?**

Several triage tools exist that are design to help prioritize treatment of sick children at hospital level. Two scales have been tailored to low resource environments: the WHO Emergency Triage and Treatment Tool (ETAT) [23], and the Pediatric South African Triage Scale (PSATS) [24]. However, only ETAT has been evaluated in a low-income country (Malawi) [25]. The ETAT guidelines rely on clinical discriminators using an ABCD concept (Airway, Breathing, Circulation/Coma/Convulsion, Dehydration) [26].

- - 1. **Should we include criteria from available pediatric severity scores?**

In well-resourced countries, a number of pediatric severity scores have been developed such as the Pediatric Risk of Mortality (PRISM) score [27,28], the Pediatric Index of Mortality (PIM) [29], the Pediatric Early Warning System (PEWS) [30], Bedside PEWS [31], and Pediatric Advanced Warning Score (PAWS) [32]. Besides PAWS, these scores are neither designed for use in outpatient nor resource-poor settings and rely on complex clinical assessments and laboratory data. Rather, the scores were designed to predict inpatient death. The PAWS score uses a full set of vital signs (respiratory rate [RR], heart rate [HR], oxygen saturation [SaO2], blood pressure [BP], body temperature [T]) and work of breathing, inspirated O2, CRT, and the Alert, Voice, Pain, Unresponsive (AVPU) scale. When validated among 49 pediatric patients admitted from the emergency department of a tertiary care hospital to the intensive care unit versus 46 controls admitted to the regular pediatric floor, it’s accuracy was moderate: the sensitivity and specificity were 83% and 65% for of a score ≥2 and 70% and 90% for a score ≥3 [32]. For comparison, a child with fever and chest indrawing and no other criteria would have a PAWS score of at least 4.

Similarly, general and disease-specific risk scores have been developed in resource-limited settings that aim at predicting death among hospitalized patients [33–36]. The Signs of Inflammation in Children that Kill (SICK), a score to predict in-hospital mortality, was derived from 1,099 (44 deaths) children in a tertiary care setting and validated among 125 children in the same setting (23 deaths), as well as 3,895 (58 deaths) children in another Indian hospital and 1,473 children (5 deaths) in a UK hospital [37–39]. Second, the Lambaréné Organ Dysfunction Score (LODS), a tool to predict death among children hospitalized with malaria, was developed based on data from 23,809 hospitalized children in Africa (1,004 deaths) [40]. A study among 8,091 (436 deaths) hospitalized children in Kenya identified predictors for immediate, early, and late death (PEDIA) [35,41]. We did not identify additional signs that could be assessed by health workers at primary care level from these scores. One sign of PEDIA, jaundice, was also included in ALMANACH as an additional danger sign. This was because jaundice was a good predictor for documented bacterial disease (LR+ 2.9), in particular typhoid (LR+ 6.2), and against documented viral disease (LR+ 0.11) in a study on fever etiologies in 1005 children in Tanzania [4,42] and, because it was hypothesized that primary health care workers could detect clinical jaundice [18]. Jaundice was, however, detected in none of the children in the ALMANACH arm during the initial evaluation studies (Shao, personal communication).

- - 1. **What evidence exists from studies on clinical signs to identify children with serious infections?**

The body of evidence has been growing for the use of clinical signs to identify children with serious infections (essentially infections that require hospital-based treatment) in outpatient high-resource settings. A systematic review of 30 studies assessed the performance of clinical signs in predicting serious infections [14,43]. Likelihood ratios (LRs) were constructed to represent the ability of single predictors to rule in (positive likelihood ratio [LR+]>5) and rule-out (negative likelihood ratio [LR-]<0.2) serious infections. The following predictors of signs that would be measurable by low-level health workers had rule-in value in settings with low-prevalence of serious infections: cyanosis (LR+ 52.2), poor peripheral perfusion (LR + 38.8), shortness of breath (LR+ 9.3), rapid breathing (LR+ 9.78), meningeal irritation (LR +25.7), seizures (LR+ 20.7), and unconsciousness (LR+ 19.8). These findings are in line with findings from studies on predictors of mortality in low-resource settings [33,35,37,40,44,45]. Most of these estimates were based on a prospective derivation study conducted among 3981 children (31 admissions for serious infection) in Belgium [46]. From this study a multivariable triage instrument to predict serious infections requiring hospitalization was constructed using classification and regression tree (CART) analyses. The tool consisted of the clinician’s feeling “that something is wrong”, dyspnea, temperature, age, and presence of diarrhea. It had a sensitivity of 96.8% at a specificity of 88.5%. The triage tool was assessed in another study including 8962 acute illness episodes (283 serious infections) [46]. Sensitivity was 100% at a specificity of 83.6% in the general practitioner setting with 17% of children testing positive. Thompson et al. assessed the diagnostic performance of vital signs in distinguishing 313 children with serious infections from 339 patients mild infections and 48 children with no infections in one outpatient department in England [47]. The LR+ and LR- were as follows: temperature ≥39°C 2.1 and 0.8, tachypnea 1.3 and 0.8, tachycardia 1.5 and 0.7, prolonged CRT 17.7 and 0.9, SaO2≤94% 2.7 and 0.9. Advanced Pediatric Life Support (APLS) cut-offs were used for definition of tachypnea and tachycardia. Having a derangement in any of the vital categories had a LR+ of 1.3 at a LR- of 0.5.

- - 1. **Criteria from triage-, severity- and serious infection-scores integrated into e-POCT**

All current general IMCI danger signs were kept in the e-POCT algorithm. This was because all have high LR+. i.e. value as “red flags” [47]. However, they lack sensitivity and we hence added additional criteria. From ETAT, we included oximetry, and severe respiratory distress into the e-POCT algorithm (see severe pneumonia section below). The circulatory signs were not included since the reliability of CRT and ‘weak pulse’, when measured by low-level health workers at primary care level, have not been assessed sufficiently [48]. Cyanosis, which was added into ALMANACH, was not included into the e-POCT algorithm since we integrated oximetry. From the single criteria of the PAWS score and the diagnostic accuracy studies for detection of serious infections, severe tachycardia was added to the e-POCT algorithm to represent “poor peripheral perfusion”. Tachycardia is indeed a well-recognized criterion for septic shock in children [49]. Given that an oximeter was included into e-POCT (see below), the HR could be fed into the algorithm without requiring additional measurements by the health worker. Like for RR cutoffs (see below), we chose age-and temperature-based percentiles for HR as opposed to APLS-based cutoffs. The electronic algorithm format allowed integration of these more complex calculations. As shown above, APLS thresholds for HR had clinically irrelevant diagnostic accuracy [47]. HR and RR vary with age but also with temperature [50–55]. Recently compiled age centile charts were in large disagreement with previously publishes reference ranges, including APLS [50]. In the study by Thompson et al., the diagnostic performance for severe tachycardia improved drastically if age-and temperature-based percentiles were used: a HR exceeding the 90th centile was highly specific (88%), though not sensitive (24%), for serious infection compared to minor infection [47]. Again, CRT and cyanosis were not included from the severity- and serious infection-scores for the reasons stated above. Dyspnea and tachypnea were addressed in the pneumonia-section of the algorithm (see below). We did not consider the Belgian triage tool since the physician’s statement that “something is wrong” was paramount for the tree [46]. The physician’s general impression is likely strongly correlated to the degree of training. Consequently, we hypothesized that it would not be useful to integrate the Belgian triage tool into the assessment of general danger signs for low-level health workers.

- - 1. **Should hemoglobin screening be included?**

Systematic hemoglobin screening was integrated for all children into e-POCT based on the following rationale: First, severe anemia is an important cause of mortality. In a systematic review, Brabin et al. estimated the relative risk of in-hospital mortality for children with severe anemia at around twice that of less anemic children [56]. Subsequent studies have confirmed that anemia is an important predictor for inpatient death [40,57]. Second, severe anemia is associated with bacteremia in febrile children [42,58]. Third, anemia cannot be detected based on clinical signs and at-risk groups cannot be clearly defined. In a meta-analysis, Chalco et al. found a low sensitivity for conjunctival and palmar pallor in detecting severe anemia [59]. The pooled sensitivity and specificity of conjunctival pallor and palmar pallor were 43.6 (95% CI 41.7–45.6) and 81.4 (95% CI 78.6–83.9), respectively, and 39.2 (95% CI 37.4–41.1) and 86.7 (95% CI 84.6–88.5) [59]. Two studies identified since the meta-analysis confirmed these findings [60,61]. On the other hand, receiving transfusion early improves survival in children with severe anemia [62]. Children with severe anemia should be referred to higher-level facilities for monitoring of signs that indicate the immediate need for blood transfusion)[62,63]. POC hemoglobinometer are already in us for other indications in peripheral, resource-limited settings.

- 1. **How can the management of children with respiratory illness be improved?**

Based on the results from a recent modeling effort, our priority was to integrate a diagnostic test for bacterial pneumonia and a test for severe pneumonia. In this model, the benefits of these test would also largely depend on the infrastructure required and the resulting level of access to the test, i.e. the test should be performable at peripheral care level and results should be obtainable within one hour*.* The targeted sensitivity and specificity of the tests from the model was 95% and 85%, respectively, for the bacterial pneumonia test and 85% and 90% for the severe pneumonia test [64].

- - 1. **Severe pneumonia**

IMCI Severe Pneumonia classification is given in the presence of any danger signs or stridor in a calm child [9]. Before 2014, children with cough and lower chest indrawing were also classified as severe pneumonia [65]. This sign was removed subsequently, as a result of a series of clinical studies [66–73]**,** referred to henceforth as “severe pneumonia trials”. In these studies, intravenous antibiotic treatment and/or hospital-based treatment were not found to be beneficial in children with severe pneumonia based on chest indrawing alone. However, they have several limitations raising the question whether chest indrawing should truly be disregarded as a sign for severe pneumonia. First, like in the early IMCI pneumonia studies, prevalence of chest indrawing was considerably higher in children in the South Asian studies compared to African studies [21,22]. Whether this reflects differences in diagnosis or epidemiology is not clear. Second, case-fatality rate for children included was low (<1%) though the case-fatality rate for severe pneumonia globally has been estimated at 7.2% and others have reported 18% [74,75]. Third, the presence of hypoxemia was not reported. Fourth, it is unclear whether hospital-based supportive interventions, such as provision of oxygen or rehydration, were provided adequately in the control arms. For respiratory infections especially, the need for antibiotic treatment does not necessarily overlap with the need for hospital-based supportive care. Children with bronchiolitis, for example, do not require antibiotic treatment at all but may require respiratory support or rehydration therapy. It is hence essential to identify children in need for hospital-based supportive care, regardless of the microbiological etiology. This was reflected in results from a hospital-based cohort study in Kenya that included 2803 children with respiratory infections. The prevalence of invasive bacterial infection with severe pneumonia syndrome was similar to that with mild pneumonia syndrome, but case fatality was greater among children with severe pneumonia syndrome [45]. Among 310 children with pneumonia (cough and chest indrawing), two viruses causing bronchiolitis (*Respiratory Syntical Virus* and human *Metapneumovirus*) were associated with treatment failure for pneumonia [76].

Since the introduction of IMCI, several severity scores for bronchiolitis [77,78] and asthma [79–82] have been developed in high-resource settings with varying degrees of quality and validation [83]. The Respiratory Index of Severity in Children (RISC) score and its modified versions were developed to predict in-hospital death in South Africa [84], Kenya [85] and Malawi [86]. From the highest quality asthma and bronchiolitis scores, as well as studies in low-income settings that aimed at identifying children with hypoxemia and those at risk for adverse outcome from respiratory infections [34,45,57,75,84,87–92], we identified the following clinical criteria that could be used by low-level health workers: respiratory rate (RR), accessory breathing muscle use (lower chest indrawing), vocalization (short phrases/weak cry, unable to speak/cry), grunting, HR, inability to drink, malnutrition, severe anemia, and level of consciousness [83]. Inability to drink, level of consciousness, malnutrition, severe anemia, and HR were integrated into the general danger signs (see above). We then constructed the “severe respiratory distress” classification as presence of cough and two of the following signs: severe tachypnea (very fast breathing), chest indrawing, decreased vocalization (speaks in short sentences or unable to speak/cry), or grunting. We used a combination of two signs to increase the specificity of detecting children that may require hospital-based supportive care, as suggested in previous studies [75]. For example, children with chest indrawing alone could be safely managed with home-based treatment as in “severe pneumonia trials“(see above). Fast breathing alone was not found to be related to asthma severity [93]. However, fast breathing in addition to chest indrawing has been identified as a predictor for treatment failure among children with WHO severe pneumonia in the “severe pneumonia trials” and other observational studies [67,89,94,95]. Similarly lower chest indrawing in combination with grunting was suggested as a criterion for hospitalization for children with WHO severe pneumonia [75]. RR is known to vary with age and also with temperature in pediatric patients. Consequently, like for HR, we used age and temperature-corrected respiratory centile cutoffs to define tachypnea and severe tachypnea [51], Table 1 (main text). The 97^th^ %ile provided the best cutoff for severe tachypnea in terms of sensitivity and specificity when applying it to the “fever study dataset” [4]. We also included hypoxemia as a criterion for severe pneumonia. Hypoxemia was proposed in the 2014 IMCI chart booklet but has not been implemented [9]. Though it is still unclear at what level of care oximeters are most useful (health center versus hospital-based triage), we did include oximetry into e-POCT to be able to assess its utility at peripheral health care level.

In the era of *Haemophilus influenza type B* (Hib) vaccine, bacterial epiglottitis causing stridor has become very rare. For croup, a viral upper respiratory infection, stridor alone is not sufficient for severe classifications [96,97]. Most children with stridor and severe disease also have other signs of respiratory distress, such as lower chest indrawing [75]. Finally, stridor is difficult to recognize for low-level health workers [20,98] and rare [75](Shao, personal communication). Stridor was therefore omitted in the ePOCT algorithm as a sign for severe pneumonia.

- - 1. **Non-severe pneumonia**

For the “bacterial pneumonia” test, i.e. a test that would identify children in need for antibiotic treatment among children with non-severe respiratory symptoms, we developed a two-step approach of a clinical ‘rule-out-step’ with high sensitivity (fast breathing and/ or chest indrawing) and a subsequent biomarker ‘rule-in-step’ with high specificity. This two-step approach was chosen due to the low prevalence of bacterial pneumonia at peripheral health care level, and the high frequency of respiratory complaints. Using a sensitive, clinical criterion as a first step would select children with a relevant probability of having bacterial pneumonia and therefore i) increase the positive predictive value of the biomarker and ii) avoid unnecessary testing of children at very low risk of having bacterial pneumonia.

Rambaud-Alhaus et al. recently reviewed clinical predictors for radiographic pneumonia and found that they all had very low accuracy, including age-related fast breathing (pooled positive and negative likelihood ratio 1,55 and 0,63) [99]. However, age-related fast breathing was the most sensitive predictor among all studied. Some of the lack in diagnostic accuracy of RR may be related to its variation over time (in the order of 10 counts within one hour)[55,100] and between observers [101]. RR decreases with age until adolescence with the steepest decline apparent in infants during the first 2 years of life [50]. It also increases with body temperature [51,53,54]. Using arbitrary simple binary cutoffs probably oversimplifies the complex relationship between RR and pneumonia [102]. We thus used the 75^th^ percentile of RR for age and temperature derived by Nijman et el. as a cutoff for tachypnea (fast breathing) [51]. We added lower chest indrawing as another criterion to further increase sensitivity. This identified 94% (29/31) of children with radiographic pneumonia in the ‘Fever Study’ dataset [4].

We found evidence that CRP would be helpful in identifying children with bacterial pneumonia. First, there is evidence from pneumococcal vaccine trials that a CRP cutoff of 140 mg/L was shown to identify pneumococcal pneumonia in children with changes seen on chest radiograph [103]. Moreover, in a study among hospitalized children in Mozambique CRP values were significantly higher in 89 children with bacterial pneumonia (positive blood culture) compared to 87 children with viral pneumonia [104]. In a systematic review that included 8 studies the diagnostic accuracy of CRP for pneumonia was moderate overall, with sensitivities ranging from 35%-100% at specificities from 40-100% [100]. CRP concentrations exceeding 35–60 mg/L occurred significantly more often in children with bacterial pneumonia. However, the outcome definitions for pneumonia were very heterogeneous, including clinical, radiological, and microbiological feature. This reflects an overall major limitation of studies assessing the accuracy of biomarkers for pneumonia: an acceptable gold standard remains yet to be developed [107]. Lynch et al. reviewed systematically the diagnosis of pneumonia and found that authors used 11 different gold standards for 25 of the studies included [107]. Radiographically defined pneumonia is often used as a proxy for bacterial pneumonia since it is known that they are correlated: Hib and pneumococcal conjugate vaccines (PCV) reduced radiographic pneumonia by circa 20% in vaccine trials [108–110]. However, reliable data is missing on the true proportion of bacterial pneumonia among cases with chest X-ray (CXR) consolidation [107]. Furthermore, studies included into the systematic review contained different populations, including populations with a high prevalence of malnutrition [111], Malnourished children have distinct inflammatory responses to infection [112]. The review by Flood et al. was further limited by different CRP cutoffs used (ranging from 35-60mg/L)[105].

Since these reviews, Koster et al. published data on the accuracy of CRP in predicting CXR consolidation in 286 children in a Dutch emergency department. Cutoffs of 20, 50, 75, and 100 mg/L yielded sensitivities of 81%, 55%, 51%, and 32%, respectively, at specificities of 53%, 78%, 87%, and 93% [113]. In the Tanzanian ‘Fever Study’, using CART analysis, a combination of CRP and Chitinase 3-like-1 discriminated between end-point pneumonia (n=30) and non-end-point pneumonia (n=125) among children with cough and fast breathing with 93% sensitivity and 81% specificity [114]. A CRP cutoff point of >44 mg/L had a LR+ of 3.8 and a LR- of 0.25 and had the strongest association with end-point pneumonia among all biomarkers studied. Galetto-Lacour et al. found that CRP (≥100 mg/L) had a LR+ of 2.33 and a LR- of 0.13 in differentiating 37 patients with presumed pneumococcal pneumonia from 38 patients with non-pneumococcal pneumonia [115]. In a hospital-based study in 119 children in Italy, CRP values above 49 mg/L were correlated with CXR consolidation and pleural effusion [116]. In another hospital-based study that aimed at establishing a prediction model to differentiate bacterial pneumonia (CXR consolidation and positive microbiological testing for typical and/or atypical bacteria) from viral pneumonia (CXR consolidation and positive viral PCR), CRP>80mg/L was the most significant predictor for bacterial pneumonia [117]. An analysis among 124 children with clinical signs of pneumonia and radiographic changes found that a CRP >80mg/L and >40mg/L had a LR+ of 6.4 and 5.1, and a LR- of 0.38 and 0.32 in detecting 50 children with endpoint pneumonia (Alcoba G, personal communication). For identifying 24 children with complicated pneumonia (pleural effusion and/or bacteremia), the LR+ and LR- for a CRP >80mg/L and >40mg/L were 2.67 and 1.69, and 0.11 and 0.17, respectively. In a multicenter study in the United States including children with pneumonia, bacteremic patients had higher median CRP values (200mg/L, interquartile range 90-320), compared to those with negative blood culture (median 67mg/L, interquartile range 24-190) [118]. A study in a tertiary care center in India found that a CRP cutoff of 60mg/L 93% sensitive and 64% specific in differentiating 28 patients with WHO very severe pneumonia from 22 with WHO severe pneumonia-all had CXR endpoint consolidation [119].

Several studies have addressed the accuracy of PCT differentiating bacterial and viral pneumonia in children[115,120–128]. Results are conflicting: some report an advantage over CRP [122,128] and others don’t [115,120]. CRP outperformed PCT in predicting end-point pneumonia in the ‘Tanzanian fever study’ [114]. No added value was found in a study that combined the two biomarkers over using them alone [120]. Given that i) there is no clear benefit in terms of diagnostic accuracy of using PCT over CRP, ii) the availability of a semi-quantitative lateral-flow rapid test and iii) the lower blood volume required to perform CRP versus PCT testing, we decided to use CRP alone as a second step for the diagnosis of bacterial pneumonia, using a relatively high cutoff in the 60-100mg/L range to ensure high specificity. Given the semi-quantitative cutoffs provided by the manufacturer of the test used for the evaluation of e-POCT in a clinical trial (Bionexia™, Biomerieux), we chose 80mg/L as a cut-off.

In sum, children with cough and tachypnea (RR above or equal to the 75^th^ percentile age and temperature) and/or chest indrawing underwent CRP-testing. Children with CRP values equal or above 80mg/L were classified as bacterial pneumonia and antibiotic treatment was recommended, except for children with malaria since malaria alone raises CRP values [104]. The relatively high cutoff of 80mg/L was chosen to guarantee high rule-in value at the primary care level and to avoid unnecessary antibiotic treatment.

- 1. **How can we improve diagnosis of serious bacterial infections among children with fever without source?**

When the probable cause of a febrile illness cannot be determined by history or physical examination, the illness is labeled as fever without source (FWS, alternatively fever without localizing signs, fever without a focus or undifferentiated fever). Though the majority of children with FWS have self-limiting viral diseases, a small proportion will have bacterial infections; the most common are urinary tract infection (UTI), occult bacteremia, and occult pneumonia. Before the introduction of Hib and PCV, 3-4% of children aged 3-24 months with FWS had occult bacteremia in outpatients studies in the United States, the majority due to Hib and *Pneumococcus* [129,130]. In studies conducted in Europe and United States, the overall bacteremia rate for children 3-36 months dropped to <0.5% in settings with high PCV coverage [131,132]. UTIs occur in approximately 7% of children with FWS [133,134]. To address UTI, urinary dipstick testing was introduced into ALMANACH [18]. A meta-analysis reported a pooled LR+ of 6.1 (4.3, 8.6) and LR- of 0.20 (0.16, 0.26) for a positive urine dipstick in diagnosing a positive urine culture [135]. The test accuracy in children younger than 2 years is lower compared to older children [136]. Obtaining clean urinary samples is however challenging in small children, i.e. those at highest risk for urinary tract infection [134]. In the tropics, malaria is an important cause of FWS, which can be readily diagnosed by mRDT [4]. For bacterial infections, typhoid fever, rickettsial diseases, scrub typhus, and leptospirosis cause FWS, with seasonal and geographic variation. ALMANACH included abdominal tenderness as a predictor for typhoid disease [18]. However, assessment of abdominal tenderness requires advanced pediatric clinical skills. It was detected in none of the 842 patients assessed in the ALMANACH study (Shao, personal communication). In studies using methodologies without gold standard diagnosis, available pathogen-specific POCTs for typhoid have low accuracy with sensitivities of approximately 60% and specificities of approximately 80% [137].

In order to improve identification of children in need for antibiotic treatment among those with FWS, we first evaluated clinical signs. Van den Bruel et al. systematically reviewed clinical signs for identifying children with serious bacterial infections [43]. No additional clinical signs could be identified from this review. More recently, De Santis *et al.* evaluated clinical predictors of bacterial disease in the Tanzanian fever study. In addition to the signs that were already included in the severe disease classification (see above), low weight (weight for age [WFA] <2 standard deviations [138], adjusted LR+ 4.6], cervical, axillary or inguinal lymphadenopathy (adjusted LR+ 3.5) and fever duration of >3 days (adjusted LR+ 1.6) were identified [42]. Abdominal tenderness was a predictor of typhoid (adjusted LR+ 7.0). However, we did not keep “abdominal tenderness” due to its unproven reliability (see above).

Next, we assessed whether CRP or PCT (which are available as POCT), or a combination of both biomarkers would be helpful in detecting children in need for antibiotic prescription among children with FWS. Van den Bruel et al. summarized the diagnostic value of laboratory tests in identifying serious infections in children (including children with FWS) in a systematic review [17]. The best performing biomarkers were PCT (LR+ 1.75 to 3.11; LR- 0.08 to 0.35) and CRP (LR+ 2.40 to 3.79; LR- 0.25 to 0.61). An earlier review focusing on CRP only found similar results [106]. For UTI specifically, we identified 6 and 13 studies that addressed the diagnostic accuracy of PCT and CRP respectively for the diagnosis of pyelonephritis (confirmed by DMSA scan) in children. All studies were summarized in a recent systematic Cochrane review [139]: PCT, at a cutoff of 0.5 ng/mL, and CRP, at a cutoff of 20mg/L had a sensitivity of 86% and 94% and a specificity of 74% and 39% respectively. Test accuracies for higher cutoffs were not reported. We found two relevant studies that assessed the diagnostic accuracy of CRP in detecting invasive *Salmonella* infection. In the first study, a CRP cutoff of 35mg/L was found to be 55% sensitive at a specificity of 88% [140]. The accuracy was lower when clinical and/or microbiological confirmation were used in other studies [141,142]. We could not identify relevant studies on the diagnostic accuracy of CRP or PCT for leptospirosis, rickettsial disease nor scrub typhus.

The overall evidence of retrieved studies was not sufficient to inform on cutoffs of CRP and PCT to be used in patients with FWS in resource-poor settings. In general, there was little benefit in combining two tests in the study by van den Bruel et al [17]. However, we postulated a theoretical benefit since PCT and CRP are positive at different time-points of the disease. Currently, IMCI does not recommend antibiotic prescription for children without severe symptoms but with FWS. In order to avoid over-prescription of antibiotics in such children with a low pre-test probability of having a serious bacterial infection, we chose cutoff values with strong rule-in value, i.e. PCT 4ng/mL and CRP 80mg/L.

- 1. **How should severe malnutrition be measured? Should it be included as a danger sign?**

Severe malnutrition is recognized as an important risk factor for severe outcome from infections [84,89,143]. In a study among 10,580 pediatric admissions in Kenya severe malnutrition accounted for 21% (141/659) of invasive bacterial infections and 38% (200/533) of deaths. Eighty-one percent (959/1183) of these admissions did not meet criteria for antibiotic treatment based on other symptoms, making anthropometry or kwashiorkor the sole basis for antibiotic treatments [45]. This justifies systematic antibiotic treatment for children with fever and severe malnutrition, and possibly hospital referral.

IMCI defines severe malnutrition as severe wasting (weight for height [standing] or weight for length [lying flat] less than -3 z-scores), or mid-upper arm circumference (MUAC) less than 115 mm, or visible clinical signs of severe malnutrition [9]. The use of several signs is out of the recognition that all signs are very specific for acute severe malnutrition and that they are not necessarily overlapping. For example, in a study among 8190 hospitalized children in Kenya (359 deaths), sensitivity and specificity for subsequent inpatient death were, respectively, 46% and 91% for MUAC less than or equal to 11.5 cm, 42% and 92% for wasting, and 47% and 93% for visible severe wasting [144]. The three indices identified different subgroups of children and were independently associated with mortality.

Myatt et al. reviewed the performance of different methods for detection of severe malnutrition at community level [145]. The review concluded that weight for height/length (WFH)-based case-detection perform worse, in terms of precision, accuracy, sensitivity, and specificity than any alternative anthropometry-based method. WFH was neither found to be simple, cheap, or acceptable. MUAC in turn was found to be the best performing method, followed by WFA. One concern with using WFA is that, in populations with a high prevalence of chronic stunting, low WFA will lack specificity, as it will misclassify children with chronic stunting as having severe acute malnutrition. Interestingly, chronic stunting has increasingly been recognized as an independent risk factor for infectious complications and mortality [146–151]. Accordingly, WFA was found to be a better predictor for mortality than WFH in several studies. However, MUAC, as a measure for acute malnutrition remained the best predictor in most studies [152–155]. An important practical restraint of length measurements at peripheral level is that they likely not feasible given the short time available for consultation and the challenges involved when children are irritable and sick [145]. For example, in a survey of IMCI implementation in Bangladesh, weight measurement was done in 20% of 842 eligible children but length measurement in only 0.6% [156]. Precision of measurement by trained community health workers was found to be lowest for WFL and highest for WFA [157]. Based on the evidence summarized above, we included MUAC measurement into e-POCT for all children above 6 months and, because there is currently not enough data to recommend it as sole measurement [158], we replaced WFH with WFA using a cutoff of <-3 z-score [159]. Clinical signs for severe malnutrition (wasting, edema) were not included since there is conflicting and little data regarding the accuracy of detection of such signs by low-level health workers [160,161]. However, the inclusion of edema in areas with significant risk of severe protein energy malnutrition may be considered in the future, as only MUAC, and not WFA, is able to detect children with Kwashiorkor given the fluid retention associated with this condition [145].

- 1. **How can the diagnosis of severe dehydration be improved?**

The WHO dehydration scale currently used in IMCI has never been formally derived nor validated. In the initial IMCI evaluation studies, all signs of the WHO dehydration scale showed low sensitivity when compared to physician diagnosis (overall sensitivity 51%, ranging from 27% for general condition and 64% for thirst) [20]. The clinical signs also had low reliability when assessed by IMCI-trained low-level health workers compared to medical doctors [162]. Two small hospital-based validation studies have been carried out in Rwanda since then. In the first study including 50 patients (29 with severe dehydration), the WHO scale had an area under the curve (AUC) for moderate and severe dehydration of 0.58 for both [163]. In the second study including 127 children (17 with severe dehydration), the AUC was 0.72. The optimal cutoff of ≥2 points had a sensitivity of 68% at a specificity of 67% [164]. Several dehydration scales have been developed in high-resource-settings which include clinical and laboratory features [165–171]. Only one, the Clinical Dehydration Scale (CDS) was developed using formal measurement methodology, including the evaluation against several outcome measures [168]. The assessment of several outcomes is of importance since most studies only have used post-illness weight gain to estimate weight loss [166–171], which is not a sufficiently validated gold-standard [167]. CDS contains 4 clinical items: general appearance, eyes, mucous membranes, and tears. In emergency department populations in high-resource settings the CDS was found to be useful in predicting weight gain [172,173], the need for intravenous rehydration [172–174], need for hospitalization [172,173], and hospital length of stay [173–175]. However, it only had a moderate inter-observer reliability (k= 0.52) [173]. For resource-poor settings, the accuracy of the CDS has been assessed in 2 hospital-based studies in Rwanda [163,164]. In the first study its sensitivity was 68% and its specificity 45% for predicting 5% body weight gain (moderate dehydration) in 48 children [163]. The second study used a composite outcome of 10% body weight or death present in 8% of patients (11/140 patients). In this study the scale had an area under the curve of 0.80 [164]. No studies have assessed the feasibility and usefulness of using the CDS in outpatient resource-poor settings.

Given that the evidence for the WHO scale was poor and that for the CDS limited in terms of reliability and use in low-resource outpatient settings, we decided to use a practical “at-risk approach” using variables of the modified Vesikari severity scale [176] for gastroenteritis. This scale was validated in a multicenter study and is used to classify severity of diarrhea in clinical trials and epidemiological studies [177,178]. Based on the Vesikari scale we considered that children with ≥5 loose stools/over past 24hrs OR ≥3 loose stools/over past 24hrs and emesis OR ≥3 emesis/over past 24hrs were at risk for dehydration and offered oral fluids. If these children would be unable to drink or would experience persistent vomiting, they would be referred for further hospital-based hydration treatment.

1. **Algorithm design**

We designed a novel algorithm (e-POCT) that integrated the modifications and additional elements developed based on the structured literature searches described above. We developed a paper flowchart that summarizes e-POCT’s logic (Fig 1, main paper).

For each patient, the algorithm asks for 5 main symptoms, follow-up with additional questions if needed based on the initial input screen, recommends laboratory testing, and gives a classification and treatment recommendation (Fig 1, main paper). All medication dosages are weight-based. Like in ALMANACH, all danger signs are evaluated at the start of the algorithm, including the disease-specific danger signs. This allows rapid assessment and referral of severe patients. Since children with severe disease and positive malaria tests are at high risk of bacterial infections [45], both antibiotic and anti-malaria treatment are recommended. Whenever possible oral antibiotics were recommended for increased patient safety [179]. We also added a section on common skin diseases, including picture examples of common tropical skin infections. Skin diseases have indeed been under-addressed within IMCI since its start [20].

The electronic support tool allowed for the automated steps (Fig 1A, main paper). First, since we managed to connect the oximeter probe directly to the android support tool, the measurements of SaO2 and HR could be fed directly into the algorithm without further manual input. Second, the software calculated automatically weight-based medication dosages and reference values (HR and RR percentiles based on age and temperature, WHO growth chart percentiles). When necessary, the software allowed entering several sub-branches simultaneously. If an antibiotic was needed for two different infections, the algorithm was able to integrate the information and to recommend one antibiotic that would work for both infections, instead of one for each disease. Third, the software recommended laboratory tests based on the signs and symptoms entered on a single screen (instead of a separate screen for each suspected disease classification). Forth, the electronic algorithm integrated all clinical signs and symptoms, as well as results of all laboratory tests performed. It then provided disease classifications and treatments based on this integrated data. Overall, the use of software allowed integration of more complex data while maintaining simple input and output screens. This allowed inclusion of additional clinical and laboratory parameters that could not be handled during a routine, “manual” consultation.

1. **Discussion**

We here provide a detailed rationale for the construction of e-POCT, a novel electronic algorithm that integrates POCTs for management of febrile illnesses in under-fives in low resource settings. The algorithm is built on an IMCI backbone and maintains IMCI’s integrative approach to the management of childhood infections. In the construction of e-POCT, we sought to address IMCI’s major challenges through a structured review of key IMCI elements, and the integration of POCTs. To our knowledge, this was the most comprehensive effort to “revisit” the IMCI case management chart considering a broad range of literature that has been published since development of IMCI, including host biomarkers. This effort was further strengthened by the experience acquired through the development and evaluation of the ALMANACH algorithm [3,16,18].

The evidence retrieved allowed us to perform major useful modifications: for the identification of patients with severe illness, pulse oximetry was integrated to identify patients with hypoxemia and severe tachycardia. Cutoffs for HR and RR were updated based on recent epidemiological studies and are corrected automatically for age and temperature by the algorithm. Systematic Hb screening was added to identify children with severe anemia. e-POCT uses MUAC and WFA to improve detection of children with severe malnutrition. For the management of children with respiratory infections, we constructed a ‘severe respiratory distress’ category and added a two-step diagnostic approach for bacterial pneumonia using RR and a CRP POCTs. Bacterial infections in children with FWS were addressed through CRP and PCT POCTs. The low-evidence and complex WHO dehydration scale was replaced with a simple ‘at-risk’ approach based on 24hr parental recall. We aimed at increasing the user-friendliness through the use of an electronic format, the reduction of clinical elements, and a simple user-interface.

Overall, the design of the algorithm was limited by the scarcity of evidence of some of the key areas that required revision. This was especially true for evidence from low-resource settings. Prospective studies on the accuracy of danger signs (especially the sensitivity) are lacking. All studies that assess IMCI danger signs were hospital-based [21,22]. Hence, they can only give estimates on the specificity of danger signs. Some additional clinical signs, such as CRT or jaundice have been shown to be valid in predicting severe illness. However, their reliability when measured by low-level health workers has not been demonstrated. Since development of e-POCT, some of the pediatric severity scores have been further validated [180]. Two additional score have been developed [181,182]. However, the reliability and usefulness of the scores at peripheral health care level remains to be determined. Unfortunately, there is no clinical discriminator for anemia, requiring systematic Hb screening of all children. Given the high mortality from anemia, such an approach may be justified. However, this will likely lead to higher referral of patients and has to be met with improved hospital-based blood transfusion capacity. Furthermore, the majority of severe anemia used to occur in children with malaria [183–185]. With declining malaria transmission this proportion has decreased [186] and the usefulness of systematic Hb screening has to be assessed in low malaria transmission areas. Alternative approaches to Hb screening, such as the Hemoglobin scale may be a future option, though its accuracy had to be improved [187]. Identification of children with severe respiratory infections is limited by low-level evidence of discriminatory signs. Lower chest indrawing has been omitted as a sign for severe pneumonia since it has been demonstrated that children with severe pneumonia do not benefit from intravenous antibiotic treatment. This is, however, not surprising since a great proportion of children with severe pneumonia suffer from viral infections [188]. Further studies should evaluate predictive signs for the need of hospital-based supportive care, regardless of the microbiological etiology. For the diagnosis of bacterial pneumonia, the role of RR as a marker for pneumonia is unclear. It was constructed as a very sensitive diagnostic criterion, but more recent evidence suggests that the current WHO cutoffs are neither sensitive nor specific [99]. The validity of using RR as an initial rule-in criterion for the diagnosis of bacterial pneumonia has to be evaluated further. One reason may be that binary cutoffs over-simplify correlation of RR with pneumonia. This is why we used updated RR cutoffs, corrected for age and temperature. A prospect may be the utilization of ‘digital stethoscopes’ that can detect children with bronchiolitis who do not require CRP testing [189]. Overall, the evidence for the use of CRP to diagnose bacterial pneumonia in children was moderate. Outcome studies are lacking. A recent study in Vietnam demonstrated that CRP could reduce antibiotic prescription for respiratory infections in children [190]. However, the trial was not powered to assess clinical outcome. Newer studies have assessed the accuracy of PCT in diagnosing childhood pneumonia [116,191]. PCT is indeed a useful marker for bacterial pneumonia in adults [192]. Using PCT for detecting bacterial pneumonia in children could be considered. As for FWS, little evidence could be retrieved on the most useful cutoff for diagnosing bacterial infections in a low prevalence setting. We used higher cutoffs for both biomarkers to avoid over-treating children at low risk for bacterial infections with antibiotics. The accuracy of biomarkers depends on the time point of measurement within the course of an illness [193]. Previous efforts to improve management of children with FWS have included urine dipstick testing to detect children with UTI [18,194]. The high CRP and PCT cutoffs may miss children with UTI raising the concern for the development of renal scarring. However, recently, specialists have proposed that less aggressive testing strategies UTI, such as testing of children with several days of fever only, may be just as adequate [195,196]. Overall, obtaining clean urine samples in children in resource-limited setting is challenging. This could be considered when more detailed evidence is available. Overall, all studies on predictors reviewed were limited by the fact that they were mostly conducted in emergency department settings. This may limit the applicability of the findings for the settings targeted by e-POCT with a lower prevalence of bacterial infections. Finally, the involvement of parental judgment, which has shown to be useful in studies in high-resource-settings has not been explored for the IMCI setting [46].

**5. References for Supportive File 3**

1. Sosa A, Byarugaba DK, Amabile C, Hsueh P-R, Kariuki S, Okeke I. Antimicrobial Resistance in Developing Countries [Internet]. New York: Springer; 2010. doi:10.1007/978-0-387-89370-9

2. Risk R, Naismith H, Burnett A, Moore SE, Cham M, Unger S. Rational prescribing in paediatrics in a resource-limited setting. Arch Dis Child. 2013;98: 503–509. doi:10.1136/archdischild-2012-302987

3. Shao AF, Rambaud-Althaus C, Samaka J, Faustine AF, Perri-Moore S, Swai N, et al. New Algorithm for Managing Childhood Illness Using Mobile Technology (ALMANACH): A Controlled Non-Inferiority Study on Clinical Outcome and Antibiotic Use in Tanzania. PLoS One. 2015;10: e0132316. doi:10.1371/journal.pone.0132316

4. D’Acremont V, Kilowoko M, Kyungu E, Philipina S, Sangu W, Kahama-Maro J, et al. Beyond Malaria — Causes of Fever in Outpatient Tanzanian Children. N Engl J Med. 2014;370: 809–817. doi:10.1056/NEJMoa1214482

5. D’Acremont V, Kahama-Maro J, Swai N, Mtasiwa D, Genton B, Lengeler C. Reduction of anti-malarial consumption after rapid diagnostic tests implementation in Dar es Salaam: a before-after and cluster randomized controlled study. Malar J. 2011;10: 107. doi:10.1186/1475-2875-10-107

6. Baltzell K, Elfving K, Shakely D, Ali AS, Msellem M, Gulati S, et al. Febrile illness management in children under five years of age: a qualitative pilot study on primary health care workers’ practices in Zanzibar. Malar J. 2013;12: 37. doi:10.1186/1475-2875-12-37

7. Liu L, Oza S, Hogan D, Perin J, Rudan I, Lawn JE, et al. Global, regional, and national causes of child mortality in 2000–13, with projections to inform post-2015 priorities: an updated systematic analysis. Lancet. 2014;385: 430–440. doi:10.1016/S0140-6736(14)61698-6

8. Gove S. Integrated management of childhood illness by outpatient health workers: technical basis and overview. Bull World Health Organ. 1997;75: 7–24.

9. World Health Organization. IMCI Chart Booklet [Internet]. World Health Organization; 2014. Available: http://www.who.int/maternal_child_adolescent/documents/IMCI_chartbooklet/en/

10. Horwood C, Vermaak K, Rollins N, Haskins L, Nkosi P, Qazi S. An evaluation of the quality of IMCI assessments among IMCI trained health workers in South Africa. PLoS One. 2009;4: e5937. doi:10.1371/journal.pone.0005937

11. Baiden F, Owusu-Agyei S, Bawah J, Bruce J, Tivura M, Delmini R, et al. An evaluation of the clinical assessments of under-five febrile children presenting to primary health facilities in rural Ghana. PLoS One. 2011;6: e28944. doi:10.1371/journal.pone.0028944

12. Arifeen SE, Bryce J, Gouws E, Baqui AH, Black RE, Hoque DME, et al. Quality of care for under-fives in first-level health facilities in one district of Bangladesh. Bull World Health Organ. 2005;83: 260–267. doi:/S0042-96862005000400009

13. Lange S, Mwisongo A, Mæstad O. Why don’t clinicians adhere more consistently to guidelines for the Integrated Management of Childhood Illness (IMCI)? Soc Sci Med. 2014;104: 56–63. doi:10.1016/j.socscimed.2013.12.020

14. Thompson M, Van den Bruel A, Verbakel J, Lakhanpaul M, Haj-Hassan T, Stevens R, et al. Systematic review and validation of prediction rules for identifying children with serious infections in emergency departments and urgent-access primary care. Health Technol Assess. 2012;16: 1–100. doi:10.3310/hta16150

15. Mitchell M, Hedt-Gauthier BL, Msellemu D, Nkaka M, Lesh N. Using electronic technology to improve clinical care - results from a before-after cluster trial to evaluate assessment and classification of sick children according to Integrated Management of Childhood Illness (IMCI) protocol in Tanzania. BMC Med Inform Decis Mak. 2013;13: 95. doi:10.1186/1472-6947-13-95

16. Rambaud-Althaus C, Shao A, Samaka J, Swai N, Perri S, Kahama-Maro J, et al. Performance of Health Workers Using an Electronic Algorithm for the Management of Childhood Illness in Tanzania: A Pilot Implementation Study. Am J Trop Med Hyg. 2017;96: 249–257. doi:10.4269/ajtmh.15-0395

17. Van den Bruel A, Thompson MJ, Haj-Hassan T, Stevens R, Moll H, Lakhanpaul M, et al. Diagnostic value of laboratory tests in identifying serious infections in febrile children: systematic review. BMJ. 2011;342: d3082. doi:10.1136/bmj.d3082

18. Rambaud-Althaus C, Shao AF, Kahama-Maro J, Genton B, D’Acremont V. Managing the Sick Child in the Era of Declining Malaria Transmission: Development of ALMANACH, an Electronic Algorithm for Appropriate Use of Antimicrobials. PLoS One. 2015;10: e0127674. doi:10.1371/journal.pone.0127674

19. Weber MW, Mulholland EK, Jaffar S, Troedsson H, Gove S, Greenwood BM. Evaluation of an algorithm for the integrated management of childhood illness in an area with seasonal malaria in the Gambia. Bull World Health Organ. 1997;75 Suppl 1: 25–32.

20. Perkins B a, Zucker JR, Otieno J, Jafari HS, Paxton L, Redd SC, et al. Evaluation of an algorithm for integrated management of childhood illness in an area of Kenya with high malaria transmission. Bull World Health Organ. 1997;75 Suppl 1: 33–42.

21. Paxton LA, Redd SC, Steketee RW, Otieno JO, Nahlen B. An evaluation of clinical indicators for severe paediatric illness. Bull World Health Organ. 1996;74: 613–618.

22. Kalter HD, Schillinger JA, Hossain M, Burnham G, Saha S, de Wit V, et al. Identifying sick children requiring referral to hospital in Bangladesh. Bull World Health Organ. 1997;75 Suppl 1: 65–75.

23. World Health Organization. Paediatric emergency triage, assessment and treatment Care of critically ill children [Internet]. Geneva; 2016. doi:978 92 4 151021 9

24. Twomey M, Cheema B, Buys H, Cohen K, de Sa A, Louw P, et al. Vital signs for children at triage: A multicentre validation of the revised South African triage scale (SATS) for children. South African Med J. 2013;103: 304–308. doi:10.7196/SAMJ.6877

25. Robertson MA, Molyneux EM. Description of cause of serious illness and outcome in patients identified using ETAT guidelines in urban Malawi. Arch Dis Child. 2001;85: 214–7. doi:10.1136/adc.85.3.214

26. World Health Organization. Pocket book of hospital care for children: guidelines for the management of common childhood illnesses. 2nd ed. Geneva: World Health Organization; 2013.

27. Pollack MM, Patel KM, Ruttimann UE. PRISM III: an updated Pediatric Risk of Mortality score. Crit Care Med. 1996;24: 743–52. doi:10.1007/s13398-014-0173-7.2

28. Gerardin P, Rogier C, Leteurtre S, Jouvencel P, Ka AS, Imbert P. Evaluation of Pediatric Risk of Mortality (PRISM) scoring in African children with falciparum malaria. Pediatr Crit Care Med. 2006;7: 45–47. doi:10.1097/01.PCC.0000192321.66637.E6

29. Shann F, Pearson G, Slater A, Wilkinson K. Paediatric index of mortality (PIM): A mortality prediction model for children in intensive care. Intensive Care Med. 1997;23: 201–207. doi:10.1007/s001340050317

30. Duncan H, Hutchison J, Parshuram CS. The Pediatric Early Warning System score: a severity of illness score to predict urgent medical need in hospitalized children. J Crit Care. 2006;21: 271–8. doi:10.1016/j.jcrc.2006.06.007

31. Parshuram CS, Hutchison J, Middaugh K. Development and initial validation of the Bedside Paediatric Early Warning System score. Crit Care. 2009;13: R135. doi:10.1186/cc7998

32. Egdell P, Finlay L, Pedley DK. The PAWS score: validation of an early warning scoring system for the initial assessment of children in the emergency department. Emerg Med J. 2008;25: 745–9. doi:10.1136/emj.2007.054965

33. Marsh K, Forster D, Waruiru C, Mwangi I, Winstanley M, Marsh V, Newton C, Winstanley P, Warn P, Peshu N et al. Indicators of life-threatening malaria in African children. N Engl J Med. 1995;332: 1399–404. doi:10.1056/NEJM199505253322102

34. Shann F, Barker J, Poore P. Clinical signs that predict death in children with severe pneumonia. Pediatr Infect Dis J. 1989;8: 852–5.

35. Berkley JA. Prognostic indicators of early and late death in children admitted to district hospital in Kenya: cohort study. BMJ. 2003;326: 361–361. doi:10.1136/bmj.326.7385.361

36. Helbok R, Issifou S, Matsiegui PB, Lackner P, Missinou MA, Kombila D, et al. Simplified multi-organ dysfunction score predicts disability in African children with Plasmodium falciparum malaria. Am J Trop Med Hyg. 2006;75: 443–7.

37. Bhal S, Tygai V, Kumar N, Sreenivas V, Puliyel JM, Jm P, et al. Signs of inflammation in children that can kill (SICK score): preliminary prospective validation of a new non-invasive measure of severity-of-illness. J Postgrad Med. 2006;52: 102–5.

38. Kumar N, Thomas N, Singhal D, Puliyel JM, Sreenivas V. Triage score for severity of illness. Indian Pediatr. 2003;40: 204–10.

39. Gupta MA, Chakrabarty A, Halstead R, Sahni M, Rangasami J, Puliyel A, et al. Validation of “Signs of Inflammation in Children that Kill” (SICK) score for immediate non-invasive assessment of severity of illness. Ital J Pediatr. BioMed Central; 2010;36: 35. doi:10.1186/1824-7288-36-35

40. Helbok R, Kendjo E, Issifou S, Lackner P, Newton CR, Kombila M, et al. The Lambaréné Organ Dysfunction Score (LODS) is a simple clinical predictor of fatal malaria in African children. J Infect Dis. 2009;200: 1834–41. doi:10.1086/648409

41. Kotloff KL, Nataro JP, Blackwelder WC, Nasrin D, Farag TH, Panchalingam S, et al. Burden and aetiology of diarrhoeal disease in infants and young children in developing countries (the Global Enteric Multicenter Study, GEMS): a prospective, case-control study. Lancet. 2013;382: 209–22. doi:10.1016/S0140-6736(13)60844-2

42. De Santis O, D’Acremont V. Predictive value of clinical and laboratory features for the main febrile diseases in children living in Tanzania. PLoS One. 2017; doi:forthcoming

43. Van den Bruel A, Haj-Hassan T, Thompson M, Buntinx F, Mant D. Diagnostic value of clinical features at presentation to identify serious infection in children in developed countries: a systematic review. Lancet. 2010;375: 834–45. doi:10.1016/S0140-6736(09)62000-6

44. von Seidlein L, Olaosebikan R, Hendriksen ICE, Lee SJ, Adedoyin OT, Agbenyega T, et al. Predicting the clinical outcome of severe falciparum malaria in african children: findings from a large randomized trial. Clin Infect Dis. 2012;54: 1080–90. doi:10.1093/cid/cis034

45. Berkley J, Maitland K, Mwangi I, Ngetsa C, Mwarumba S, Lowe BS, et al. Use of clinical syndromes to target antibiotic prescribing in seriously ill children in malaria endemic area: observational study. BMJ. 2005;330: 995. doi:10.1136/bmj.38408.471991.8F

46. Van den Bruel A, Aertgeerts B, Bruyninckx R, Aerts M, Buntinx F. Signs and symptoms for diagnosis of serious infections in children: a prospective study in primary care. Br J Gen Pract. 2007;57: 538–46.

47. Thompson M, Coad N, Harnden A, Mayon-White R, Perera R, Mant D. How well do vital signs identify children with serious infections in paediatric emergency care? Arch Dis Child. 2009;94: 888–93. doi:10.1136/adc.2009.159095

48. Fleming S, Gill P, Jones C, Taylor JA, Van den Bruel A, Heneghan C, et al. Validity and reliability of measurement of capillary refill time in children: a systematic review. Arch Dis Child. 2015;100: 239–49. doi:10.1136/archdischild-2014-307079

49. Biban P, Gaffuri M, Spaggiari S, Zaglia F, Serra A, Santuz P. Early recognition and management of septic shock in children. Pediatr Rep. 2012;4: e13. doi:10.4081/pr.2012.e13

50. Fleming S, Thompson M, Stevens R, Heneghan C, Plüddemann A, MacOnochie I, et al. Normal ranges of heart rate and respiratory rate in children from birth to 18 years of age: A systematic review of observational studies. Lancet. 2011;377: 1011–1018. doi:10.1016/S0140-6736(10)62226-X

51. Nijman RG, Thompson M, van Veen M, Perera R, Moll HA, Oostenbrink R. Derivation and validation of age and temperature specific reference values and centile charts to predict lower respiratory tract infection in children with fever : BMJ. 2012;345: e4224–e4224. doi:10.1136/bmj.e4224

52. Thompson M, Harnden A, Perera R, Mayon-White R, Smith L, McLeod D, et al. Deriving temperature and age appropriate heart rate centiles for children with acute infections. Arch Dis Child. 2009;94: 361–365. doi:10.1136/adc.2008.145011

53. O’Dempsey TJ, Laurence BE, McArdle TF, Todd JE, Lamont AC, Greenwood BM. The effect of temperature reduction on respiratory rate in febrile illnesses. Arch Dis Child. 1993;68: 492–5.

54. Campbell H, Byass P, O’Dempsey TJ. Effects of body temperature on respiratory rate in young children. Arch Dis Child. 1992;67: 664.

55. Simoes E, Roark R, Berman S, Esler LL. Respiratory rate : measurement of variability over time and accuracy at different counting periods. 1991; 1199–1203.

56. Brabin BJ, Premji Z, Verhoeff F. An analysis of anemia and child mortality. J Nutr. 2001;131: 636S–645S.

57. Djelantik IGG, Gessner BD, Sutanto A, Steinhoff M, Linehan M, Moulton LH, et al. Case fatality proportions and predictive factors for mortality among children hospitalized with severe pneumonia in a rural developing country setting. J Trop Pediatr. 2003;49: 327–32.

58. Calis JCJ, Phiri KS, Faragher EB, Brabin BJ, Bates I, Cuevas LE, et al. Severe Anemia in Malawian Children. N Engl J Med. 2008;358: 888–899. doi:10.1056/NEJMoa072727

59. Chalco JP, Huicho L, Alamo C, Carreazo NY, Bada C a. Accuracy of clinical pallor in the diagnosis of anaemia in children: a meta-analysis. BMC Pediatr. 2005;5: 46. doi:10.1186/1471-2431-5-46

60. Butt Z, Ashfaq U, Sherazi SFH, Jan NU, Shahbaz U. Diagnostic accuracy of “pallor” for detecting mild and severe anaemia in hospitalized patients. J Pak Med Assoc. 2010;60: 762–765.

61. Aggarwal AK, Tripathy JP, Sharma D, Prabhu A. Validity of Palmar Pallor for Diagnosis of Anemia among Children Aged 6-59 Months in North India. Anemia. 2014;2014: 543860. doi:10.1155/2014/543860

62. Lackritz EM, Campbell CC, Ruebush TK, Hightower AW, Wakube W, Steketee RW, et al. Effect of blood transfusion on survival among children in a Kenyan hospital. Lancet. 1992;340: 524–8.

63. English M, Ahmed M, Ngando C, Berkley J, Ross A. Blood transfusion for severe anaemia in children in a Kenyan hospital. Lancet. 2002;359: 494–495. doi:10.1016/S0140-6736(02)07666-3

64. Lim Y-W, Steinhoff M, Girosi F, Holtzman D, Campbell H, Boer R, et al. Reducing the global burden of acute lower respiratory infections in children: the contribution of new diagnostics. Nature. 2006; 9–18. doi:10.1038/nature05442

65. World Health Organization. IMCI Chart Booklet. Geneva: World Health Organization; 2008.

66. Soofi S, Ahmed S, Fox MP, MacLeod WB, Thea DM, Qazi S a, et al. Effectiveness of community case management of severe pneumonia with oral amoxicillin in children aged 2-59 months in Matiari district, rural Pakistan: a cluster-randomised controlled trial. Lancet. 2012;379: 729–37. doi:10.1016/S0140-6736(11)61714-5

67. Addo-Yobo E, Anh DD, El-Sayed HF, Fox LM, Fox MP, MacLeod W, et al. Outpatient treatment of children with severe pneumonia with oral amoxicillin in four countries: the MASS study. Trop Med Int Health. 2011;16: 995–1006. doi:10.1111/j.1365-3156.2011.02787.x

68. Bari A, Sadruddin S, Khan AA, Khan IUH, Khan AA, Lehri I a, et al. Community case management of severe pneumonia with oral amoxicillin in children aged 2-59 months in Haripur district, Pakistan: a cluster randomised trial. Lancet. 2011;378: 1796–803. doi:10.1016/S0140-6736(11)61140-9

69. Hazir T, Fox LAM, Nisar Y Bin, Fox MP, Ashraf YP, MacLeod WB, et al. Ambulatory short-course high-dose oral amoxicillin for treatment of severe pneumonia in children: a randomised equivalency trial. Lancet. 2008;371: 49–56. doi:10.1016/S0140-6736(08)60071-9

70. Addo-Yobo E, Chisaka N, Hassan M, Hibberd P, Lozano JM, Jeena P, et al. Oral amoxicillin versus injectable penicillin for severe pneumonia in children aged 3 to 59 months: a randomised multicentre equivalency study. Lancet. 2004;364: 1141–8. doi:10.1016/S0140-6736(04)17100-6

71. Agweyu A, Gathara D, Oliwa J, Muinga N, Edwards T, Allen E, et al. Oral amoxicillin versus benzyl penicillin for severe pneumonia among Kenyan children: A pragmatic randomized controlled noninferiority trial. Clin Infect Dis. 2015;60. doi:10.1093/cid/ciu1166

72. Patel ABAB, Bang A, Singh M, Dhande L, Chelliah LRLR, Malik A, et al. A randomized controlled trial of hospital versus home based therapy with oral amoxicillin for severe pneumonia in children aged 3 - 59 months: The IndiaCLEN Severe Pneumonia Oral Therapy (ISPOT) Study. BMC Pediatr. 2015;15: 186. doi:10.1186/s12887-015-0510-9

73. Das RR, Singh M. Treatment of Severe Community-Acquired Pneumonia with Oral Amoxicillin in Under-Five Children in Developing Country: A Systematic Review. PLoS One. 2013;8. doi:10.1371/journal.pone.0066232

74. Nair H, Simões EAF, Rudan I, Gessner BD, Azziz-Baumgartner E, Zhang JSF, et al. Global and regional burden of hospital admissions for severe acute lower respiratory infections in young children in 2010: a systematic analysis. Lancet. 2013;381: 1380–90. doi:10.1016/S0140-6736(12)61901-1

75. Pépin J, Demers AM, Mberyo-Yaah F, Jaffar S, Blais C, Somsé P, et al. Acute lower respiratory infections among children hospitalized in Bangui, Central African Republic: toward a new case-management algorithm. Trans R Soc Trop Med Hyg. 95: 410–7.

76. Kelly MS, Smieja M, Luinstra K, Wirth KE, Goldfarb DM, Steenhoff AP, et al. Association of respiratory viruses with outcomes of severe childhood pneumonia in Botswana. PLoS One. 2015;10. doi:10.1371/journal.pone.0126593

77. Duarte-Dorado DM, Madero-Orostegui DS, Rodriguez-Martinez CE, Nino G. Validation of a scale to assess the severity of bronchiolitis in a population of hospitalized infants. J Asthma. NIH Public Access; 2013;50: 1056–61. doi:10.3109/02770903.2013.834504

78. Voets S, van Berlaer G, Hachimi-Idrissi S. Clinical predictors of the severity of bronchiolitis. Eur J Emerg Med. 2006;13: 134–138. doi:10.1097/01.mej.0000206194.85072.33

79. Chalut DS, Ducharme FM, Davis GM. The Preschool Respiratory Assessment Measure (PRAM): A responsive index of acute asthma severity. J Pediatr. 2000;137: 762–768. doi:10.1067/mpd.2000.110121

80. Conway SP, Littlewood JM. Admission to hospital with asthma. Arch Dis Child. 1985;60: 636–9.

81. Arnold DH, Gebretsadik T, Abramo TJ, Moons KG, Sheller JR, Hartert T V. The RAD score: a simple acute asthma severity score compares favorably to more complex scores. Ann Allergy Asthma Immunol. 2011;107: 22–8. doi:10.1016/j.anai.2011.03.011

82. Hurwitz ME, Burney RE, Howatt WF, Crowley D, Mackenzie JR. Clinical scoring does not accurately assess hypoxemia in pediatric asthma patients. Ann Emerg Med. 1984;13: 1040–1043. doi:10.1016/S0196-0644(84)80066-9

83. Bekhof J, Reimink R, Brand PLP. Systematic review: Insufficient validation of clinical scores for the assessment of acute dyspnoea in wheezing children. Paediatr Respir Rev. 2014;15: 98–112. doi:10.1016/j.prrv.2013.08.004

84. Reed C, Madhi SA, Klugman KP, Kuwanda L, Ortiz JR, Finelli L, et al. Development of the Respiratory Index of Severity in Children (RISC) score among young children with respiratory infections in South Africa. PLoS One. 2012;7: e27793. doi:10.1371/journal.pone.0027793

85. Emukule GO, McMorrow M, Ulloa C, Khagayi S, Njuguna HN, Burton D, et al. Predicting mortality among hospitalized children with respiratory illness in Western Kenya, 2009-2012. PLoS One. 2014;9: 2009–2012. doi:10.1371/journal.pone.0092968

86. Hooli S, Colbourn T, Lufesi N, Costello A, Nambiar B, Thammasitboon S, et al. Predicting Hospitalised Paediatric Pneumonia Mortality Risk: An External Validation of RISC and mRISC, and Local Tool Development (RISC-Malawi) from Malawi. PLoS One. 2016;11: e0168126. doi:10.1371/journal.pone.0168126

87. Wandeler G, Pauchard JY, Zangger E, Diawara H, Gehri M. Which clinical signs predict hypoxaemia in young Senegalese children with acute lower respiratory tract disease? Paediatr Int Child Heal. 2015;35: 65–68. doi:10.1179/2046905514y.0000000153

88. Demers AM, Morency P, Mberyo-Yaah F, Jaffar S, Blais C, Somsé P, et al. Risk factors for mortality among children hospitalized because of acute respiratory infections in Bangui, Central African Republic. Pediatr Infect Dis J. 2000;19: 424–32.

89. Jain DL, Sarathi V, Jawalekar S. Predictors of treatment failure in hospitalized children [3-59 months] with severe and very severe pneumonia. Indian Pediatr. 2013;50. doi:10.1007/s13312-013-0220-z

90. Mamtani M, Patel A, Hibberd PL, Tuan TA, Jeena P, Chisaka N, et al. A clinical tool to predict failed response to therapy in children with severe pneumonia. Pediatr Pulmonol. 2009;44: 379–386. doi:10.1002/ppul.21014

91. Corrard F, de La Rocque F, Martin E, Wollner C, Elbez A, Koskas M, et al. Food intake during the previous 24 h as a percentage of usual intake: a marker of hypoxia in infants with bronchiolitis: an observational, prospective, multicenter study. BMC Pediatr. 2013;13: 6. doi:10.1186/1471-2431-13-6

92. Kelly CS, Andersen CL, Pestian JP, Wenger AD, Finch AB, Strope GL, et al. Improved outcomes for hospitalized asthmatic children using a clinical pathway. Ann Allergy, Asthma Immunol. 2000;84: 509–516. doi:10.1016/S1081-1206(10)62514-8

93. Roofe LR, Resha DJ, Abramo TJ, Arnold DH. Noninvasive bedside assessment of acute asthma severity using single-breath counting. Pediatr Emerg Care. 2014;30: 8–10. doi:10.1097/PEC.0000000000000060

94. Fox MP, Thea DM, Sadruddin S, Bari A, Bonawitz R, Hazir T, et al. Low Rates of Treatment Failure in Children Aged 2-59 Months Treated for Severe Pneumonia: A Multisite Pooled Analysis. Clin Infect Dis. 2013;56: 978–987. doi:10.1093/cid/cis1201

95. McCollum EDED, King C, Hollowell R, Zhou J, Colbourn T, Nambiar B, et al. Predictors of treatment failure for non-severe childhood pneumonia in developing countries--systematic literature review and expert survey--the first step towards a community focused mHealth risk-assessment tool? BMC Pediatr. 2015;15: 74. doi:10.1186/s12887-015-0392-x

96. Westley CR, Cotton EK, Brooks JG. Nebulized racemic epinephrine by IPPB for the treatment of croup: a double-blind study. Am J Dis Child. 1978;132: 484–7.

97. Taussig LM, Castro O, Beaudry PH, Fox WW, Bureau M. Treatment of laryngotracheobronchitis (croup). Use of intermittent positive-pressure breathing and racemic epinephrine. Am J Dis Child. 1975;129: 790–3.

98. Simoes EA, McGrath EJ. Recognition of pneumonia by primary health care workers in Swaziland with a simple clinical algorithm. Lancet. 340: 1502–3.

99. Rambaud-Althaus C, Althaus F, Genton B, D’Acremont V. Clinical features for diagnosis of pneumonia in children younger than 5 years: a systematic review and meta-analysis. Lancet Infect Dis. 2015;15: 439–50. doi:10.1016/S1473-3099(15)70017-4

100. Muro F, Mtove G, Mosha N, Wangai H, Harrison N, Hildenwall H, et al. Effect of context on respiratory rate measurement in identifying non-severe pneumonia in African children. Trop Med Int Heal. 2015;20: 757–765. doi:10.1111/tmi.12492

101. English M, Murphy S, Mwangi I, Crawley J, Peshu N, Marsh K. Interobserver variation in respiratory signs of severe malaria. Arch Dis Child. 1995;72: 334–6.

102. Spruijt B, Vergouwe Y, Nijman RG, Thompson M, Oostenbrink R. Vital signs should be maintained as continuous variables when predicting bacterial infections in febrile children. J Clin Epidemiol. 2013;66: 453–457. doi:10.1016/j.jclinepi.2012.09.014

103. Madhi SA, Klugman KP. World Health Organisation definition of “radiologically-confirmed pneumonia” may under-estimate the true public health value of conjugate pneumococcal vaccines. Vaccine. 2007;25: 2413–2419. doi:10.1016/j.vaccine.2006.09.010

104. Díez-Padrisa N, Bassat Q, Machevo S, Quintó L, Morais L, Nhampossa T, et al. Procalcitonin and C-Reactive Protein for Invasive Bacterial Pneumonia Diagnosis among Children in Mozambique, a Malaria-Endemic Area. Ratner AJ, editor. PLoS One. 2010;5: e13226. doi:10.1371/journal.pone.0013226

105. Flood RG, Badik J, Aronoff SC. The Utility of Serum C-Reactive Protein in Differentiating Bacterial from Nonbacterial Pneumonia in Children. Pediatr Infect Dis J. 2008;PAP: 95–9. doi:10.1097/INF.0b013e318157aced

106. Sanders S, Barnett A, Correa-Velez I, Coulthard M, Doust J. Systematic review of the diagnostic accuracy of C-reactive protein to detect bacterial infection in nonhospitalized infants and children with fever. J Pediatr. 2008;153: 570–4. doi:10.1016/j.jpeds.2008.04.023

107. Lynch T, Bialy L, Kellner JD, Osmond MH, Klassen TP, Durec T, et al. A systematic review on the diagnosis of pediatric bacterial pneumonia: When gold is bronze. PLoS One. 2010;5. doi:10.1371/journal.pone.0011989

108. Mulholland K, Hilton S, Adegbola R, Usen S, Oparaugo A, Omosigho C, et al. Randomised trial of Haemophilus influenzae type-b tetanus protein conjugate vaccine [corrected] for prevention of pneumonia and meningitis in Gambian infants. Lancet. 1997;349: 1191–7. doi:10.1016/S0140-6736(96)09267-7

109. Levine OS, Lagos R, Muñoz A, Villaroel J, Alvarez AM, Abrego P, et al. Defining the burden of pneumonia in children preventable by vaccination against Haemophilus influenzae type b. Pediatr Infect Dis J. 1999;18: 1060–4.

110. Cutts F, Zaman S, Enwere G, Jaffar S, Levine O, Okoko J, et al. Efficacy of nine-valent pneumococcal conjugate vaccine against pneumonia and invasive pneumococcal disease in The Gambia: randomised, double-blind, placebo-controlled trial. Lancet. 2005;365: 1139–1146. doi:10.1016/S0140-6736(05)71876-6

111. Babu G, Ganguly NK, Singhi S, Walia BN. Value of C-reactive protein concentration in diagnosis and management of acute lower respiratory infections. Trop Geogr Med. 1989;41: 309–15.

112. Page A-L, de Rekeneire N, Sayadi S, Aberrane S, Janssens A-C, Rieux C, et al. Infections in children admitted with complicated severe acute malnutrition in Niger. PLoS One. 2013;8: e68699. doi:10.1371/journal.pone.0068699

113. Koster MJ, Broekhuizen BDL, Minnaard MC, Balemans WAF, Hopstaken RM, de Jong PA, et al. Diagnostic properties of C-reactive protein for detecting pneumonia in children. Respir Med. 2013;107: 1087–1093. doi:10.1016/j.rmed.2013.04.012

114. Erdman LK, D’Acremont V, Hayford K, Rajwans N, Kilowoko M, Kyungu E, et al. Biomarkers of host response predict primary end-point radiological pneumonia in Tanzanian children with clinical pneumonia: A prospective cohort study. PLoS One. 2015;10: e0137592. doi:10.1371/journal.pone.0137592

115. Galetto-Lacour A, Alcoba G, Posfay-Barbe KM, Cevey-Macherel M, Gehri M, Ochs MM, et al. Elevated inflammatory markers combined with positive pneumococcal urinary antigen are a good predictor of pneumococcal community-acquired pneumonia in children. Pediatr Infect Dis J. 2013;32: 1175–9. doi:10.1097/INF.0b013e31829ba62a

116. Agnello L, Bellia C, Di Gangi M, Lo Sasso B, Calvaruso L, Bivona G, et al. Utility of serum procalcitonin and C-reactive protein in severity assessment of community-acquired pneumonia in children. Clin Biochem. 2016;49: 47–50. doi:10.1016/j.clinbiochem.2015.09.008

117. Elemraid MA, Rushton SP, Thomas MF, Spencer DA, Gennery AR, Clark JE. Utility of inflammatory markers in predicting the aetiology of pneumonia in children. Diagn Microbiol Infect Dis. 2014;79: 458–462. doi:10.1016/j.diagmicrobio.2014.04.006

118. Myers AL, Hall M, Williams DJ, Auger K, Tieder JS, Statile A, et al. Prevalence of bacteremia in hospitalized pediatric patients with community-acquired pneumonia. Pediatr Infect Dis J. 2013;32: 736–740. doi:10.1097/INF.0b013e318290bf63

119. Yadav KK, Awasthi S, Takia L, Agarwal J, Agarwal GG. Procalcitonin and C-reactive protein in WHO defined severe and very severe community acquired pneumonia: A hospital based cross-sectional study. Clin Epidemiol Glob Heal. 2015;3: S3–S9. doi:10.1016/j.cegh.2015.11.005

120. Toikka P, Irjala K, Juven T, Virkki R, Mertsola J, Leinonen M, et al. Serum procalcitonin, C-reactive protein and interleukin-6 for distinguishing bacterial and viral pneumonia in children. Pediatr Infect Dis J. 2000;19: 598–602. doi:10.1097/00006454-200007000-00003

121. Leroy S, Romanello C, Galetto-Lacour A, Smolkin V, Korczowski B, Rodrigo C, et al. Procalcitonin to reduce the number of unnecessary cystographies in children with a urinary tract infection: a European validation study. J Pediatr. 2007;150: 89–95. doi:10.1016/j.jpeds.2006.08.066

122. Moulin F, Raymond J, Lorrot M, Marc E, Coste J, Iniguez JL, et al. Procalcitonin in children admitted to hospital with community acquired pneumonia. Arch Dis Child. 2001;84: 332–6. doi:10.1136/ADC.84.4.332

123. Heiskanen-Kosma T, Korppi M. Serum C-reactive protein cannot differentiate bacterial and viral aetiology of community-acquired pneumonia in children in primary healthcare settings. Scand J Infect Dis. 2000;32: 399–402.

124. Korppi M, Remes S. Serum procalcitonin in pneumococcal pneumonia in children. Eur Respir J. 2001;17: 623–627.

125. Nascimento-Carvalho CM, Cardoso MR, Barral A, Araujo-Neto CA, Guerin S, Saukkoriipi A, et al. Procalcitonin is useful in identifying bacteraemia among children with pneumonia. Scand J Infect Dis. 2010;42: 644–649. doi:10.3109/00365541003796775

126. Korppi M, Remes S, Heiskanen-Kosma T. Serum procalcitonin concentrations in bacterial pneumonia in children: A negative result in primary healthcare settings. Pediatr Pulmonol. 2003;35: 56–61. doi:10.1002/ppul.10201

127. Cohen JF, Leis A, Lecarpentier T, Raymond J, Gendrel D, Chalumeau M. Procalcitonin Predicts Response to beta-lactam treatment in hospitalized children with community-acquired pneumonia. PLoS One. 2012;7: 3–7. doi:10.1371/journal.pone.0036927

128. Prat C, Domínguez J, Rodrigo C, Giménez M, Azuara M, Jiménez O, et al. Procalcitonin, C-reactive protein and leukocyte count in children with lower respiratory tract infection. Pediatr Infect Dis J. 2003;22: 963–8. doi:10.1097/01.inf.0000095197.72976.4f

129. MCGowan JE, Bratton L, Klein JO, Finland M. Bacteremia in Febrile Children Seen in a Walk-in Pediatric Clinic. N Engl J Med. 1973;288: 1309–1312. doi:10.1056/NEJM197306212882501

130. Baron MA, Fink HD, Posnett J, Sandler T. Bacteremia in Private Pediatric Practice. Pediatrics. 1980;66: 171 LP-175.

131. Bressan S, Berlese P, Mion T, Masiero S, Cavallaro A, Da Dalt L. Bacteremia in feverish children presenting to the emergency department: a retrospective study and literature review. Acta Paediatr. 2012;101: 271–7. doi:10.1111/j.1651-2227.2011.02478.x

132. Hernandez-Bou S, Trenchs V, Batlle A, Gene A, Luaces C. Occult bacteraemia is uncommon in febrile infants who appear well, and close clinical follow-up is more appropriate than blood tests. Acta Paediatr. 2015;104: e76–e81. doi:10.1111/apa.12852

133. Shaikh N, Morone NE, Bost JE, Farrell MH. Prevalence of urinary tract infection in childhood: a meta-analysis. Pediatr Infect Dis J. 2008;27: 302–8. doi:10.1097/INF.0b013e31815e4122

134. Roberts KB. Urinary tract infection: clinical practice guideline for the diagnosis and management of the initial UTI in febrile infants and children 2 to 24 months. Pediatrics. 2011;128: 595–610. doi:10.1542/peds.2011-1330

135. Whiting P, Westwood M, Watt I, Cooper J, Kleijnen J. Rapid tests and urine sampling techniques for the diagnosis of urinary tract infection (UTI) in children under five years: a systematic review. BMC Pediatr. 2005;5: 4. doi:10.1186/1471-2431-5-4

136. Mori R, Yonemoto N, Fitzgerald a, Tullus K, Verrier-Jones K, Lakhanpaul M. Diagnostic performance of urine dipstick testing in children with suspected UTI: a systematic review of relationship with age and comparison with microscopy. Acta Paediatr. 2010;99: 581–4. doi:10.1111/j.1651-2227.2009.01644.x

137. Islam K, Sayeed MA, Hossen E, Khanam F, Charles RC, Andrews J, et al. Comparison of the Performance of the TPTest, Tubex, Typhidot and Widal Immunodiagnostic Assays and Blood Cultures in Detecting Patients with Typhoid Fever in Bangladesh, Including Using a Bayesian Latent Class Modeling Approach. PLoS Negl Trop Dis. 2016;10: 1–10. doi:10.1371/journal.pntd.0004558

138. World Health Organization. WHO Child Growth Standards. Geneva: World Health Organization; 2006.

139. Shaikh N, Borrell JL, Evron J, Leeflang MMG. Procalcitonin, C-reactive protein, and erythrocyte sedimentation rate for the diagnosis of acute pyelonephritis in children. Cochrane database Syst Rev. 2015; CD009185. doi:10.1002/14651858.CD009185.pub2

140. Meloni GF, Tomasi PA, Spanu P, Piga S, Porcu A. C-reactive protein levels for diagnosis of Salmonella gastroenteritis. Pediatr Infect Dis J. 1999;18: 471–3.

141. Choo KE, Davis TM, Henry RL, Chan LP. Serum C-reactive protein concentrations in Malaysian children with enteric fever. J Trop Pediatr. 2001;47: 211–214.

142. Cooper EC, Ratnam I, Mohebbi M, Leder K. Laboratory features of common causes of fever in returned travelers. J Travel Med. 2014;21: 235–239. doi:10.1111/jtm.12122

143. Gowraiah V, Awasthi S, Kapoor R, Sahana D, Venkatesh P, Gangadhar B, et al. Can we distinguish pneumonia from wheezy diseases in tachypnoeic children under low-resource conditions? A prospective observational study in four Indian hospitals. Arch Dis Child. 2014;99: 899–906. doi:10.1136/archdischild-2013-305740

144. Berkley J, Mwangi I, Griffiths K, Ahmed I, Mithwani S, English M, et al. Assessment of severe malnutrition among hospitalized children in rural Kenya: comparison of weight for height and mid upper arm circumference. JAMA. 2005;294: 591–7. doi:10.1001/jama.294.5.591

145. Myatt M, Khara T, Collins S. A review of methods to detect cases of severely malnourished children in the community for their admission into community-based therapeutic care programs. Food Nutr Bull. 2006;27: S7-23.

146. Smedman L, Sterky G, Mellander L, Wall S. Anthropometry and subsequent mortality in groups of children aged 6-59 months in Guinea-Bissau. Am J Clin Nutr. 1987;46: 369–73.

147. Pelletier DL, Frongillo EA, Schroeder DG, Habicht JP, Habicht JP. The effects of malnutrition on child mortality in developing countries. Bull World Health Organ. 1995;73: 443–8.

148. Olofin I, McDonald CM, Ezzati M, Flaxman S, Black RE, Fawzi WW, et al. Associations of suboptimal growth with all-cause and cause-specific mortality in children under five years: a pooled analysis of ten prospective studies. PLoS One. 2013;8: e64636. doi:10.1371/journal.pone.0064636

149. Kossmann J, Nestel P, Herrera MG, El Amin A, Fawzi WW. Undernutrition in relation to childhood infections: a prospective study in the Sudan. Eur J Clin Nutr. 2000;54: 463–72.

150. Black RE, Allen LH, Bhutta ZA, Caulfield LE, de Onis M, Ezzati M, et al. Maternal and child undernutrition: global and regional exposures and health consequences. Lancet. 2008;371: 243–260. doi:10.1016/S0140-6736(07)61690-0

151. Prendergast AJ, Humphrey JH. The stunting syndrome in developing countries. Paediatr Int Child Health. 2014;34: 250–65. doi:10.1179/2046905514Y.0000000158

152. Chen LC, Chowdhury A, Huffman SL. Anthropometric assessment of energy-protein malnutrition and subsequent risk of mortality among preschool aged children. Am J Clin Nutr. 1980;33: 1836–45.

153. Briend A, Zimicki S. Validation of arm circumference as an indicator of risk of death in one to four year old children. Nutr Res. 1986;6: 249–261. doi:10.1016/S0271-5317(86)80129-4

154. Vella V, Tomkins A, Ndiku J, Marshal T, Cortinovis I. Anthropometry as a predictor for mortality among Ugandan children, allowing for socio-economic variables. Eur J Clin Nutr. 1994;48: 189–97.

155. Alam N, Wojtyniak B, Rahaman MM. Anthropometric indicators and risk of death. Am J Clin Nutr. 1989;49: 884–8.

156. Saha KK, Billah M, Menon P, Arifeen S El, Mbuya N. Bangladesh National Nutrition Services : assessment of implementation status. Washington: World Bank Group; 2015.

157. Velzeboer MI, Selwyn BJ, Sargent F, Pollitt E, Delgado H. The use of arm circumference in simplified screening for acute malnutrition by minimally trained health workers. J Trop Pediatr. 1983;29: 159–66.

158. Roberfroid D, Hammami N, Lachat C, Prinzo ZW, Sibson V, Guesdon B, et al. Utilization of mid-upper arm circumference versus weight-for-height in nutritional rehabilitation programmes: a systematic review of evidence [Internet]. Geneva; 2013. Available: http://www.who.int/entity/nutrition/publications/guidelines/updates_management_SAM_infantandchildren_review1.pdf

159. Center for Disease Control, World Food Programme. A Manual: Measuring and Interpreting Malnutrition and Mortality. Atlanta; 2005.

160. Hamer C, Kvatum K, Jeffries D, Allen S. Detection of severe protein-energy malnutrition by nurses in The Gambia. Arch Dis Child. 2004;89: 181–4.

161. Mogeni P, Twahir H, Bandika V, Mwalekwa L, Thitiri J, Ngari M, et al. Diagnostic performance of visible severe wasting for identifying severe acute malnutrition in children admitted to hospital in Kenya. Bull World Health Organ. 2011;89: 900–906. doi:10.2471/BLT.11.091280

162. Simoes EA, Desta T, Tessema T, Gerbresellassie T, Dagnew M, Gove S. Performance of health workers after training in integrated management of childhood illness in Gondar, Ethiopia. Bull World Health Organ. 1997;75 Suppl 1: 43–53.

163. Pringle K, Shah SP, Umulisa I, Munyaneza RBM, Dushimiyimana JM, Stegmann K, et al. Comparing the accuracy of the three popular clinical dehydration scales in children with diarrhea. Int J Emerg Med. 2011;4: 58. doi:10.1186/1865-1380-4-58

164. Levine AC, Munyaneza RM, Glavis-Bloom J, Redditt V, Cockrell HC, Kalimba B, et al. Prediction of severe disease in children with diarrhea in a resource-limited setting. PLoS One. 2013;8: 4–13. doi:10.1371/journal.pone.0082386

165. Falszewska A, Dziechciarz P, Szajewska H. The diagnostic accuracy of clinical dehydration scale in identifying dehydration in children with acute gastroenteritis: a systematic review. Clin Pediatr (Phila). 2014;53: 1181–8. doi:10.1177/0009922814538493

166. Steiner MJ, DeWalt DA, Byerley JS. Is this child dehydrated? JAMA. 2004;291: 2746–2754. doi:10.1001/jama.291.22.2746

167. Gorelick MH, Shaw KN, Murphy KO. Validity and reliability of clinical signs in the diagnosis of dehydration in children. Pediatrics. 1997;99: E6.

168. Friedman JN, Goldman RD, Srivastava R, Parkin PC. Development of a clinical dehydration scale for use in children between 1 and 36 months of age. J Pediatr. 2004;145: 201–207. doi:http://dx.doi.org/10.1016/j.jpeds.2004.05.035

169. Vega RM, Avner JR. A prospective study of the usefulness of clinical and laboratory parameters for predicting percentage of dehydration in children. Pediatr Emerg Care. 1997;13: 179–182.

170. Duggan C, Refat M, Hashem M, Wolff M, Fayad I, Santosham M, et al. How valid are clinical signs of dehydration in infants? J Pediatr Gastroenterol Nutr. 1996;22: 56–61.

171. Mackenzie A, Barnes G, Shann F. Clinical signs of dehydration in children. Lancet. 1989;2: 605–7.

172. Gravel J, Manzano S, Guimont C, Lacroix L, Gervaix A, Bailey B. Validation multicentrique du score clinique de deshydratation pediatrique. Arch Pediatr. 2010;17: 1645–1651. doi:10.1016/j.arcped.2010.09.009

173. Kinlin LM, Freedman SB. Evaluation of a clinical dehydration scale in children requiring intravenous rehydration. Pediatrics. 2012;129: e1211-9. doi:10.1542/peds.2011-2985

174. Goldman RD, Friedman JN, Parkin PC. Validation of the Clinical Dehydration Scale for Children With Acute Gastroenteritis. Pediatrics. 2008;122: 545–549. doi:10.1542/peds.2007-3141

175. Bailey B, Gravel J, Goldman RD, Friedman JN, Parkin PC. External validation of the clinical dehydration scale for children with acute gastroenteritis. Acad Emerg Med. 2010;17: 583–588. doi:10.1111/j.1553-2712.2010.00767.x

176. Ruuska T, Vesikari T. Rotavirus disease in Finnish children: use of numerical scores for clinical severity of diarrhoeal episodes. Scand J Infect Dis. 1990;22: 259–67. doi:10.3109/00365549009027046

177. Schnadower D, Tarr PI, Gorelick MH, O’Connell K, Roskind CG, Powell EC, et al. Validation of the modified Vesikari score in children with gastroenteritis in 5 US emergency departments. J Pediatr Gastroenterol Nutr. 2013;57: 514–9. doi:10.1097/MPG.0b013e31829ae5a3

178. Freedman SB, Eltorky M, Gorelick M. Evaluation of a gastroenteritis severity score for use in outpatient settings. Pediatrics. 2010;125: e1278–e1285. doi:10.1542/peds.2009-3270

179. Li HK, Agweyu A, English M, Bejon P. An Unsupported Preference for Intravenous Antibiotics. PLoS Med. 2015;12. doi:10.1371/journal.pmed.1001825

180. Conroy AL, Hawkes M, Hayford K, Namasopo S, Opoka RO, John CC, et al. Prospective validation of pediatric disease severity scores to predict mortality in Ugandan children presenting with malaria and non-malaria febrile illness. Crit Care. 2015;19: 47. doi:10.1186/s13054-015-0773-4

181. George EC, Walker AS, Kiguli S, Olupot-Olupot P, Opoka RO, Engoru C, et al. Predicting mortality in sick African children: the FEAST Paediatric Emergency Triage (PET) Score. BMC Med. 2015;13: 174. doi:10.1186/s12916-015-0407-3

182. Mpimbaza A, Sears D, Sserwanga A, Kigozi R, Rubahika D, Nadler A, et al. Admission Risk Score to Predict Inpatient Pediatric Mortality at Four Public Hospitals in Uganda. PLoS One. 2015;10: e0133950. doi:10.1371/journal.pone.0133950

183. Koram KA, Owusu-Agyei S, Utz G, Binka FN, Baird JK, Hoffman SL, et al. Severe anemia in young children after high and low malaria transmission seasons in the Kassena-Nankana district of northern Ghana. Am J Trop Med Hyg. 2000;62: 670–4.

184. Owusu-Agyei S, Fryauff DJ, Chandramohan D, Koram KA, Binka FN, Nkrumah FK, et al. Characteristics of severe anemia and its association with malaria in young children of the Kassena-Nankana District of northern Ghana. Am J Trop Med Hyg. 2002;67: 371–377.

185. Biemba G, Dolmans D, Thuma PE, Weiss G, Gordeuk VR. Severe anaemia in Zambian children with Plasmodium falciparum malaria. Trop Med Int Health. 2000;5: 9–16.

186. Pedro R, Akech S, Maitland K, Maitland K. Changing trends in blood transfusion in children and neonates admitted in Kilifi District Hospital, Kenya. Malar J. 2010;9: 307. doi:10.1186/1475-2875-9-307

187. Marn H, Critchley JA. Accuracy of the WHO Haemoglobin Colour Scale for the diagnosis of anaemia in primary health care settings in low-income countries: A systematic review and meta-analysis. Lancet Glob Heal. 2016;4: e251–e265. doi:10.1016/S2214-109X(16)00005-X

188. Ali A, Khowaja AR, Bashir MZ, Aziz F, Mustafa S, Zaidi A. Role of Human Metapneumovirus, Influenza A Virus and Respiratory Syncytial Virus in Causing WHO-Defined Severe Pneumonia in Children in a Developing Country. PLoS One. 2013;8: 8–11. doi:10.1371/journal.pone.0074756

189. Simoes EA, McGrath EJ, Jl J, Hf H. Recognition of pneumonia by primary health care workers in Swaziland with a simple clinical algorithm. Lancet. 1991;340: 1502–3.

190. Do NTT, Ta NTD, Tran NTH, Than HM, Vu BTN, Hoang LB, et al. Point-of-care C-reactive protein testing to reduce inappropriate use of antibiotics for non-severe acute respiratory infections in Vietnamese primary health care: a randomised controlled trial. Lancet Glob Heal. 2016;4: e633–e641. doi:10.1016/S2214-109X(16)30142-5

191. Bivona G, Luisa A, Concetta S, Bruna LS, Bellia C, Marcello C. Procalcitonin and community-acquired pneumonia (CAP) in children. Clin Chim Acta. 2015;451: 215–218. doi:10.1016/j.cca.2015.09.031

192. Shaddock EJ. How and when to use common biomarkers in community-acquired pneumonia. Pneumonia. Pneumonia; 2016;8: 17. doi:10.1186/s41479-016-0017-7

193. Melbye H, Hvidsten D, Holm A, Nordbø SA, Brox J. The course of C-reactive protein response in untreated upper respiratory tract infection. Br J Gen Pract. 2004;54: 653–8.

194. World Health Organization. Urinary Tract Infections in Infants and Children in Developing Countries in the Context of IMCI. Geneva; 2005.

195. Salleeh H Bin, Mcgillivray D, Martin M, Patel H. Duration of Fever Affects the Likelihood of a Positive Bag Urinalysis or Catheter Culture in Young Children. J Pediatr. 2010;156: 629–633. doi:10.1016/j.jpeds.2009.10.016

196. Newman DH, Shreves AE, Runde DP. Pediatric urinary tract infection: Does the evidence support aggressively pursuing the diagnosis? Ann Emerg Med. 2013;61: 559–565. doi:10.1016/j.annemergmed.2012.10.034
